# Resolving the ancestry of Austronesian-speaking populations

**DOI:** 10.1007/s00439-015-1620-z

**Published:** 2016-01-18

**Authors:** Pedro A. Soares, Jean A. Trejaut, Teresa Rito, Bruno Cavadas, Catherine Hill, Ken Khong Eng, Maru Mormina, Andreia Brandão, Ross M. Fraser, Tse-Yi Wang, Jun-Hun Loo, Christopher Snell, Tsang-Ming Ko, António Amorim, Maria Pala, Vincent Macaulay, David Bulbeck, James F. Wilson, Leonor Gusmão, Luísa Pereira, Stephen Oppenheimer, Marie Lin, Martin B. Richards

**Affiliations:** Department of Biology, CBMA (Centre of Molecular and Environmental Biology), University of Minho, Campus de Gualtar, 4710-057 Braga, Portugal; IPATIMUP (Institute of Molecular Pathology and Immunology of the University of Porto), Rua Dr. Roberto Frias s/n, 4200-465 Porto, Portugal; Faculty of Biological Sciences, University of Leeds, Leeds, LS2 9JT UK; Molecular Anthropology and Transfusion Medicine Research Laboratory, Mackay Memorial Hospital, Taipei City, 10449 Taiwan; Life and Health Sciences Research Institute (ICVS), School of Health Sciences, University of Minho, Campus de Gualtar, 4710-057 Braga, Portugal; ICVS/3B’s-PT Government Associate Laboratory, Braga, Guimarães, Portugal; I3S - Institute for Research Innovation in Health, University of Porto, 4200-135 Porto, Portugal; Centre for Global Archaeological Research, Universiti Sains Malaysia (USM), 11800 Penang, Malaysia; Department of Applied Social Studies, University of Winchester, Sparkford Road, Winchester, SO22 4NR UK; Department of Biological Sciences, School of Applied Sciences, University of Huddersfield, Queensgate, Huddersfield, HD1 3DH UK; ICBAS - Institute of Biomedical Sciences Abel Salazar, University of Porto, 4050-313 Porto, Portugal; Centre for Global Health Research, Usher Institute of Population Health Sciences and Informatics, University of Edinburgh, Teviot Place, Edinburgh, EH8 9AG Scotland, UK; Synpromics Ltd, Nine Edinburgh Bioquarter, Edinburgh, EH16 4UX UK; Department of Obstetrics and Gynecology, National Taiwan University, Roosevelt Rd., Taipei, 10617 Taiwan; Faculty of Sciences, University of Porto, Rua do Campo Alegre, s/n, 4169-007 Porto, Portugal; Department of Statistics, University of Glasgow, 15 University Gardens, Glasgow, G12 8QQ UK; Department of Archaeology and Natural History, College of Asia and the Pacific, The Australian National University, Acton, Canberra, ACT 2601 Australia; MRC Human Genetics Unit, Institute of Genetics and Molecular Medicine, University of Edinburgh, Western General Hospital, Edinburgh, EH4 2XU Scotland; DNA Diagnostic Laboratory (LDD), State University of Rio de Janeiro (UERJ), Rua São Francisco Xavier, Rio de Janeiro, 20550-900 Brazil; Faculty of Medicine, University of Porto, Al. Prof. Hernâni Monteiro, 4200-319 Porto, Portugal; Institute of Human Sciences, School of Anthropology, University of Oxford, The Pauling Centre, 58a Banbury Road, Oxford, OX2 6QS UK

## Abstract

There are two very different interpretations of the prehistory of Island Southeast Asia (ISEA), with genetic evidence invoked in support of both. The “out-of-Taiwan” model proposes a major Late Holocene expansion of Neolithic Austronesian speakers from Taiwan. An alternative, proposing that Late Glacial/postglacial sea-level rises triggered largely autochthonous dispersals, accounts for some otherwise enigmatic genetic patterns, but fails to explain the Austronesian language dispersal. Combining mitochondrial DNA (mtDNA), Y-chromosome and genome-wide data, we performed the most comprehensive analysis of the region to date, obtaining highly consistent results across all three systems and allowing us to reconcile the models. We infer a primarily common ancestry for Taiwan/ISEA populations established before the Neolithic, but also detected clear signals of two minor Late Holocene migrations, probably representing Neolithic input from both Mainland Southeast Asia and South China, via Taiwan. This latter may therefore have mediated the Austronesian language dispersal, implying small-scale migration and language shift rather than large-scale expansion.

**Electronic supplementary material:**

The online version of this article (doi:10.1007/s00439-015-1620-z) contains supplementary material, which is available to authorized users.

## Introduction

Austronesian languages are spoken throughout Taiwan, Island Southeast Asia (ISEA), parts of New Guinea and most of the Pacific Islands. The high linguistic diversity observed in the aboriginal groups of Taiwan, compared to the single language branch (Malayo-Polynesian) spoken throughout the remainder of this vast distribution (Ross 2005), has suggested to historical linguists a homeland on the island of Taiwan (Blust 1976, 1995). The Taiwanese linguistic homeland model has received further support in recent years from the work of Ross (2009), which effectively nests Malayo-Polynesian within the Formosan language tree, and has led in turn to the prevailing “out-of-Taiwan” model (Bellwood 1997; Hung et al. 2011) for the spread by demic diffusion of farming—referring specifically to rice agriculture—and red-slipped pottery from 4000 years ago (4 ka) in ISEA, culminating in the spread of Austronesian Oceanic speakers into the Pacific within the last 3 ka (Bellwood 1997; Spriggs 2003, 2007). In practice, the Neolithic in ISEA is defined by the appearance of ceramics, and less ubiquitously new shell artefacts and cloth and barkcloth technologies, and any role of rice or other introduced agriculture has proved much more contentious (Spriggs 2011).

The “out-of-Taiwan” model has been dominant for 30 years, despite challenges on many fronts. Languages can be transmitted horizontally, so a root in Taiwan need not automatically imply a demic diffusion model. More complex pictures are emerging for ISEA, in which coastal language shift and language transmissions play the major role (Donohue and Denham 2010, 2015). This paves the way for a modified small-scale “out-of-Taiwan” model that retains the linguistic argument for the origin of the Austronesian family in Taiwan without assuming any large-scale population movement or replacement, or that rice agriculture is the driving force (Diamond and Bellwood 2003; Oppenheimer 2004; Spriggs 2011).

In fact, a key driver of human mobility may have been the dramatic transformation in the landscape of ISEA in the late Pleistocene/early Holocene. Sea-level rises due to global warming at the end of the last glaciation separated the ancient Sunda continent—for millennia an extension of mainland Asia—into present-day ISEA and Mainland Southeast Asia (MSEA). These are thought to have been concentrated in three major episodes, from 15 to 13.5 ka, 11.5 to 10 ka and 7 to 8 ka (Pelejero et al. 1999). Alternative models to “out-of-Taiwan” have argued that it may have been the rapid coastal transformation and resulting land-loss (Solheim 2006) that had the most profound effect on genetic patterns in the region, rather than a more recent expansion from Taiwan (Oppenheimer 1998; Oppenheimer and Richards 2001; Soares et al. 2008, 2011) However, although such models attempt to explain the current population structure in ISEA, they have been less successful in incorporating the linguistic evidence suggesting an Austronesian origin in Taiwan (Barker and Richards 2013).

On the genetic side, many seemingly contradictory results have been published in recent years, shifting the perspective back and forth between a strong Neolithic expansion and minor or non-existent dispersals from Taiwan. The two-layer colonization model (Pleistocene colonization and mid-Holocene “out-of-Taiwan” expansion) (Bellwood 1997) often remains the lens through which data are interpreted. Thus, genetic variation is often categorised either as autochthonous (first colonization or “Melanesian”) or as a later Asian input interpreted as “Austronesian” (Friedlaender et al. 2008; Kayser et al. 2008b). The “Asian” signal on the mtDNA is generated by the so-called “Polynesian motif” (Delfin et al. 2012; Melton et al. 1995; Redd et al. 1995; Soares et al. 2011; Sykes et al. 1995; Trejaut et al. 2005), which approaches 100 % in many Remote Pacific islands.

However, Soares et al. (2011) estimated an arrival of this clade (or its ancestor) in Near Oceania ~6 to 10 ka. Although the motif has an ultimately mainland Asian ancestry (in haplogroup B4a1a) sometime in the last 10–20 ka (Soares et al. 2011), it was already well established in Near Oceania by the mid-Holocene. This implies that the contribution of a Neolithic “out-of-Taiwan” migration to Remote Pacific Islanders is negligible in the mtDNA [as well as Y-chromosome (Capelli et al. 2001; Kayser et al. 2000)] variation in the last 3 ka. But, for ISEA, too, the picture is far from consistent with an “out-of-Taiwan” demic expansion. The largest surveys consistently suggest a far more complex picture than the two-layer model (Capelli et al. 2001; Hill et al. 2007; Karafet et al. 2010; Trejaut et al. 2014; Tumonggor et al. 2013). Sea-level rises probably shaped much of the genetic structure of ISEA (Hill et al. 2007; Karafet et al. 2010), with major dispersals originating in what is now the mainland [including mtDNA haplogroup B4a1a (Soares et al. 2011)] as well as across what is now ISEA [including haplogroup E (Soares et al. 2008)].

Genome-wide data have also led to challenges to the “out-of-Taiwan” model, albeit with caveats (discussed below). The Pan-Asian SNP Consortium (Abdulla et al. 2009) suggested that the diversity of Taiwanese aboriginals is likely a sub-set of the ISEA diversity, implying that dispersals between Taiwan and ISEA took place in the reverse direction. This would match the situation seen in mtDNA haplogroup E, inferred to have expanded in ISEA in the postglacial period and reached Taiwan within the last 8 ka (Soares et al. 2008).

Here we perform founder analyses with large new mtDNA datasets, both control-region and whole-genome sequences, and—for the first time—Y-chromosome data. Founder analysis estimates dispersal times and quantifies the contribution of each migration to the present-day population. We develop an explicit set of criteria by which to evaluate candidate “out-of-Taiwan” markers, and show that haplogroup M7c3, analysed here at the maximal resolution level of whole-mtDNAs, and found in aboriginal Taiwanese and the Philippines at moderate frequencies, but only low frequencies in ISEA and the western Pacific, fulfils these criteria almost perfectly. However, the other major candidates proposed for the “out-of-Taiwan” dispersal, haplogroups E and B4a1a, fail to meet any of them.

Single-locus studies of the uni-parental marker systems can today provide exquisite resolution, but they are, of course, subject to greater stochastic effects than the autosomal genome. We therefore here back up mtDNA and Y-chromosome variation with fresh analyses of the autosomal genome-wide structure of Southeast Asians. These multi-locus analyses support the view that the spread of the red-slipped pottery Neolithic and Austronesian languages in ISEA were indeed accompanied by dispersals of sea-farers from Taiwan, but beyond the Philippines the primary mechanism for the spread of both was acculturation. In fact, a slightly earlier Neolithic dispersal from MSEA, involving paddle-impressed ceramics and possibly accompanied by Austroasiatic languages, had a substantially greater genetic impact on much of ISEA, especially in the south.

## Methods

### mtDNA founder analysis

Founder analysis (Richards et al. 2000) works by identifying founder types (the result of individual migration events from source to sink in the past) and then partitioning clusters derived from them in the sink population on the basis of their coalescence times, to estimate arrival times. This mtDNA founder analysis departed from reduced-median networks (Bandelt et al. 1995) of data from HVS-I (the first hypervariable segment) of the mtDNA control region (between nucleotide positions 16,051 and 16,400), usually augmented by haplogroup-diagnostic coding-region variants.

For our sink region, we analysed 2216 mtDNA sequences from Island Southeast Asia (556 from the Philippines, 340 from Borneo, and 1320 from the rest of Indonesia), including 320 new sequences from ISEA (183 from Sabah, Brunei and Kalimantan in Borneo) and published data (Table S1). For the source we included 6070 Chinese sequences, 1429 from MSEA, 827 aboriginal Taiwanese and 4573 sequences throughout North and Central Asia. We included additional data from Malaysia (519 Malays and 308 *Orang Asli*), 55 unpublished sequences from Singapore and published data from other regions to further resolve the phylogenetic networks (Table S2). A second database was created with the aim of performing a founder analysis for Remote Oceania. Adding to the sequences above, datasets for New Guinea (846), the Karkar Islands (47), the Solomon Islands (258), the Bismarck Archipelago (1005) and Bougainville (255) were included in the new source population. Sequences from Vanuatu (130) and throughout Polynesia (148) were included in the sink population (Table S3).

We analysed the data phylogenetically haplogroup by haplogroup, and carried out the founder analysis as before (Rito et al. 2013), including a 200-year scan as a preliminary step. We estimated errors using the approach of Saillard et al. (2000), to allow for non-star-like founder clusters. In this approach, we replaced the number of samples in the ρ estimation by an effective number of samples based on the number of samples that would be present in a completely star-like network associated with the same level of uncertainty as we have implemented before (Soares et al. 2012) including at the whole-population level for South African populations (Rito et al. 2013). We employed a mutation rate of one mutation every 16,677 years for the range 16,051–16,400 (Soares et al. 2009) in the founder analysis.

We regard the scan as a heuristic approach to detecting and dating peaks of immigration, and the partition analysis as an attempt to quantify and place confidence limits upon them. Following examination of the heuristic scan, we then used archaeological evidence to finalize the dates chosen for the partition analysis, which should compensate for any systematic bias in the HVS-I mutation rate we adopted. 4.5 ka approximates the earliest likely arrival of both the putative “out-of-Taiwan” Neolithic and the paddle-impressed-ware Neolithic from MSEA in ISEA and is likely to be conservative in the case of the former—an age of 4.5 ka is more persuasive for the influence of MSEA than Taiwan, which more likely postdates 4 ka (Anderson 2005; Spriggs 2007). The putative episode of sea-level rise at ~8 ka (Pelejero et al. 1999) was initially the least well-established of the three episodes (Blanchon and Shaw 1995), but a rapid rise between 7.4 and 6.5 ka has recently received clear support (Bird et al. 2010). The existence of human dispersals at around this time is, however, supported by whole-mtDNA genome evidence from haplogroup E in ISEA and Taiwan (Soares et al. 2008), and by the founder analysis scan. For the primary settlement, we used 50 ka as a rough approximation; evidence from Niah Cave and New Guinea suggest an age upwards of 49 ka (Hunt et al. 2007; Summerhayes et al. 2010).

### Y-chromosome founder analysis

We enhanced the resolution of previously available data (Capelli et al. 2001) by augmenting with 10 more Y-STR (short tandem repeat) markers and five more SNPs. Adding to the ten previous Y-chromosome STRs (DYS388, DYS393, DYS392, DYS19, DYS390, DYS391, DYS425, DYS426, DYS389I and DYS389II), we included an additional ten: DYS460, DYS461, DYS438, DYS448, DYS458, DYS437, DYS439, H4, A10 and DYS635. We typed the ten Y-STRs in a multiplex that also included the previously typed Y-STRs, DYS388 and DYS425 (Capelli et al. 2001), to control against mixing of samples across the studies, and as a result we excluded several samples. The DYS426 marker gave inconsistent typing results across the dataset and we therefore excluded this locus from further analyses. Table S4 shows a list of the primers used in the multiplex. We ran the samples on an ABI 16-capillary 3130XL DNA Analyser at the University of Leeds.

Five SNPs were added that included: M230, which allowed the identification of the Pacific haplogroup S; M324, which allowed the identification of O3a against O3* in the previous dataset (Capelli et al. 2001); M50, which allowed the identification of subclade O1a2 within O1a, which were to prove highly relevant to identifying “out-of-Taiwan” founders; and M38 and M208, which permit the identification of subclades C-M38 and the Pacific C-M208 in samples previously labelled C* haplogroup only (Capelli et al. 2001). We typed M230, M324 and M208 using restriction analysis (Table S5 indicate primers and enzymes used), and M50 and M38 using denaturing high-performance liquid chromatography (dHPLC), as described previously (Underhill et al. 2000). We ran the samples on an automated 3500HT Wave (Transgenomic) dHPLC instrument and analyzed the results with the Navigator software. Fig. S1 summarises the SNPs analysed and the distribution of the clades identified across the sampling locations.

We used the SNP data to test the reliability of the STR analyses by constructing a median-joining network (Bandelt et al. 1999) of the overall Y-STR data. Initially we calculated a highly reticulated network using the same standard weight (10) for all STRs. We registered the number of occurrences of each STR change, and used these values to generate a graduated weighting scheme for a new network reconstruction (weight of 1 for the STR showing the highest number of occurrences and a weight of 10 for the lowest). We repeated this process until the phylogeny became stable with further iterations. The final weighting scheme gave DYS458 a weight of 1, while DYS388, DYS425, DYS437 and DYS438 were each given a weight of 10. The new STR network showed a remarkable congruence with the clustering of SNP-defined lineages (Fig. S2), with just a few minor exceptions (most particularly, ten C-M38 lineages that separated from the main cluster), showing that, although the SNP tree is limited, the Y-STR data, suitably analysed, can provide an unbiased and reliable reconstruction of ancestry. STR markers are, of course, far more unstable than bi-allelic variants but, if we consider the difficulties associated with generating a reliable tree of mtDNA haplogroups based on HVS-I data alone, we can assume we have a comparable resolution to the maternal founder analysis counterpart—possibly higher.

Zhivotovsky et al. (2004) calculated an effective mutation rate of 6.9 × 10^−4^ mutations in 25 years per Y-STR, or an average Y-STR mutation rate of 2.76 × 10^−5^ mutations per year, which is slower than estimated Y-STR pedigree rates by an order of magnitude (Gusmão et al. 2005). Since our dataset included a star-like clade that is confined to Remote Oceania (Fig. S3), and considering that the colonization time of this region is well established by radiocarbon evidence, we opted for re-calibrating the specific 19 Y-STR average mutation rate using the settlement of the Remote Pacific as the calibration point. To this end, we constructed a network of haplogroup C-M208 and applied weights obtained previously in the general Y-STR network (Fig. S4) to the network. C-M208 shows three main branches, two present in Madang and Vanuatu and a third present only in Remote Oceania (excluding Vanuatu) from Fiji to French Polynesia. The expansion into Remote Oceania began as early as ~3.3 ka, indicated by radiocarbon estimates at a Lapita site in Vanuatu (Bedford et al. 2006), while Western Polynesia (Tonga and Samoa) was colonised ~2.9 ka (Rieth and Hunt 2008). The star-like subclade, C-M208, was not present in Vanuatu and we therefore assumed an estimate of 3000 years for the age of this subclade, whose westernmost location was Fiji [see discussion in Clark and Anderson (2009)]. We estimated an average Y-STR mutation rate of 4.08 × 10^−5^ mutations per year, meaning nearly 50 % faster than the one calculated by Zhivotovsky et al. (2004). We must emphasize that this average mutation rate corresponds specifically to this group of STRs, and also that we are dealing with a very recent human event in the calibration. Our estimated mutation rate is still substantially slower when compared with father–son transmission studies (Ballantyne et al. 2010; Gusmão et al. 2005).

As for the HVS-I mtDNA data, we calculated networks for each clade. We rooted the networks by estimating the root through midpoint rooting and using an outgroup of the consensus STR length of the closest available clade to further pinpoint a hypothetical root. Again we selected founders using both *f1* and *f2* criteria (Richards et al. 2000). We used the Bayesian migration partition tool (Richards et al. 2000) in the same two ways: a scan of equally distant intervals of 200 years (Rito et al. 2013; Soares et al. 2012) and a model of migration with migration time windows based on the scan and archaeological and climatological data. This is the first direct application of founder analysis to Y-chromosome data.

The partition model included, as for mtDNA, migrations at 0.5 ka (for recent gene flow), 4.5 ka (Neolithic) and 8 ka (postglacial migrations) differing only in the time of the older migration (20 ka instead of 50 ka). We do not expect the ρ statistic (Forster et al. 1996) to be a good estimator of age for more ancient lineages in a highly mutating system such as STRs. Because of this, using a realistic time of first settlement based on archaeology of ~50 ka (Hunt et al. 2007) in the partition model would cause the older lineages dating to about 20 ka to be statistically allocated in the postglacial migration at 8 ka, to which they are closer, which we believe would be quite misleading. We therefore opted for including a migration at the time of the peak, 20 ka, even though the age of the peak probably does not correspond to the time of migration.

### Validation: founder analyses for Remote Oceania

To evaluate the performance of the methodology, we also executed a founder analysis from ISEA/Near Oceania into Remote Oceania. The well-characterized time of expansion into the Remote Pacific islands provides a valuable framework for testing the clock and the founder analysis methodology that we employed. The expansion into Remote Oceania may have begun before 3 ka into Vanuatu (Bedford et al. 2006), but the major migration into Western Polynesia began only within the last 3 ka (Rieth and Hunt 2008). In our founder analysis using a 200-year scan we obtained the same pattern for the two criteria: a single peak at 3000 years (Fig. S5), fitting very well the archaeological data.

We obtained a similar pattern for the Y-chromosome analysis (Fig. S5). However, contrary to the mtDNA analysis, we should re-emphasize that this is very far from a completely independent check for the methodology, since the rate of the Y-STRs we employed was calibrated assuming 3000 years for the major founder clade entering Remote Oceania. It is nevertheless reassuring that the time of the peak was not affected by the inclusion of the other clades in the analysis. We should note also that the estimated mutation rate is to be employed in determining migratory fractions in ISEA and not the Pacific, so its use does not provide circular evidence, only the cross-checking.

### Genome-wide analysis

We used 1251 samples taken from the Pan-Asian SNP Genotyping Database (Abdulla et al. 2009; Ngamphiw et al. 2011) (Table S6). We used the West African Yoruba data as an outgroup, as well as a South Asian group. The objective of the analysis is to compare the genome-wide patterns with the haploid marker results.

The initial dataset contained 54,794 SNPs. We pruned this dataset for linkage disequilibrium (LD) using PLINK (Purcell et al. 2007). One SNP in pairs with LD higher than *r*^2^ = 0.1 was removed in windows of 50 SNPs shifted five SNPs each time, as used before (Pierron et al. 2014; Verdu et al. 2014). We used a total of 23,332 SNPs in further analyses. We employed ADMIXTURE (Alexander et al. 2009) to estimate population structure using a maximum likelihood approach, assuming different numbers (2–15) of ancestral populations or genetic components (*K*). A few populations, namely the "Negrito" groups in Malaysia, displayed a single private component for very low values of *K* that is not present elsewhere, most probably due to the effect of strong genetic drift. As the objective of the analysis was to display overall geographic patterns to provide a frame of comparison with the uniparental markers’ phylogeography, and since these populations did not provide any relevant information in this regard, we opted for excluding them from the final analysis. We performed a cross-validation by inspecting the cross-validation error (CVE) in the analyses with different values of *K* (2–15). Theoretically, the one with the lowest CVE should be the most accurate. A graphic of the variation of the CVE obtained is shown in Fig. S6. Although the CVE does not vary much above *K* = 5, *K* = 10 displays the lowest value.

To visualize the distribution of specific mtDNA clades or autosomal components, we displayed the frequency distributions using the Kriging algorithm of Surfer 8. Data points used for the mtDNA and the genome-wide components are shown in Fig. S7.

### Whole-mitochondrial genomes

We analysed lineages from across the range of variation in mtDNA haplogroup M7, but with a particular focus on the candidate “out-of-Taiwan” marker, M7c3c. We generated a total of 114 new M7 sequences, including 38 Taiwanese, 20 Vietnamese, 16 Indonesians, 12 Peninsular Malaysians, 7 East Malaysians, 6 Laotians, 4 Chinese, 4 Micronesians (from Nauru and Kiribati), 3 Bruneians, 2 Filipinos, 1 Burmese and 1 Thai. We also extracted 51 new M7 sequences from the raw data of the 1000 Genomes project (unavailable at the start of this study). We performed whole-mtDNA sequencing as previously described (Torroni et al. 2001) using an ABI 48-capillary 3730 DNA Analyser (Taipei) an ABI 16-capillary 3130XL DNA Analyser (Leeds) and an ABI 16-capillary 3100 DNA Analyser (Porto). Details on the new and published sequences used in the phylogenetic reconstruction of haplogroup M7 are indicated in Table S7. We deposited the new whole-mtDNA sequences in GenBank (accession numbers JX987440-JX987470 and KU131308-KU131390). For comparison with mtDNA haplogroup M7, we also re-analysed haplogroups B4a1 and E using all the available published whole-mtDNA genome sequences. We list the samples we used in the analyses in Tables S8 and S9.

We reconstructed phylogenies of haplogroups M7, B4a1a and E using Network 4.6 software with the reduced-median algorithm (Bandelt et al. 1995), resolving reticulations on the basis of the relative rates of the mutations involved (Soares et al. 2009). We estimated ages for the different phylogenies using both the ρ statistic (Forster et al. 1996) and maximum likelihood (ML), using the mtDNA clock of Soares and collaborators that corrects for purifying selection from the long-term phylogenetic rate of one mutation every 3624 years (Soares et al. 2009). We estimated branch lengths in ML using PAML 3.13 (Yang 1997) assuming the HKY85 substitution model with gamma-distributed rates (32 categories). We also employed a synonymous mutation rate of one substitution every 7884 years (Soares et al. 2009) using the ρ statistic (Forster et al. 1996). We have discussed in some detail previously the potential impact of mutation rate uncertainty on phylogeographic conclusions (Mellars et al. 2013) and we re-calculated the confidence intervals in similar fashion here.

We obtained Bayesian skyline plots (BSPs) (Drummond et al. 2005) using BEAST 1.4.6. (Drummond and Rambaut 2007) to detect signatures of population increment associated with the haplogroups under analysis. We employed a relaxed molecular clock (lognormal in distribution across branches and uncorrelated between them), a mutation rate of 2.514 × 10^−8^ mutations per site per year for the whole-mtDNA genome (Pereira et al. 2010) and the HKY model of nucleotide substitutions with gamma-distributed rates, assuming a generation time of 25 years. We compared different signatures of population growth in ISEA and aboriginal Taiwanese data for the three haplogroups analysed, M7c3c, B4a1a and E.

Given a recent age estimate for haplogroup E based on a single ancient DNA sequence (Ko et al. 2014) that diverges significantly from previous estimates for this haplogroup, we performed a new estimate that was also based on ancient DNA sequences. Ancient DNA calibration is becoming a widely used approach (Ho et al. 2007), but estimates based on a single sequence are highly divergent and present large confidence intervals (Fu et al. 2013b). Calibrating a clock with only a single ancient DNA sequence can be misleading, particularly for recent samples for which even the stochastic presence of one more or one less mutation than the expected clade average can lead to strong departures from a realistic mutation rate. For calibration purposes, we added two more ancient East Asian sequences to the haplogroup E sequence described by Ko et al. (2014). One dates to the same time-frame as the E sequence: Boshan 11, from north-east China, at 8.18 ka (Fu et al. 2013b). The second one is older: Tianyuan 1301, also from north-east China, at 39.5 ka (Fu et al. 2013a); it is important to include this in the analysis, since haplogroup E as a whole will necessarily be older than the ancient haplogroup E sequence. In the analysis, we used a tree that represents a snapshot of human diversity (Table S10), which allows a better estimate of the evolutionary parameters in mtDNA, with all the main haplogroup E branches represented. We employed a relaxed molecular clock (lognormal in distribution across branches and uncorrelated between them) using BEAST 1.4.6. (Drummond and Rambaut 2007), with a constant population size and the HKY model of nucleotide substitutions, with gamma-distributed rates.

## Results

### mtDNA control-region and Y-chromosome founder analyses

To investigate the genetic input into ISEA through time, we carried out founder analyses with both mitochondrial DNA (mtDNA) control-region sequences—for the maternal line of descent—and Y-chromosome variation using a 19 Y-STR dataset within SNP-defined lineages—for the paternal line of descent. Founder analysis is a quantitative phylogeographic approach developed to evaluate the diversity of lineage clusters that has arisen within a particular geographic sink region (in this case, ISEA), following migration from a specified (assumed) source region (in this case, MSEA/China/Taiwan). Using the molecular clock to convert to time depth, these values are a proxy for the minimum arrival age of each founder cluster in the sink (Richards et al. 2000).

**Fig. 1:**
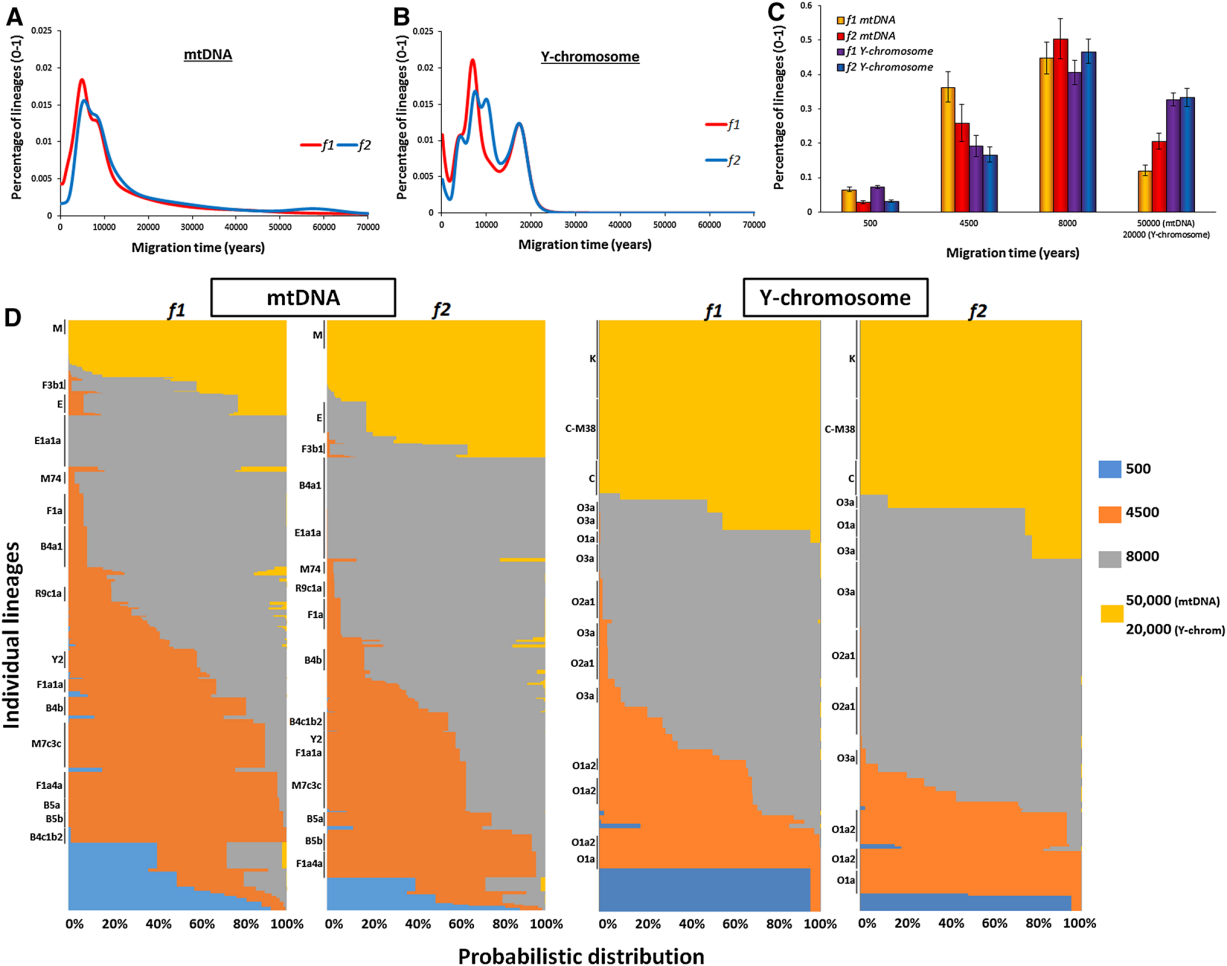
Founder analysis results for ISEA, assuming Taiwan as source, for mtDNA (female lineages) and Y-chromosome variation (male lineages). **a** Probabilistic distribution of mtDNA founder clusters across migration times scanned at 200-year intervals from 0 to 70 ka, using two criteria for founder identification, *f1* and *f2*; **b** probabilistic distribution of Y-chromosome founder clusters across migration times scanned at 200-year intervals from 0 to 70 ka, using two criteria for founder identification, *f1* and *f2*; **c** proportion of founder lineages in a four-migration model for mtDNA and Y-chromosome variation using two criteria for founder identification, *f1* and *f2*; **d** probabilistic distribution of each individual lineage in mtDNA and Y-chromosome variation in a four-migration model chromosome using two criteria for founder identification, *f1* and *f2*. Individual founder clusters with more than 2 % frequency in overall ISEA (sink populations) are indicated at the left-hand side of each *plot*

For maternal lineages, the 200-year scan of founder lineages dispersing into ISEA (Fig. 1a) identified two major coalescence peaks (corresponding to bursts of immigration) under the two criteria we employed, *f1* and *f2* (Fig. 1a) (Table S11), at 4.6–4.8 ka and at 8–10 ka, respectively. We also observed a slight hump ~55 ka with the *f2* criterion alone.

For Y-chromosome variation (Fig. 1b), we obtained very similar peaks with both criteria (one at 4–5 ka and a second at ~8 ka). Rather remarkably, the two main peaks in two different genetic systems with distinct mutation rates and estimated using two distinct founder criteria are consistent across each of these different analyses. In addition, we observed an increment representing very recent migrations and, with the *f2* criterion, a further extra peak at 10–11 ka. This peak might signal the second well-defined episodic flood immediately after the Younger Dryas (Pelejero et al. 1999). We did not include it in the migration model, however, for two reasons: it was detected only under a single criterion and with one genetic system; and, in any case, founders at this peak will be included statistically in the ~8 ka migration that overall can be defined as postglacial migrations. The oldest arrivals here date to ~20 ka, largely haplogroup K and C lineages. This may well correspond with the ancient minor peak for mtDNA; we expect ρ dating with STRs to provide severe underestimates for ancient clades because of mutational saturation. However, for the present analysis this is a minor issue, since we are concerned primarily with events in the Holocene. Particularly in the case of K, an older age than this could be expected, considering that K probably evolved in the region since the first settlement as displayed by the high prevalence of K* and K subclades in the ancient Sahul populations, including Aboriginal Australians (Hudjashov et al. 2007).

We then partitioned the founders in ISEA using a migration model informed not only by the scan results in the two genetic systems, but also archaeological and palaeoclimatological evidence, to quantify the contribution of each immigration event to the extant mtDNA and Y-chromosome gene pools in ISEA. The model from mtDNA data here assumes migrations at 4.5, 8 and 50 ka, corresponding to Neolithic immigration, postglacial expansions and first Pleistocene settlement. We assumed a further dispersal at 0.5 ka to allow for any recent/historical gene flow.

For Y-chromosome variation, we used a more recent age of 20 ka to cover the more ancient migrations, as mentioned above. However, the matching of peaks at 4–5 ka and 8–10 ka for both the paternal and maternal line of descent is striking. The overall contribution at each proposed migration time for each of the two founder criteria in the mtDNA and Y-chromosome variation is shown graphically in Fig. 1c, d. The mtDNAs coalescing at the time of the first settlement (~50 ka) accounted for ~10 to 20 % of modern mtDNA lineages in ISEA. Note that many lineages from the ancient Sunda continent would very likely be present across both ISEA and MSEA, which were only finally separated by sea-level rise ~8 ka. However, MSEA is a source region in this analysis, so this value in the founder analysis corresponds to ancient lineages private to ISEA only. In the mtDNA analysis, lineages descending directly from the haplogroups carried by the first settlers correspond to M*, N*, R* and possibly haplogroup F3 (Fig. 1d). Although a recently published ancient mtDNA haplogroup E sequence (Ko et al. 2014) was used to suggest a Taiwanese source for this clade, an early origin in ISEA (Soares et al. 2008) remains more likely, as discussed below. At this ancient time-frame, Y-chromosome lineages (with STR ρ dating) are uninformative due to saturation, but haplogroups K* and even C may date to the first colonization at that time. These are above 30 % in the Y-chromosome analysis.

Overall, the migration at ~8 ka contributes the most lineages to the current gene pool of ISEA with a fraction of ~40–50 % in both mtDNA and Y-chromosome variation (Fig. 1c). We stress again that, statistically, this migration time could include lineages entering ISEA throughout the period of sea-level rises, from 14 to 8 ka, covering all three flooding episodes (Pelejero et al. 1999). This partition probabilistically includes major and well-studied haplogroups such as B4a1a (Soares et al. 2011), subclades of haplogroup E (Soares et al. 2008), F1a*, and subclades of haplogroup M shared between ISEA and MSEA, with B4a1a and E the major contributors. In Y-chromosome variation, this migration includes most clusters within haplogroups O2a1 and O3 and a subclade of O1a (Fig. S4), matching to some extent the results of Karafet et al. (2010) indicating that O1a* entered ISEA before the Neolithic. We should note that in our recent Y-chromosome survey (Trejaut et al. 2014), O2 and O3 clades declined in frequency moving north from ISEA towards Taiwan, the opposite of what one might expect from an “out-of-Taiwan” movement. A previous survey (Karafet et al. 2010) also suggested that O3, O2a1 and O1a* entered ISEA from the mainland before the Neolithic period.

The contribution at the time of the Neolithic, at 4–5 ka, varied with the criterion and the genetic system, but 25–35 % is probably the best estimate (Fig. 1c). (The *f1* criterion in mtDNA probably overestimates recent migration due to the large size of the source sample used.) Only one major founder presented significant differences between the analyses: B4b appears Neolithic in *f1* criterion and part of the postglacial migration in the *f2* criterion (Fig. 1d). This haplogroup deserves further attention in the future. The widely held model for the spread of the Neolithic in ISEA implicates expanding pre-Austronesian/Austronesian speakers from South China/Taiwan (Bellwood 1997); but in fact not all of the Neolithic founders we identify support this hypothetical “out-of-Taiwan” dispersal. A large fraction of Neolithic mtDNA founder clusters from haplogroups B5a1 and F1a1a (~10 % out of the 25–35 % Neolithic lineages in the analysis) appear to have originated in MSEA, and are rare or absent in either Taiwan or the Philippines.

**Fig. 2:**
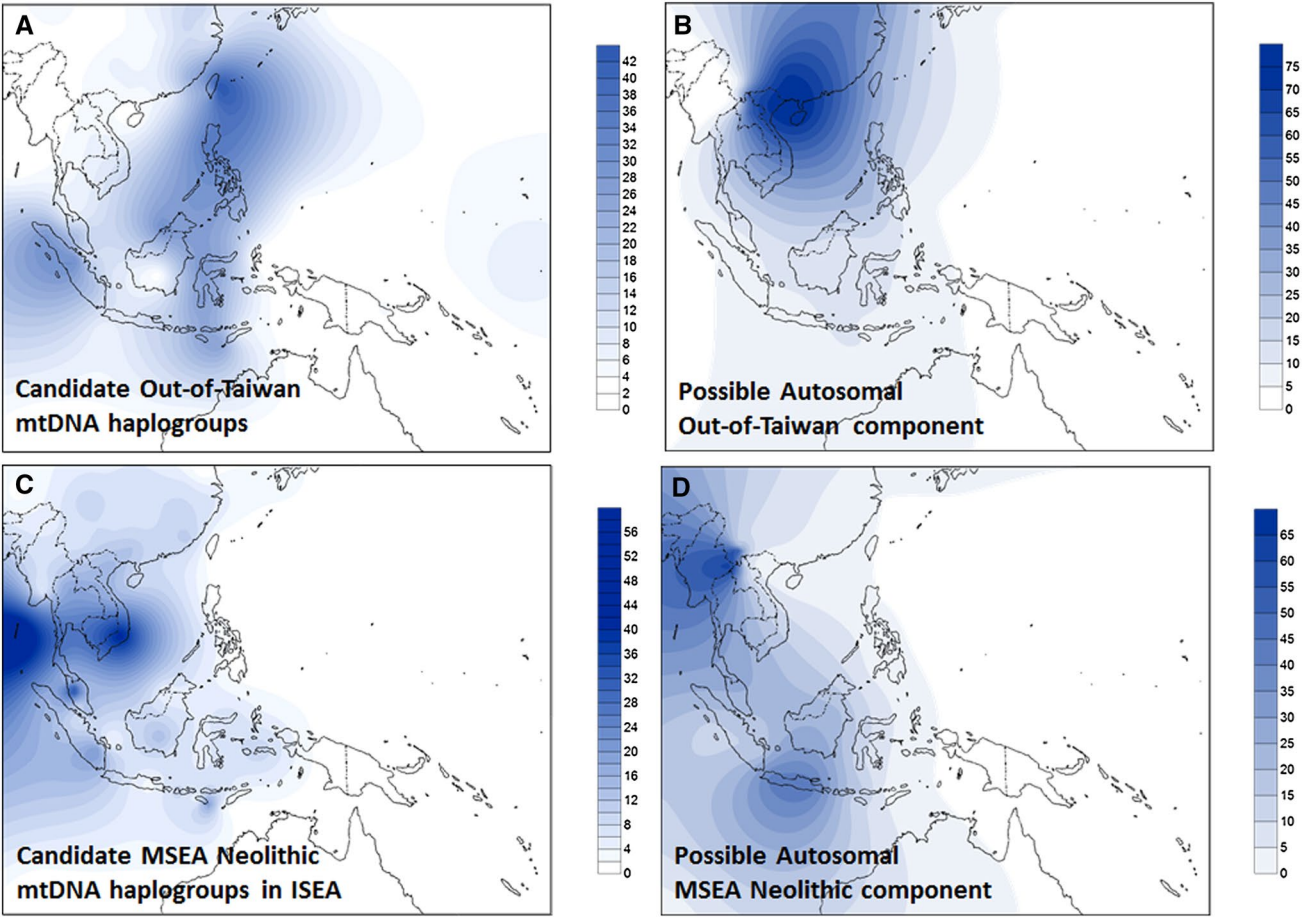
Frequency map of probable Neolithic markers (lineages argued to track one or other of the dispersals associated with Neolithic ceramics) in mtDNA and genome-wide data. **a** Pooled frequency of candidate “out-of-Taiwan”, “Neolithic II” mtDNA haplogroups, based on founder analysis. **b** Possible “out-of-Taiwan”, “Neolithic II” component in the genome-wide data when considering 10 ancestral populations in the ADMIXTURE analysis. **c** Pooled frequency of candidate MSEA “Neolithic I” haplogroups in ISEA. **d** Possible MSEA “Neolithic I” component in the genome-wide data when considering 10 ancestral populations in the ADMIXTURE analysis. The outline map was obtained from http://www.outline-world-map.com

Our results therefore suggest that mid-Holocene Neolithic immigration into ISEA was in part via MSEA, temporally associated with spread of basket-marked and carved paddle-impressed pottery, which appeared across MSEA as early as red-slipped pottery appeared in Taiwan (Bulbeck 2011), and possibly involving speakers of Austroasiatic languages (i.e. Anderson’s “Neolithic I”) (Anderson 2005). The mtDNA haplogroups M7c3c, Y2, F1a4a, B4c1c and possibly B4b (which shows contrasting patterns under the two criteria) may, however, represent genuine “out-of-Taiwan” clades in ISEA. These founders are all derived from Chinese-mainland source haplogroups, and within Austronesian-speaking populations they have a higher overall frequency in Taiwan and the Philippines (Fig. 2a). This input, at ~20 %, lends support to a modified, small-scale “out-of-Taiwan” model [Anderson’s “Neolithic II” (Anderson 2005; Donohue and Denham 2010)], proposed to explain the appearance of red-slipped pottery in relation to the early dispersal of Austronesian languages.

On the male line of descent, the Neolithic contribution is lower (15–20 %) but, since MSEA is not represented in the Y-chromosome dataset, all these Neolithic founders are likely to represent the putative “out-of-Taiwan” dispersal, mirroring closely the ~20 % “out-of-Taiwan” founders for mtDNA. Most of O1a and all of O1a2 likely represent signals of Neolithic migrants from Taiwan, confirming earlier suggestions (Karafet et al. 2010; Trejaut et al. 2014). A portion of O3a (~10 % in the *f1* criterion) was also partitioned into the Neolithic in our analysis.

### Corroboration of founder analyses with genome-wide evidence

We next compared these results with patterns observed in autosomes, using genome-wide data from the Pan-Asian SNP Genotyping Database (Abdulla et al. 2009; Ngamphiw et al. 2011) and the ADMIXTURE software.

**Fig. 3:**
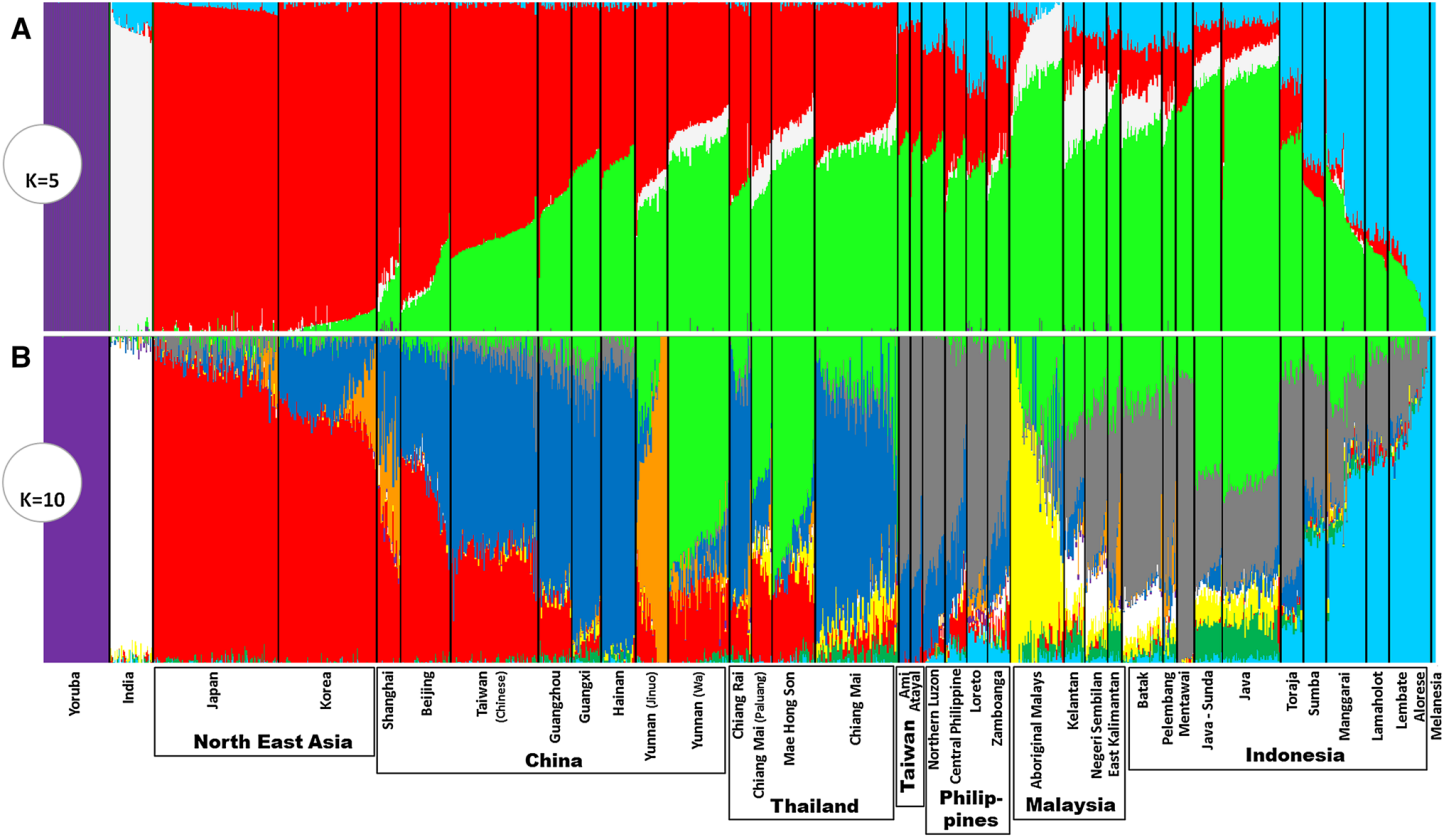
Reconstruction of ancestry in Asian populations using ADMIXTURE. Considering **a** five ancestral populations (*K* = 5) and **b** 10 ancestral populations (*K* = 10)

At a more basal level, the first that seems anthropologically and genetically potentially valid (*K* = 5, which includes African, South Asian and Near Oceanian components in purple, white and blue) (Fig. 3a), the East Asian autosomal data separate into a Southeast Asian component (green) with a focus on the ancient Sunda continental shelf (MSEA, Sumatra, Java and Borneo) that varies from ~80 % around Borneo and drops in frequency as one moves north, and a Chinese/Northeast Asian component (red), which varies between 100 and 60 % in mainland China. Frequencies of the latter in Taiwan (~30 %) and Southeast Asia (5–30 %) match the mtDNA picture of Neolithic-age Chinese gene flow into ISEA (Fig. S8; cf. Fig. 2a). It is, however, difficult to directly connect a given component in ancestry analysis with a given demographic occurrence. One could calculate the time of admixture, but admixture ages are not necessarily indicative of time of migration (Lipson et al. 2014). In addition, the ages calculated are sometimes dubious and under-estimated as the estimated time of split between Europeans and New Guineans suggests (Wollstein et al. 2010).

Analyses from *K* = 6 to *K* = 15 generate additional components by further sub-dividing these Northeast and Southeast Asian components, whilst maintaining the African, South Asian and Melanesian/Near Oceanian components intact across the analyses. The autosomal estimate with ten ancestral populations, theoretically the best estimate of ancestry for the data as it has the lowest cross-validation error, includes seven components with discernible frequencies in at least one location in Austronesian-speaking populations (Fig. S9). The South Asian component (white) is present at low frequencies only in the Malay Peninsula and Sumatra, matching the historical record (Manguin et al. 2011). The Northeast Asian component (red) is seen at appreciable frequency only in the Philippines (at only very low rates). A Near Oceanian component (pale blue) dominates many of the populations of Eastern Indonesia, as expected. Two minor components (dark green and yellow) are virtually specific to ISEA, mainly in what was western Sundaland (Java/Sumatra/Malay Peninsula), with one (yellow) markedly elevated in Aboriginal Malays.

One important component (grey) is both specific for Austronesian-speaking populations and highly frequent across ISEA (Fig. S9). It reaches 60–70 % in the two aboriginal Taiwanese groups in the sample—the equivalent cluster in Pan-Asian SNP data approaches 100 % (Abdulla et al. 2009)—peaking in our dataset in the Philippines, Sumatra and Sulawesi (70–90 %), and is virtually absent from Continental Asia, suggesting an insular origin. Comparison between the analyses with five and ten ancestral populations also suggests that this was part of the larger Southeast Asian component in the former. Considering the major postglacial signal observed in mtDNA and Y-chromosome variation in both our founder analysis and in earlier analyses (Hill et al. 2007; Karafet et al. 2010; Soares et al. 2008, 2011; Trejaut et al. 2014), and the sharing of many lineages between ISEA and Taiwan (Soares et al. 2008, 2011), this autosomal component may correspond to an ancestral cluster common to both Taiwan and ISEA that was established before the hypothetical dispersal of Austronesian. Even if we consider that there is likely a signal of Austronesian expansion “out-of-Taiwan” in the genome-wide data (see below), this component, which is most frequent in Taiwan, the Philippines, the Mentawai Islands and Sulawesi, disparate islands at opposite extremes of the Sunda shelf, could explain why a maximum likelihood population tree of the Pan-Asian SNP data indicated Taiwan as an offshoot of ISEA diversity (Abdulla et al. 2009). Such population trees only depict broad patterns and, although a minor component could show an ancestry in Taiwan when compared with ISEA, the most frequent component could show the overall opposite ancestry.

Two autosomal components that might signal Neolithic dispersals can be compared with the patterns obtained from Neolithic founder candidates in the mtDNA analysis. One of these components (paler green in Fig. 3b) is frequent in MSEA/Southwest China (up to ~70 %) and varies from 5 to 40 % in Indonesia (Fig. 2d), but is absent from Taiwan and rare in the Philippines. It is probably a relatively recent arrival as it is not evenly distributed across ISEA. The MSEA Neolithic candidates in the mtDNA also show a strong peak of frequency in MSEA and frequencies of 5–30 % in Indonesia, but are rare in the Philippines and Taiwan (Fig. 2c). We can also match these distributions with the presence of basket-marked and carved paddle-impressed pottery: in Sarawak, assemblages at ~4.5 ka with carved cord-wrapped or basketry-wrapped paddle-impressed pottery (Bellwood 1997; Bulbeck 2008) show the influence of an early Neolithic from MSEA in Western Indonesia.

The final component (dark blue in Fig. 3b) has a high frequency in South China (Fig. 2b) and is also seen in Taiwan at ~25–30 %, in the Philippines at ~20–30 % (except in one location which is almost zero) and across Indonesia/Malaysia at 1–10 %, declining overall from Taiwan within Austronesian-speaking populations. The mtDNA candidates for “out-of-Taiwan” markers (Fig. 2a) also show an overall frequency of up to ~35 % in Taiwan and the Philippines, but are almost absent in parts of Borneo, Java and Eastern Indonesia. Sumatra superficially presents a more discordant picture between genome-wide and mtDNA results, but the sampling of the Pan-Asian SNP dataset involves only Batak people whilst our mtDNA sampling involved the wider Sumatran population. We should also bear in mind that the genome-wide sampling lacks major areas of ISEA, including the whole of Borneo.

Therefore, the overall picture from the ADMIXTURE analysis with 10 ancestral populations where the cross-validation error was the lowest, is concordant with the mtDNA and Y-chromosome pattern, with a minor Neolithic input from MSEA, probably immediately preceding a Neolithic input from Taiwan (Anderson 2005) that had a strong demographic impact in the Philippines, but a much more minor genetic input elsewhere in the Indo-Malaysian Archipelago.

### Confirmation with whole-mtDNA genome data

Although providing much larger sample sizes, the low phylogenetic resolution of mtDNA HVS-I data can create problems for phylogeographic analyses such as founder analysis, for example by conflating distinct founders. In parallel, we therefore checked the phylogeographic signal with the much better resolved whole-mtDNA genomes for the major “out-of-Taiwan” haplogroup in the founder analysis, M7c3c. In particular, we wished to compare the results for M7c3c with the two putative postglacial signals for haplogroups E and B4a1a (Soares et al. 2008, 2011).

**Fig. 4:**
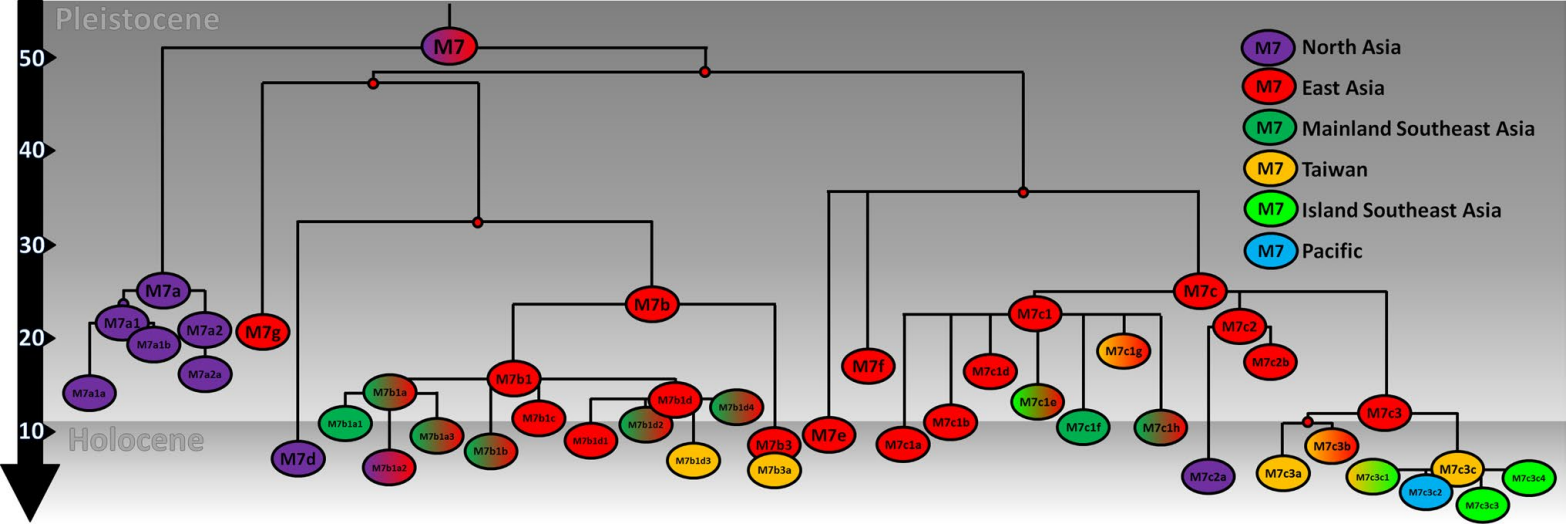
Schematic tree of haplogroup M7. The tree is scaled using maximum likelihood and a time-dependent molecular clock for whole-mtDNA genomes

Haplogroup M7 dates to just over 50 ka. An overall mainland Eastern Asian distribution is clear for the M7 phylogeny (Fig. 4; full tree in Supplementary Material 2). There are two basal branches, M7a, which displays a strong Northeast Asian ancestry centred on Japan and Korea, and a second major clade encompassing M7b, M7c, M7d, M7e, M7f and M7g, which we refer to as M7b′c′d′e′f′g. This splits into two further major subclades, M7b′d′g and M7c′e′f both with an East Asian ancestry.

The overall phylogenetic and phylogeographic pattern is strikingly clear: both aboriginal Taiwanese and Island Southeast Asian-specific lineages are close to the tips of an overall mainland Eastern Asian distribution. The major subclade of M7b3, M7b3a, is only present in Taiwan and ISEA. It is frequent in Taiwan (at ~10 %) and considering its age (~6 ka) seems likely to have arrived in Taiwan with the rice Neolithic from South China; but it is vanishingly infrequent across ISEA. In M7b1, M7b1d3 is also restricted to Taiwan, and with a similar age may also have arrived from China with the Neolithic, but again it is virtually absent from ISEA.

In M7c′e′f, the three subclades branch from a single node and all show evidence of East Asian ancestry. Within M7c, M7c3 is by far the most frequent and the only one to disperse significantly into Taiwan and ISEA. This clade probably had an origin in South China, with several subclades also present in Taiwan. Its major subclade, M7c3c [M7c1c in Hill et al. (2007)], here re-dated with whole-mtDNA genomes to ~5 ka, is restricted to Austronesian-speaking populations (both Taiwan and ISEA). Given the presence of other subclades of M7c3 in Taiwan and South China, the most probable source for M7c3c is in Taiwan (amongst M7c3 arrivals from China, again perhaps with the rice Neolithic), with subsequent dispersal into ISEA. Several subclades of M7c3c exist throughout Taiwan and ISEA, and there is also one in the Pacific (M7c3c2, found in both Micronesia and the Solomon Islands), dating to less than 3 ka. This pattern confirms M7c3c as a strong candidate for an “out-of-Taiwan” marker, as indicated by the HVS-I founder analysis.

We can contrast this distinctive pattern with the distribution of haplogroups B4a1a and E, both of which are—like M7c3c—largely restricted to insular, Austronesian-speaking populations. For that reason they have been proposed as candidates for “out-of-Taiwan” markers, but neither shows a direct ancestry in South China. We propose here a set of phylogeographic parameters that we expect to see fulfilled in a clear-cut “out-of-Taiwan” marker:

- If the haplogroup was carried into Taiwan from South China by rice-agriculturists ~6 to 8 ka, the dispersal’s timing should be bracketed by the age of the ancestral clade seen in South China (upper bound) and the insular Austronesian-specific subclade (lower bound);
- the insular and Austronesian-specific subclade should date to after the arrival of rice-agriculturists from China ~5.5 ka, but before the “out-of-Taiwan” migration ~4.5 ka;
- the founder age in ISEA for the subclade should date to ~4.5 ka, the time of the “out-of-Taiwan” dispersal;
- the founder age from Taiwan/Philippines to the rest of ISEA should be lower than the date of the “out-of-Taiwan” migration, ~4 ka; and
- the expansion of the clade in Taiwan should predate the expansion in ISEA.

**Fig. 5:**
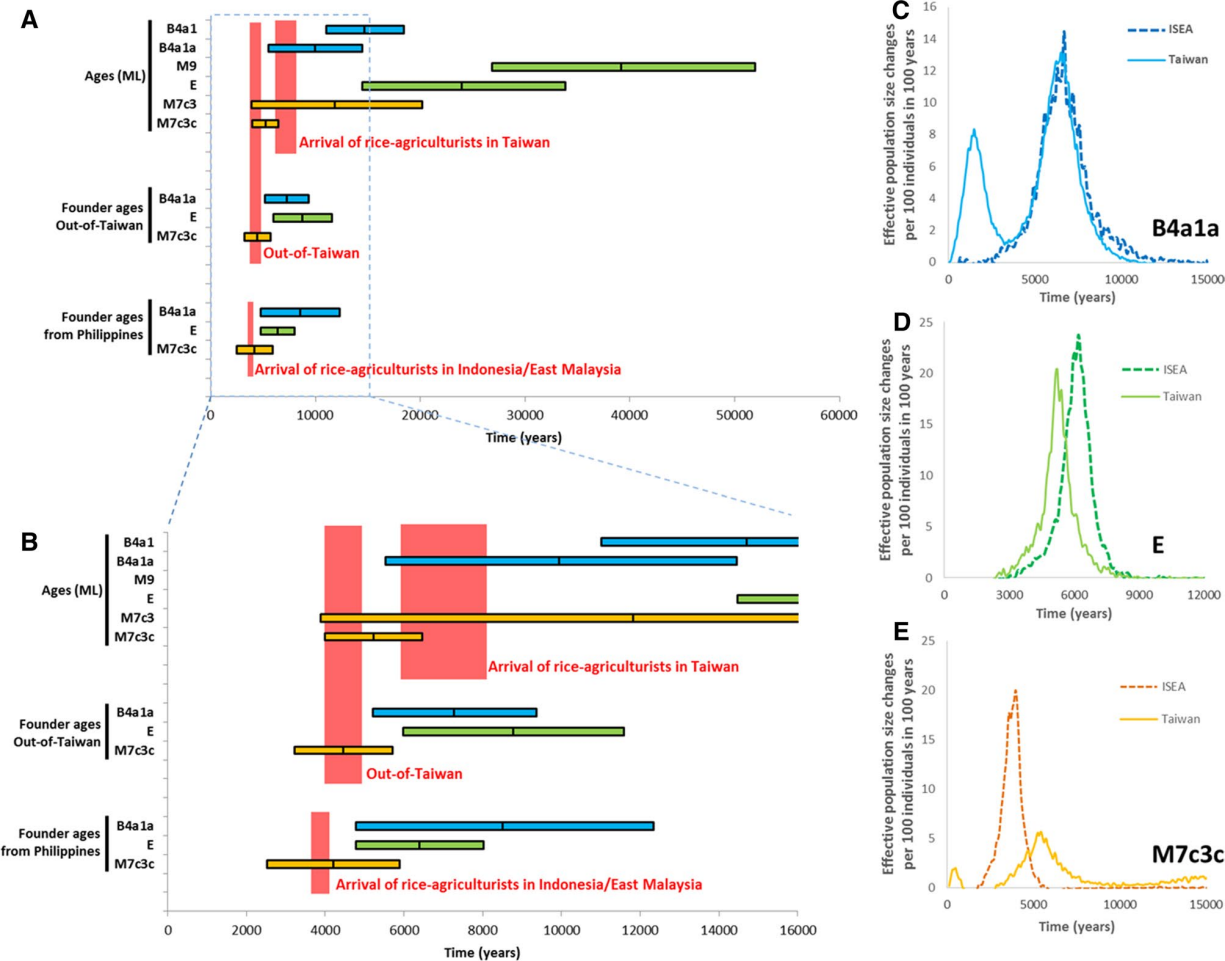
Phylogeographic patterns in haplogroups M7c3c, E and B4a1a1. **a** ML ages of key clades in the test for an “out-of-Taiwan” pattern; ρ founder ages from Taiwan into ISEA; ρ founder ages from Taiwan and the Philippines into the rest of ISEA. **b** Detailed view of the most relevant time-frame for the data in **a**. **c**–**e** Increments in expansion of haplogroups B4a1a (**c**), E (**d**) and M7c3c (**e**), measured from Bayesian skyline plots as effective population size change per 100 individuals per 100 years, in Taiwan and ISEA

We evaluated each of these points in turn (Fig. 5; Table S12; note that taking into account mutation-rate uncertainty, as documented in Table S12 does not alter the conclusions). First, we consider the ML ages of key subclades, then founder ages, and finally Bayesian skyline plot (BSP) expansion time estimates. Regarding (a), B4a1a appears in Austronesian-speaking populations between 14.7 [11.0; 18.5] ka, the age of the continental ancestral clade B4a1, and 9.9 [5.5; 14.5] ka, the age of B4a1a; haplogroup E appears between 39.2 [26.9; 52.0] ka, the age of ancestral M9, and 24.0 [14.5; 33.9] ka; and M7c3c appears between 11.8 [3.9; 20.2] ka- the age of M7c3- and 5.2 [4.0; 6.5] ka. Only M7c3c clearly fits an arrival in Taiwan in line with the “out-of-Taiwan” model. B4a1a cannot be completely ruled out from these estimates, given the 95 % confidence interval of the age estimate, but it is nevertheless very unlikely (Fig. 5a, b).

Point (b) stipulates that the insular subclade should originate after the hypothetical arrival of rice-agriculturists in Taiwan and before the dispersal “out-of-Taiwan”. M7c3c, at 5.2 [4.0; 6.5] ka, follows this pattern; B4a1a, at 9.9 [5.5; 14.5] ka, and haplogroup E, at 24.0 [14.5; 33.9] ka, both suggest an earlier origin within currently Austronesian-speaking populations.

Taking point (c), an average founder age for M7c3c from Taiwan into ISEA is 4.4 [3.2; 5.7] ka, matching the 4.5 ka prediction of the “out-of-Taiwan” model. Haplogroups E and B4a1a yield 8.8 [6.0; 11.6] ka and 7.3 [5.2; 9.4] ka, respectively, suggesting earlier postglacial expansions. When including the Philippines along with Taiwan as part of the source for the dispersal—point (d)—the founder for haplogroup M7c3c dated a little lower at 4.2 [2.5; 5.9] ka—a striking match to the hypothetical Austronesian arrival in the Indo-Malaysian archipelago. Haplogroup E, by contrast, yielded 6.4 [4.8; 8.0], and the B4a1a point estimate actually increased to 8.5 [4.8; 12.3] ka, when compared with the previous founder age estimate into ISEA as a whole, clearly indicating that the “out-of-Taiwan” assumption of the founder model in this case is likely to be false.

Finally (e), we used BSPs to estimate the expansion time of each haplogroup. Figure 5c–e show the increment or rate of expansion (corresponding skyline plots in Fig. S10; data in Table S13). The B4a1a data for Taiwan and ISEA (Fig. 5c) suggest a very similar time of expansion, starting ~10 ka (with a second expansion restricted to Taiwan ~2000 years ago). However, haplogroup E expanded in ISEA before Taiwan (Fig. 5d), starting ~8 ka for ISEA and ~7 ka for Taiwan. Finally, for M7 we see a first expansion in Taiwan starting ~7.5 ka, peaking at 5.2 ka, while for ISEA the expansion starts later at 5.2 ka with peak at ~4 ka, corresponding closely to the “out-of-Taiwan” model.

Therefore, haplogroup M7c3c meets all the criteria expected for an “out-of-Taiwan” marker, whereas haplogroups E and B4a1a meet none of them. Yet a haplogroup E lineage recently recovered from human remains in the Strait of Taiwan, dating to ~8 ka, evidently represents a sequence ancestral to the E1 subclade, leading Ko et al. (2014) to suggest an origin of haplogroup E ~10 ka ago in China or Taiwan and a Neolithic migration into ISEA (based on a Bayesian analysis). This compares with our estimate for the age of haplogroup E with the time-dependent clock (Soares et al. 2009) of ~24 ka (Fig. 5). Previous age estimates based on the time-dependent clock and Bayesian ancient DNA calibrations do not differ to this extent (Fu et al. 2013b), despite some claims to the contrary. The authors of one recent estimate based on several ancient sequences claim that their estimated rate is 45 % faster than the one we estimated (Brotherton et al. 2013), but this arises from their comparing their estimated rate with our inter-specific phylogenetic rate rather than the time-dependent rate. For the time-frame of the European Neolithic and Bronze Age with which they were concerned, our curve indicates a mutation rate of 2.307 × 10^−8^ substitutions per site per year for the time of 6.15 ka (their oldest sample), only 4 % slower than the one they estimated. The difference would be even less for the age of their other, younger samples.

Here, indeed, we estimate an age for haplogroup E of 29.7 [18.5; 43.9] ka and an average mutation rate of 2.041 × 10^−8^ [1.54 × 10^−8^; 2.48 × 10^−8^] substitutions per site per year using a Bayesian estimate with two additional East Asian ancient DNA sequences. Given that the root of haplogroup E is seven mutations from the root of the “out-of-Africa” haplogroup M (Macaulay et al. 2005; Mellars et al. 2013) which has an average branch length to the present-day (~50,000 years) of ~20 mutations, age estimates for E more recent than ~20 ka seem implausible.

Involving haplogroup E in a wide-scale Neolithic dispersal across and out of mainland China also ignores the evidence that haplogroup E is restricted to the off-shore islands and has never been seen in any extant Chinese populations. Its age of >20 ka and insular distribution rather suggest an origin on the eastern side of the Sunda shelf. Although the early Holocene haplogroup E sequence creates a deeper link within E1, extant diversity haplogroup E diversity nevertheless remains deeper in ISEA, for both E1 and E2 (Soares et al. 2008). Moreover, a large mtDNA survey of aboriginal Taiwanese groups, which probably diverged early in Austronesian history, but were subsequently isolated and experienced drift very differently from other Austronesian populations, failed to detect any novel haplogroup E diversity, finding the same sub-set of ISEA diversity (Ko et al. 2014). The 8-ka age of the sample would place it in a period of intense postglacial expansions, due to huge sea-level changes resulting from global warming, and might be better explained as an offshoot from the south, where many lineages were lost in the postglacial period. We would caution against drawing strong conclusions from a single sample. Nevertheless, regardless of its point of origin, our analyses show that haplogroup E most probably expanded in ISEA well before the Neolithic period.

## Discussion

Settlement models of ISEA that emphasize climate change and drastic shifts in the population in the early postglacial period have tended to side-step the linguistic evidence for a Taiwanese origin of the Austronesian languages (Donohue and Denham 2010). Although languages may sometimes be transmitted solely horizontally, for example by trade, this seems unlikely to explain the pattern of the Austronesian languages in ISEA as a whole, in the context of such a wide and ecologically complex region (Blench 2012). We address this issue here from the standpoint of genetic variation across the genome.

Previous results have shown a strong common ancestry between ISEA and Taiwan populations predating the pottery Neolithic period for mtDNA and Y-chromosome variation (Capelli et al. 2001; Hill et al. 2007; Karafet et al. 2010; Trejaut et al. 2014; Tumonggor et al. 2013), as well as indications that some minor lineages entered ISEA during the Neolithic. Here we show that two Neolithic waves entered ISEA, as previously suggested on the basis of pottery comparisons (Anderson 2005) and recently from autosomal analyses (Lipson et al. 2014), but that both were small-scale affairs.

The first Neolithic migration, from MSEA [“Neolithic I” in the scheme of Anderson (2005)], reflected in the distribution of haplogroups B5a1 and F1a1a and the “pale green” genome-wide component, took place ~4.5 ka and affected mainly Western Indonesia/Borneo—although it extended as far as Eastern Indonesia, particularly in the south, even reaching regions of contact with Papuan populations. A signal for this dispersal was also recently proposed by Lipson et al. (2014), although they favoured admixture with Austronesian agriculturists dispersing around the coasts of MSEA as an explanation, which our results render unlikely.

The second Neolithic wave [“out-of-Taiwan” or Anderson’s “Neolithic II” (Anderson 2005)] is marked by the appearance of red-slipped pottery ~4 ka (Spriggs 2007, 2011) and impacted strongly on the Philippines (accounting for 30–40 % of current genetic diversity), where domesticated rice does indeed appear relatively early in the archaeological record (Paz 2002). However, for the rest of ISEA (the Indo-Malaysian archipelago), the demographic impact was much lower—often negligible. The overall fractions of “out-of-Taiwan” immigrants in the founder analysis for both mtDNA and Y-chromosome variation are very similar at ~15 to 20 %, suggesting that previous models inferring highly divergent male and female contributions are incorrect (similarly to the Pacific). The mtDNA haplogroup M7c3c, in particular, closely matches the expected pattern for an “out-of-Taiwan” marker.

Thus, although the Neolithic dispersal from Taiwan suggested by red-slipped pottery proves not to have been a large-scale demographic event (at least, beyond the Philippines), it did indeed occur, and followed an expansion into Taiwan from South China, as one archaeological model predicts (Bellwood 1997). However, we must be careful what we mean by the term “Neolithic”, since the archaeological record for most of ISEA primarily indicates the appearance of various novel ceramics, and provides little or no evidence for large changes in the subsistence base. The low level of settlement across ISEA at this time accords not with large-scale demic diffusion fuelled by rice agriculture, but with more with archaeological views that stress the transition from grain cultivation to the root and arboreal crops that dominate agricultural systems in the western Pacific (Donohue and Denham 2010; Paz 2002). It is clearly parsimonious to conclude that these sea-faring settlers spoke Austronesian and spread their languages across ISEA, but they may have had rather little to do with either rice farming or arboriculture/vegeculture (aspects of which originated much earlier, in part diffusing from Near Oceania (Barker and Richards 2013; Blench 2012).

The low scale of the migrations overall concurs with recent archaeological evaluations (e.g. Spriggs 2011), but contrasts sharply with the recent interpretation of Lipson et al. (2014). However, their assumption that aboriginal Taiwanese represent the source for ISEA, their use of only three autosomal source clusters and their extremely recent age estimates for admixture times (within the last 2200 years) compromise their conclusions. Our analysis supports a scenario in which language shift played the major role, rather than large-scale population replacement (Donohue and Denham 2010, 2015).

The genetic situation further east seems to require a model where language was transmitted mostly horizontally across the north coast of New Guinea. Curiously, M7c3c (most or all probably belonging to the subclade M7c3c2 dating to ~2.6 ka) and some other putative “out-of-Taiwan” subclades (like B4b1) are detected at relatively high frequencies in Eastern Micronesia/Northwest Polynesia. These lineages may have been carried directly through Western Micronesia from the vicinity of the Philippines (Fitzpatrick and Callaghan 2013; Hung et al. 2011). This migration was, however, distinct from the primary spread of the Austronesian languages into the Pacific, and would be expected to have affected mainly the Marianas.

Otherwise, whilst languages may have moved alongside other lineages integrated within ISEA, “out-of-Taiwan” haplogroups are virtually undetected across the north coast of New Guinea, the Bismarck Archipelago or the Solomon Islands. Minor exceptions include 1.4 % M7c3c in the Admiralty Islands (Kayser et al. 2008a), <0.2 % in New Britain (Friedlaender et al. 2007) and two closely related whole-mtDNA M7c3c sequences (~2 %) in the Solomon Islands (Duggan et al. 2014). M7c3c sequences, all within M7c3c2, are also seen in Ontong Java, a Polynesian outlier in the north Solomons. M7c3c and the other probable “out-of-Taiwan” clades have not been detected in Vanuatu, Fiji or Samoa, despite very extensive sampling.

Most of the present-day diversity in Near and Remote Oceania was established in New Guinea by ~10 ka (Soares et al. 2011), a fraction of which was carried by Austronesian speakers into the Remote Pacific. Powerful, long-established spheres of interaction may have facilitated the spread of the Austronesian languages in the south (Bulbeck 2008; Terrell and Welsch 1997; Torrence and Swadling 2008). They may thus have spread stepwise from the north and west via small-scale interactions and waves of acculturation. There appears to have been no “Austronesian farming-dispersal” in any meaningful sense across ISEA—early Austronesian speakers were more likely fisher–foragers—opening up the discussion to a range of innovative archaeological and linguistic models (Barker and Richards 2013; Donohue and Denham 2010, 2015). As both archaeologists and linguists have suggested, alluding to the spread of the early Metal Age in Europe, it may be that what began to spread across ISEA around 4000 years ago was primarily a new way of thinking—the adoption of a new ideology and perhaps even a new religion (Blench 2012; Spriggs 2011).

## Electronic supplementary material

Supplementary material 1 (PDF 2272 kb)

Supplementary material 2 (XLS 415 kb)
