# Supplementary material for: Resolving the ancestry of Austronesian-speaking populations
Source: Hum Genet. 2016 Jan 18;135:309–26. doi: 10.1007/s00439-015-1620-z (PMC4757630; doi:10.1007/s00439-015-1620-z)
Supplement: Supplementary file 1 — Supplementary material 1 (PDF 2272 kb) [file 439_2015_1620_MOESM1_ESM.pdf]

Supplementary Material 1

Resolving the ancestry of Austronesian-speaking populations

Pedro A. Soares[1,2,3], Jean A. Trejaut[4], Teresa Rito[2,5,6], Bruno Cavadas[2,7], Catherine Hill[3], Ken Khong Eng[3,8], Maru Mormina[3,9], Andreia Brandão[2,,7,10,11], Ross M. Fraser[12,13], Tse-Yi Wang[4], Jun-Hun Loo[4], Christopher Snell[3], Tsang-Ming Ko[14], António Amorim[2,7,15], Maria Pala[10], Vincent Macaulay[16], David Bulbeck[17], James F. Wilson[12,18], Leonor Gusmão[2,19], Luísa Pereira[2,7,20], Stephen Oppenheimer[21], Marie Lin[4], Martin B. Richards[3,10]

[1]CBMA (Centre of Molecular and Environmental Biology), Department of Biology, University of Minho, Braga, Portugal

[2]IPATIMUP (Institute of Molecular Pathology and Immunology of the University of Porto), Porto, Portugal

[3]Faculty of Biological Sciences, University of Leeds, Leeds, United Kingdom

[4]Molecular Anthropology and Transfusion Medicine Research Laboratory, Mackay Memorial Hospital, Taipei, Taiwan

[5]Life and Health Sciences Research Institute (ICVS), School of Health Sciences, University of Minho, Braga, Portugal

[6]ICVS/3B's - PT Government Associate Laboratory, Braga/Guimarães, Portugal

[7]I3S - Institute for Research Innovation in Health, University of Porto, 4200-135 Porto, Portugal

[8]Centre for Global Archaeological Research, Universiti Sains Malaysia, 11800 USM Penang, Malaysia

[9]Department of Applied Social Studies, University of Winchester, UK

[10]Department of Biological Sciences, School of Applied Sciences, University of Huddersfield, Queensgate, Huddersfield, United Kingdom

[11]ICBAS - Institute of Biomedical Sciences Abel Salazar. University of Porto, 4050-313 Porto, Portugal

[12]Centre for Population Health Sciences, University of Edinburgh, Edinburgh, UK

[13]Synpromics Ltd, Nine Edinburgh Bioquarter, Edinburgh, EH16 4UX, UK

[14]Department of Obstetrics and Gynecology, National Taiwan University, Taipei

[15]Faculty of Sciences, University of Porto, Portugal

[16]Department of Statistics, University of Glasgow, Glasgow, United Kingdom

[17]Department of Archaeology and Natural History, College of Asia and the Pacific, The Australian National University, Canberra, Australia

[18]MRC Human Genetics Unit, Institute of Genetics and Molecular Medicine, University of Edinburgh, Western General Hospital, Edinburgh, EH4 2XU, Scotland

[19]DNA Diagnostic Laboratory (LDD), State University of Rio de Janeiro (UERJ), Rio de Janeiro, Brazil.

[20]Faculty of Medicine, University of Porto, Portugal

[21]University of Oxford, Institute of Social and Cultural Anthropology, UK

Corresponding author: Professor Martin B. Richards; Department of Biological Sciences, School of Applied Sciences, University of Huddersfield, Queensgate, Huddersfield, HD1 3DH, United Kingdom; email: m.b.richards@hud.ac.uk; Telephone number: +44 1484 471676

Figure S1. Y-chromosome tree of the SNPs analysed. The embedded table indicates the distribution of the haplogroups across the sampled area.

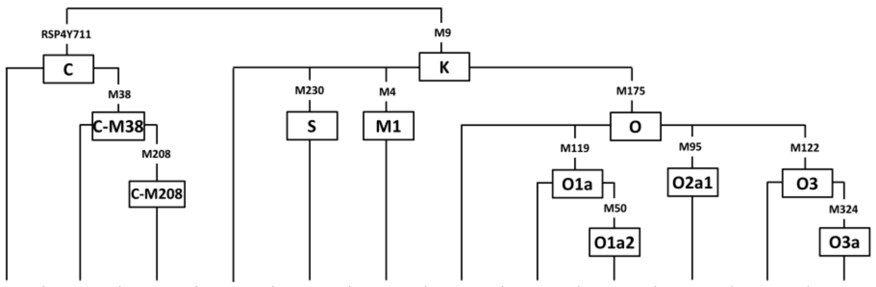

Location/Group													n
S. China							2	4		1		16	23
Ami								9	14	2	2	22	49
Atayal								27	8				35
Bunun								4	17	4			25
Paiwan							1	44	19			1	65
Yami								7		3			10
Philippines								7	1		2	13	23
Pekanbaru	1							10	3	8	2	7	31
Kota Kinabalu	3	3						11	7	7	3	10	44
Banjarmasin	8			3				1	2	7		4	25
Palu		2			3	1		4		5		13	28
Toraja	1	2		1	5	3		4	16	8		8	48
Mataran	3	2		2	2	2		3	4	16		4	38
Alor		21		4		11		1	2			7	46
Madang		3	9	22	10	28				1			73
Lihir		10		21	2	2			2				41
Kavieng		4		7	1	13			1			4	26
Vanuatu		9	5	33	7	10		1	2			3	70
Fiji	2	4	8	6		3						3	26
Atiu			33	3								1	37
French Polynesia			18	4				1				11	34
Total													797

Figure S2. Overall Y-chromosome STR network, calculated using the median-joining algorithm. SNPs were not included in the phylogenetic reconstruction and the samples were labelled according to their SNP lineage after the network construction, to test the robustness of the phylogeny.

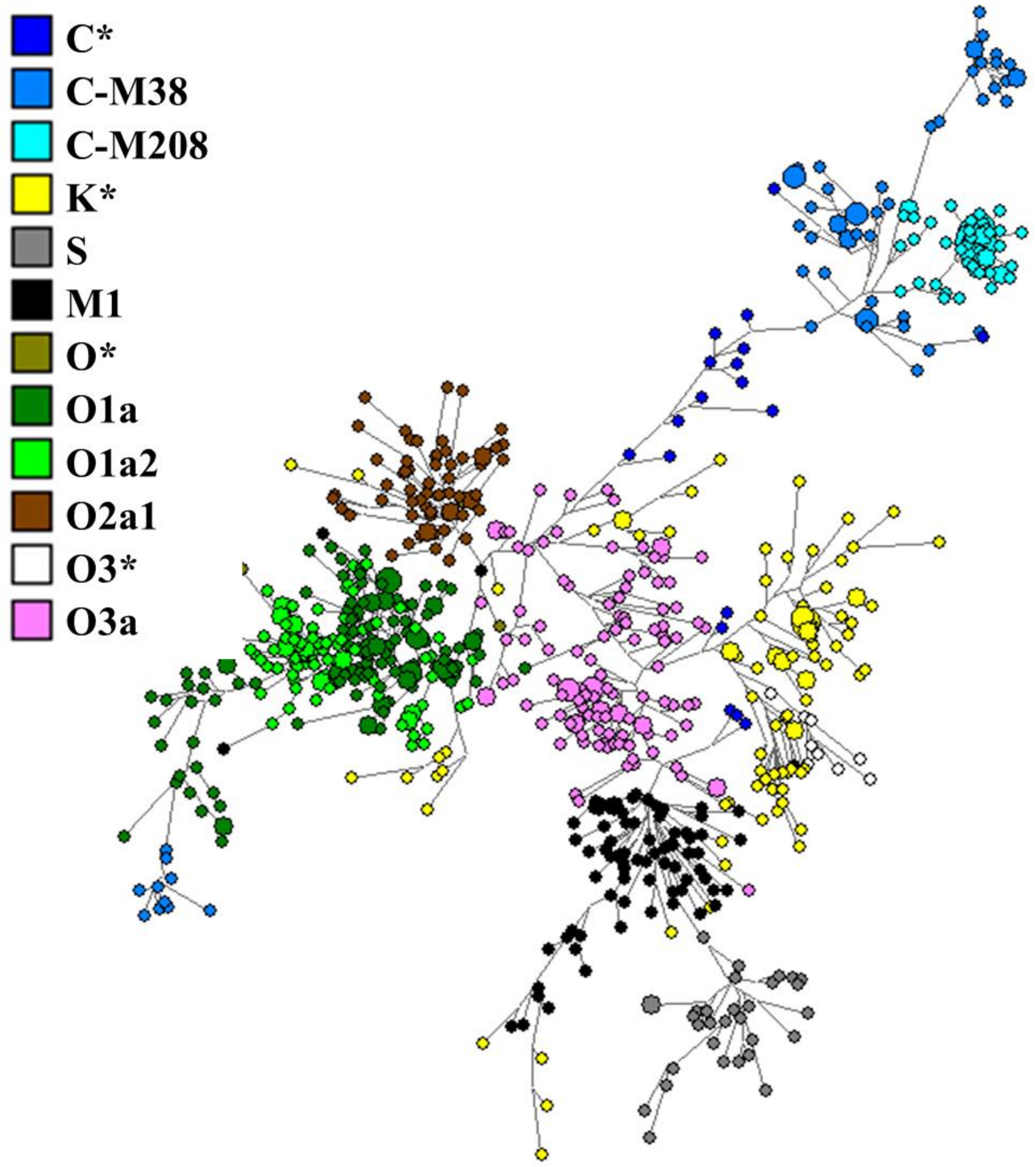

Figure S3. STR network of haplogroup C-M208, indicating the subclade that is exclusive to the Remote Pacific

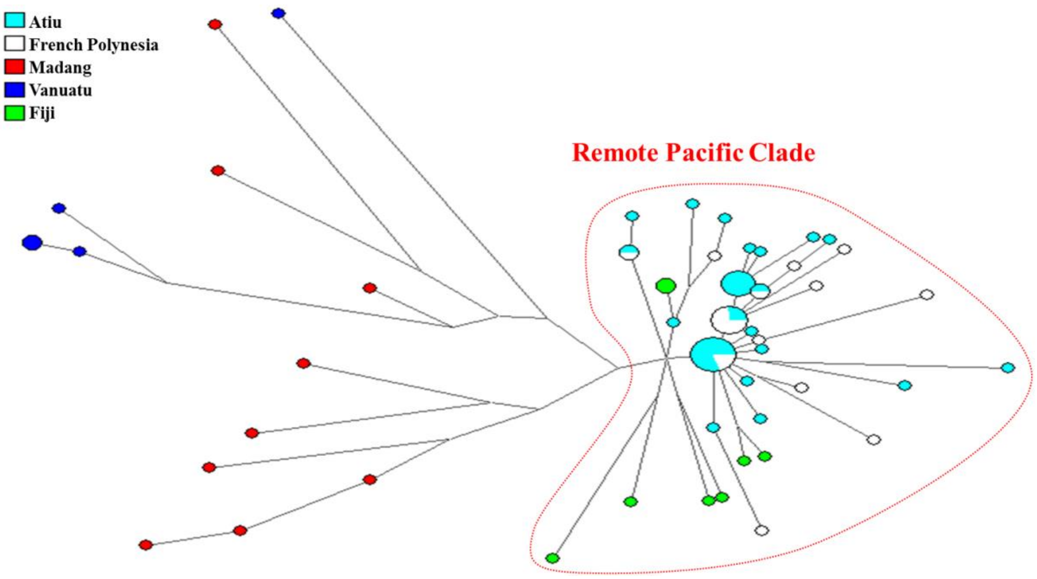

Figure S4. STR network of haplogroup O1*. A subclade displaying a deeper ancestry in ISEA than the remainder of the haplogroup is indicated as indicated by the founder analysis.

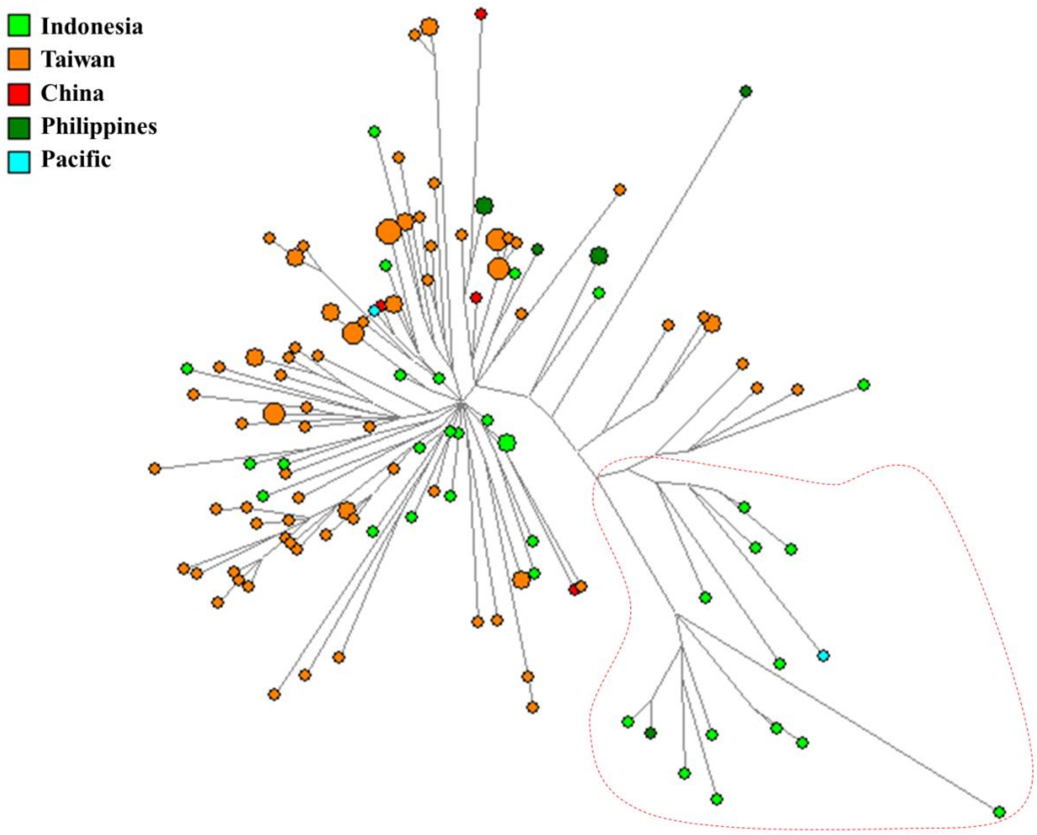

Figure S5. Scan of migration time from ISEA/Near Oceania into Remote Oceania using both Y-chromosome and mtDNA variation

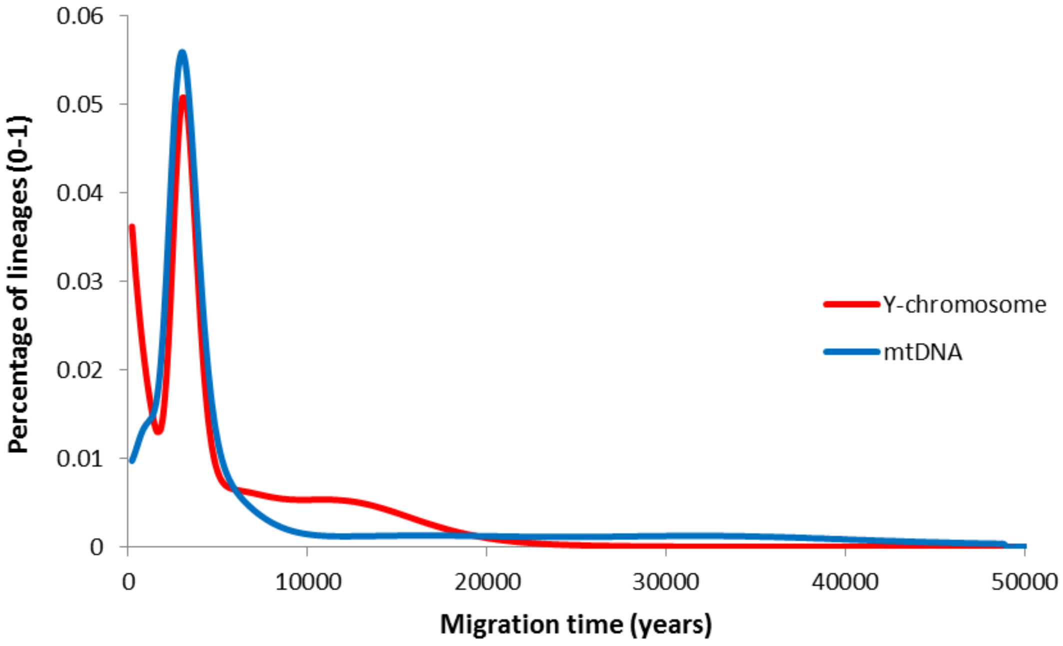

Figure S6. Plot of cross-validation errors across different analyses of ADMIXTURE, against different numbers of ancestral populations (K)

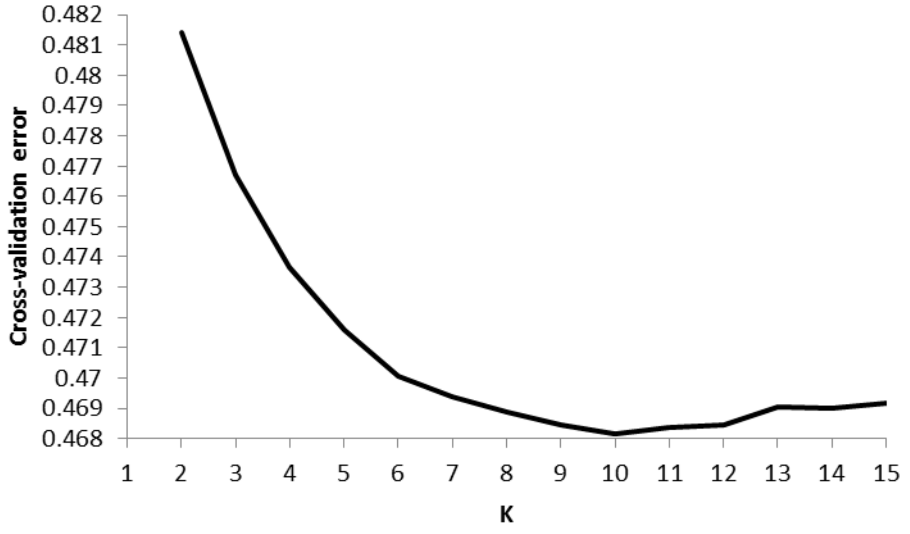

Figure S7. Data points used in the Surfer software for obtaining the frequency distribution of mtDNA clades (A) and autosomal components (B). The outline map was obtained from www.outline-world-map.com.

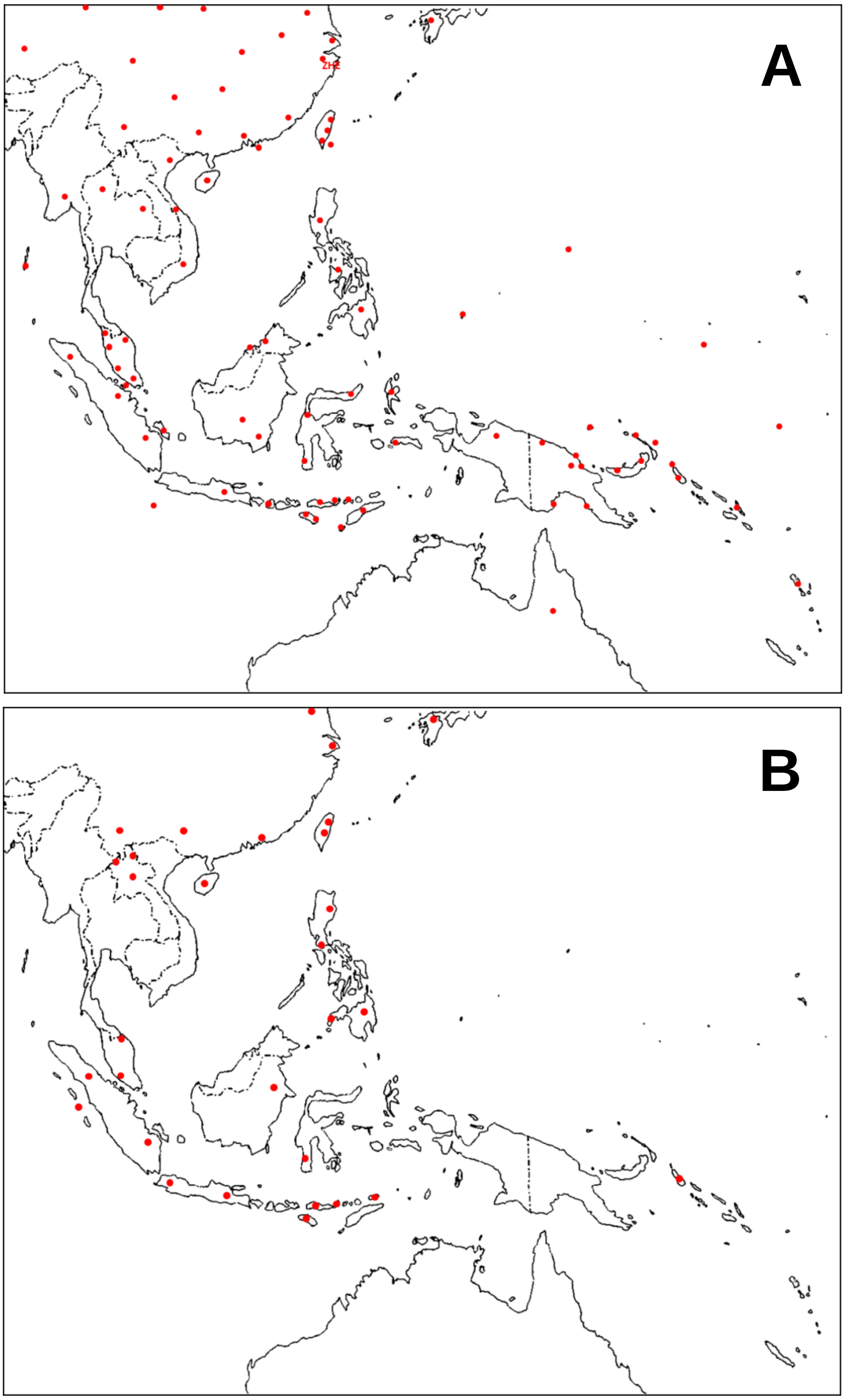

Figure S8. Frequency distribution maps of the two East Asian components obtained on the A*DMIXTURE* analysis when five ancestral populations were considered. The outline map was obtained from www.outline-world-map.com.

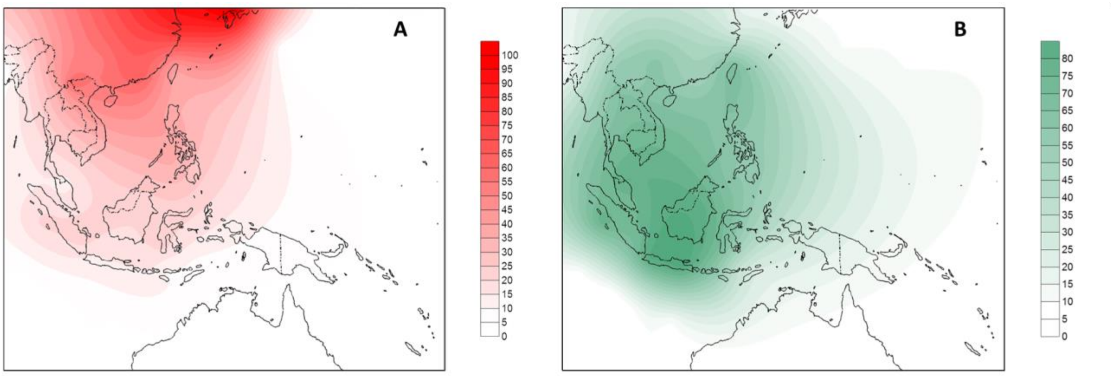

Figure S9. Frequency distribution map of an Island Southeast Asian/Taiwanese component obtained on the ADMIXTURE analysis when 10 ancestral populations were considered. The outline map was obtained from www.outline-world-map.com.

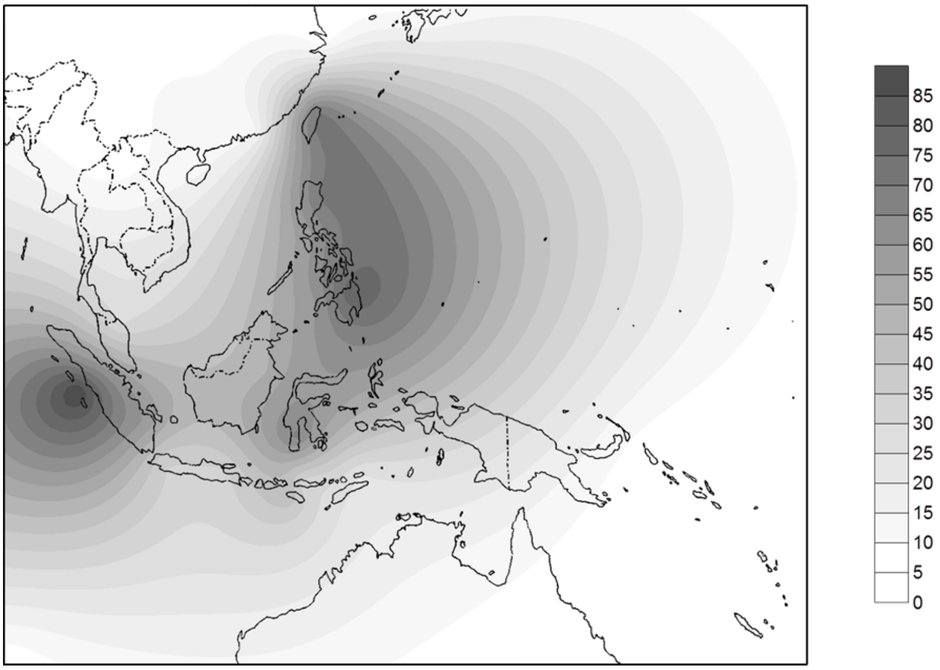

Figure S10. Bayesian skyline plots (BSPs) for haplogroups B4a1a, E and M7c3c in ISEA and Taiwan

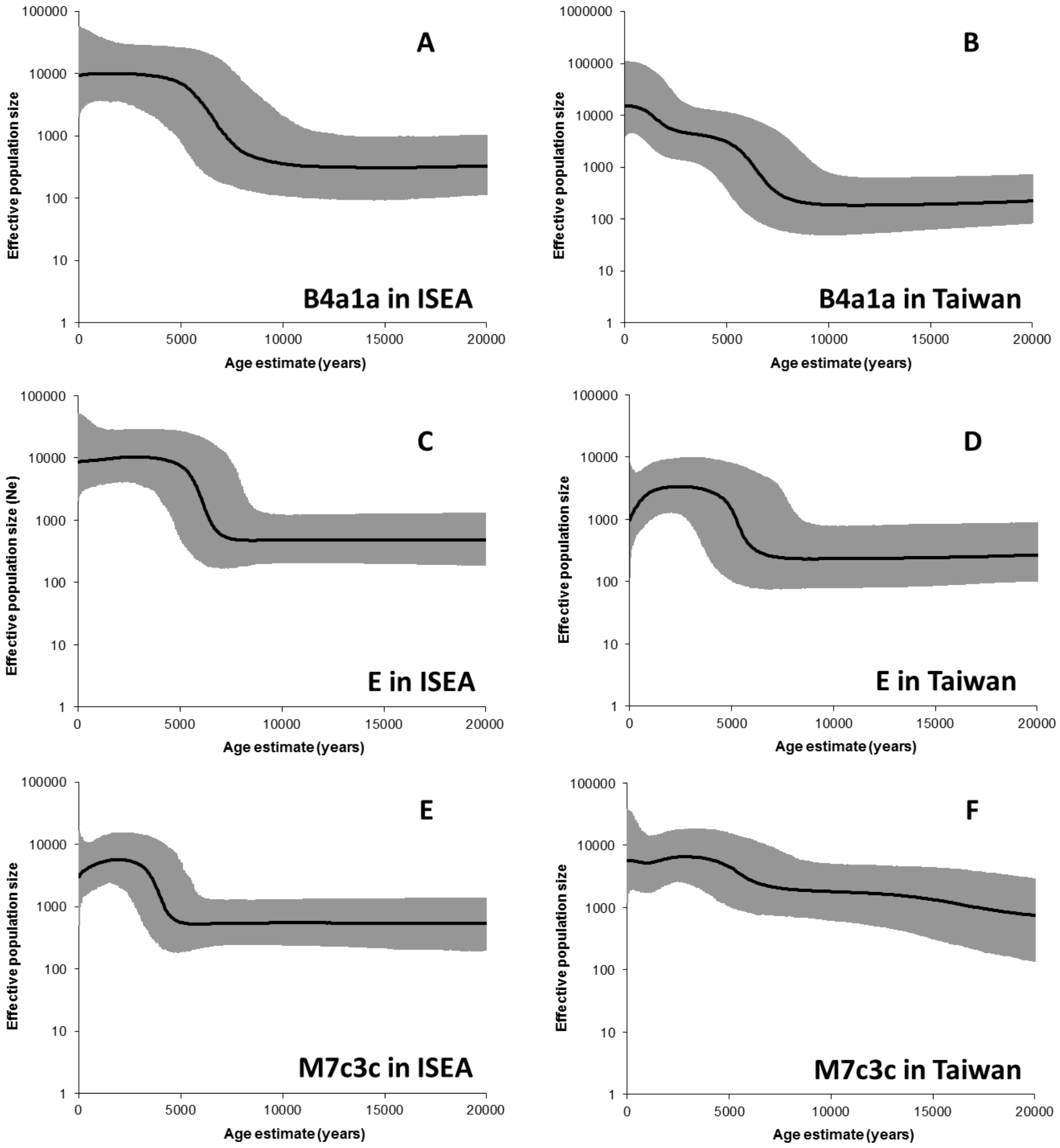

Table S1. Source and sink mtDNA HVS-I datasets employed in the mtDNA founder analysis into ISEA

**Region**	**Sub-region/group**	***n***	**Reference**
		**Source**	
China	Beijing	40	[1]
	Guangxi	1138	[2-6]
	Guizhou	355	[3; 7; 8]
	Hainan	162	[3]
	Zheijiang	61	[6]
	Manchurian	40	[1]
	Northern Han	60	[9]
	Qinghai	171	[2; 6; 10; 11]
	Shanghai	193	[3; 6; 12]
	Yunnan	1238	[2; 3; 5; 6; 10; 13-16]
	Xinjiang	214	[16-18]
	Hunan	291	[5; 6; 19]
	Fujian	54	[6]
	Inner Mongolia	200	[6; 13]
	Liaoning	102	[6; 16]
	Jilin	106	[20; 21]
	Shandong	50	[16]
	Gangsu	128	[6; 11]
	Guangdong	631	[3; 5; 16; 22-24]
	Anhui	42	[6]
	Jiangsu	67	[6]
	Jiangxi	23	[6]
	Shaanxi	123	[6; 19]
	Hubei	52	[3; 16]
	Hong Kong	397	[25; 26]
	Sichuan	132	[6; 11]
Tibet	-	452	[11]
Mongolia		199	[1; 18; 27]
Central Asia	Kazakhstan	108	[18; 28]
	Kyrgyzstan	149	[28]
	Tuvan	36	[29]
Japan	-	1721	[9; 12; 19; 30-34]
Korea	-	64	[35; 36]
	South	583	[1; 30; 37]
North Asia	Tuvan	102	[38; 39]
	Tofalar	31	[38; 39]
	Todjins	26	[38]
	Sojots	15	[38]
	Khakassians	30	[38]
	Buryat	231	[9; 38-40]
	Altai	54	[38]
	Tubalar	26	[39]
	Evenk	15	[29; 35; 39]
	Yakuts	104	[29; 40]

	Ulchi	43	[39]
	Udegey	16	[35; 39]
	Nivkh	78	[9; 35; 39]
	Koryak	257	[9; 41]
	Yukaghirs	15	[29]
	Chukchi	72	[42; 43]
	Eskimo	83	[42; 43]
	ItelÆmen	46	[41]
	Negidal	17	[39]
Taiwan	Yami	84	[44; 45]
	Han	66	[30]
	Paiwan	97	[44-46]; Unpublished
	Rukai	70	[44; 45]
	Puyuma	72	[44; 45]
	Ami	149	[44-46]; Unpublished
	Tsou	80	[44; 45]
	Bunun	129	[44-46]; Unpublished
	Saisiat	83	[44; 45]
	Atayal	147	[44-46]
Thailand	Northwest	354	[47; 48]; Unpublished
	Hill Tribe	58	[49]
	Central/ Lao Song/ Phuthai	90	[1]
	Chantaburi	24	[47]
	Trang	20	[47]
	North Thailand	32	[2]
	Khon Kaen/ Mukdahan	94	[47]
	Chong	24	[47]
	Mussur	21	[47]
Vietnam	South Vietnam	211	[3; 19]; Unpublished
	Central Vietnam	58	[3]
	North (Hanoi)	443	[1; 50]; Unpublished
Burma	-	378	Unpublished
	**Sink**		
Borneo	Brunei	30	Unpublished
	Palangkaraya	112	Unpublished
	Kota Kinabalu	109	[46]; Unpublished
	Banjarmasin	89	[46]
Indonesia	Adonara/Lembata	111	[51]
	Alor/Pantar	165	[46; 51]; Unpublished;
	Ambon	72	[46]; Unpublished
	Bali	99	[46]; Unpublished
	Bangka	34	[52]
	East Timor	38	[51]
	Flores	84	[51]; Unpublished
	-	54	[9]
	Lombok	74	Unpublished; [46]
	Manado	89	[46]
	Medan	45	[46; 53]
	Moluccas/Nusa Tenggaras	61	[54]
	Padang	25	[52]; Unpublished

	Palu	38	[46]
	Pekanbaru	56	[52; 53]
	Solor	41	[51]
	Palembang	37	[46]; Unpublished
	Jawa Timur	36	[46]
	Toraja/ Ujung Padang	110	[46]
	Waingapu - Sumba	51	[46]; Unpublished
Philippines	Luzon	47	[55]
	Mindanao	27	[55]
	Visayas	26	[55]
	Undetermined	456	[9; 46; 55]; Unpublished

Table S2. Additional data compiled and eventually used to refine the topology of the HVS-I networks but not employed either as source or sink population in any analysis

**Region**	**Sub-region/group**	***n***	**Reference**
Andaman islands	Great Andamanese	20	[56]
	Jarawa	4	[56]
	Onge	63	[56]
Australia	Unknown	54	[54]
	Darling River, West	63	[57]
	Kimberley of Western Australia	2	[25]
	western desert of Western Australia	2	[25]
	Yuendumu, Central Australia	51	[57]
	northwestern Australia	32	[58]
Singapore	-	55	Unp.
Malaysia	Johor	71	[59; 60]
	Kedah/Perlis/Penang	52	[59; 60]
	Perak	67	[59; 60]
	Kelantan/Terengganu	106	[59; 60]
	Selangor/Wilayah/Negeri/Melaka	223	[59-61]
Orang Asli	Malaysia	288	[53]; Unp
	Sakai	20	[47]
Christmas Islands	Christmas Islands	70	[62]
Micronesia	Guam	40	Unpublished
	Nauru	34	Unpublished
	Kiribati	14	Unpublished

Table S3. Source and sink mtDNA HVS-I datasets employed in the mtDNA founder analysis into Remote Oceania. Both source and sink populations in Table S1 are included in the source for this analysis.

**Region**	**Sub-region/group**	***n***	**Reference**
		**Source**	
Karkar Islands		47	[63]
New Guinea	Simbu/Western Highlands	16	Unpublished
	Bundi	58	[58]; Unp
	Irian Jaya	178	Unpublished
	Southern Highlands	17	Unpublished
	Sepik Province	219	[64]
	Port Moresby	117	Unpublished
	Madang	163	Unpublished
	Undetermined	78	[54]; Healy and Hunley (GenBank direct submission); Unpublished
Bismarck Archipelago	Balopa	59	[65]
	East New Britain	222	[66]
	West New Britain	353	[66]
	Lavongai	18	[66]
	Kavieng	83	Unpublished
	Lihir	94	Unpublished
	New Ireland Papua	62	[66]
	North New Ireland Astronesian	98	[66]
	Mussau	16	[66]
Bougainville	South	109	[66]
	North	91	[66]
	Central - Aita	33	[66]
	-	22	Healy and Hunley (GenBank direct submission); Unpublished
Solomon Islands	Malaita	237	[66]
	-	21	Unp
		**Sink**	
Vanuatu	-	130	[67]; Unpublished
New Zealand	-	13	Pierson and Fris (GenBank direct submission); Unpublished
Cook Islands		27	Pierson and Fris (GenBank direct submission); Unpublished
Fiji	-	1	Pierson and Fris, 2006
Tonga	-	51	[65]; Pierson and Fris (GenBank direct submission);
Samoa	-	39	[54]; Pierson and Fris (GenBank direct submission);
French Polynesia	Mangareva	17	[68], Unpublished

Table S4. Primers used in the typing of ten Y-STRs, including the fluorescence label for each forward primer (FAM, TET, HEX). References are provided when the primers were taken from the literature.

STR	Forward Primer	Reverse Primer
DYS460	FAM-AGCAAGCACAAGAATACCAGAG [69]	TCTATCCTCTGCCTATCATTTATTA [70]
DYS461	FAM-AGGCAGAGGATAGATGATATGGAT [70]	TGATGCTGTGTCACTATATTTCTG [69]
DYS438	FAM-TGGGGAATAGTTGAACGGTAA [71]	GTGGCAGACGCCTATAATCC [71]
DYS448	FAM- TGTCAAAGAGCTTCAATGGAGA (*)	TCTTCCTTAACGTGAATTTCCTC (*)
DYS425	TET- TGGAGAGAAGAAGAGAGAAAT (*)	AGTAATTCTGGAGGTAAAATGG (*)
DYS458	TET-GCAACAGGAATGAAACTCCAAT (*)	GTTCTGGCATTACAAGCATGAG (*)
DYS437	TET-GACTATGGGCGTGAGTGCAT [71]	AGACCCTGTCATTCACAGATGA [71]
DYS439	TET-TCCTGAATGGTACTTCCTAGGTTT [71]	GCCTGGCTTGGAATTCTTTT [71]
GATA-H4	TET-GTTATGCTGAGGAGAATTTCCAA [69]	CCTCTGATGGTGAAGTAATGGAATTAGA [70]
DYS388	HEX – GTGAGTTAGCCGTTTAGCGA (*)	CAGATCGCAACCACTGCG (*)
GATA-A10	HEX-CCTGCCATCTCTATTTATCTTGC (*)	TGGAGATAGTGGGTGGATTGA(*)
DYS635	HEX-AGTGTCTCACTTCAAGCACCAAGCAC [70]	GCAGCAAAATTCACAGTTGGAAAAATGT [70]

(*) Newly designed primer

Table S5. Primers and restriction enzymes used in the typing of three Y-chromosome SNPs.

SNP	Forward Primer	Reverse Primer	Restriction Enzyme
M208	GCAACGATTTATCAGCTTTCA	GCAGGAAAAGCCTGTTTGTT	*TaqI*
M230	AATGTCACATTTAGTCTTAACCCAT	ACATTATTAGTATGTAAATCTTCATTGC	*Tsp5091*
M324	TGATAGAAGGCAAGAGGGAGT	AACAAATTGATTTCCAGGGATA	*MnlI*

Table S6. Samples used in the ADMIXTURE analysis

Population	code	n	Ethnicity	Location
Yoruba	YRI	60	Yoruba	Nigeria
India	IN-WI	25	Caucasoids	Rajasthan, India
	IN-WL	14	Caucasoids	Maharashtra, India
Japanese	JP-ML	71	Japanese	Tokyo, Japan
	JPT	44	Japanese	Tokyo, Japan
Koreans	KR-KR	90	Koreans	Gyunggi-province, Korea
Han	CN-SH	21	Han	Shanghai, China
Han	CHB	45	Han	Beijing, China
Chinese in	TW-HA	48	Chinese	Taipei, Taiwan

Taiwan	TW-HB	32	Chinese	Taipei, Taiwan
Han	CN-GA	30	Han	Guangzhou, China
Zhuang	CN-CC	26	Zhuang	Guangxi, China
Jiamao	CN-JI	31	Jiamao	Hainan, China
Wa	CN-WA	29	Wa	Yunnan, China
Wa	CN-WA	27	Wa	Yunnan, China
Jinuo	CN-JN	29	Jinuo	Yunnan, China
Yao	TH-YA	19	Yao	Chiang Rai province, Thailand
				Phayao province, Thailand
				Nan province, Thailand
Paluang	TH-PL	18	Paluang	Chiang Mai province, Thailand
Karen	TH-KA	20	Karen	Mae Hong Son province, Thailand
				Chiang Mai province, Thailand
Lawa	TH-LW	19	Lawa	Mae Hong Son province, Thailand
Tai	TH-TU	20	Tai Yuan	Lamphun province, Thailand
				Chiang Mai province, Thailand
				Saraburi province, Thailand
	TH-TY	18	Tai Yong	Lamphun province, Thailand
	TH-TL	20	Tai Lue	Nan province, Thailand
				Chiang Mai province, Thailand
	TH-TK	18	Tai Khuen	Chiang Mai province, Thailand
Ami	AX-AM	10	Ami	Taiwan
Atayal	AX-AT	10	Atayal	Taiwan
Filipino	PI-UB	20	Filipino	Isabela Province, The Philippines
Filipino	PI-UN	19	Filipino	Metro Manila, The Philippines
Minanubu	PI-MA	18	Minanubu	Loreto, Agusan del Sur, The Philippines
Filipino	PI-UI	20	Filipino	Zamboango, The Philippines
Proto-Malay	MY-TM	49	Proto-Malay	Jelebu District, Negri Sembilan, Malaysia
			Proto-Malay	Kuala Pilah District, Negri Sembilan, Malaysia
Malay	MY-KN	18	Malay	Jeli (Dabung), Machang, Kelantan, Malaysia
Malay	MY-MN	20	Malay	Lenggeng, Negeri Sembilan, Malaysia
Dayak	ID-DY	12	Dayak	East Kalimantan, Indonesia
Batak	ID-TB	20	Batak Toba	Balige, Sumatra, Indonesia
	ID-KR	17	Batak Karo	Karo, North Sumatra, Indonesia
Malay	ID-ML	12	Malay	Pelembang, South Sumatra, Indonesia
Mentawai	ID-MT	15	Mentawai	Mentawai Island, Indonesia
Sunda	ID-SU	25	Sunda	Jakarta, Java, Indonesia
Javanese	ID-JV	19	Javanese	Java, Indonesia
	ID-JA	34	Javanese	Jakarta, Java, Indonesia
Toraja	ID-TR	20	Toraja	Tana Toraja, Sulawesi, Indonesia
Kambera	ID-SB	20	Kambera	Sumba Timur, Indonesia
Manggarai	ID-SO	19	Manggarai	Ngada, Flores, Indonesia
	ID-RA	17	Manggarai	Rampasasa, Manggarai, Indonesia
Lamaholot	ID-LA	20	Lamaholot	Larantuka, East Flores, Indonesia
Alorese	ID-AL	19	Alorese	Alor Island, Indonesia
Lembata	ID-LE	19	Lembata	Lembata, East Flores, Indonesia
Melanesians	AX-ME	5	Melanesians	Indo-Pacific

Table S7. M7 sequences used in the phylogenetic reconstruction

Sequence (accession number / Code)	Location/ group	Reference
AP008249, AP008266, AP008270, AP008274, AP008280, AP008282, AP008295, AP008297, AP008299, AP008310, AP008316, AP008327, AP008330, AP008336, AP008341, AP008350, AP008351, AP008354, AP008359, AP008365, AP008367, AP008372, AP008376, AP008387, AP008394, AP008402, AP008404, AP008405, AP008429, AP008439, AP008455, AP008466, AP008469, AP008483, AP008485, AP008503, AP008507, AP008509, AP008514, AP008517, AP008541, AP008548, AP008555, AP008571, AP008585, AP008586, AP008588, AP008592, AP008600, AP008621, AP008625, AP008643, AP008647, AP008653, AP008671, AP008686, AP008689, AP008695, AP008699, AP008711, AP008721, AP008725, AP008728, AP008729, AP008731, AP008734, AP008750, AP008755, AP008758, AP008779, AP008794, AP008797, AP008799, AP008886, AP008887, AP008902, AP008913, AP009420, AP009421, AP009423, AP009427, AP009435, AP009443, AP009451, AP009459, AP009466, AP010685, AP010979, AP010986, AP010993, AP010996, AP011009, AP011022, AP011039, AP011048	Japan	[72]
AP010661, AP010672, AP010680, AP010681, AP010692, AP010698, AP010717, AP010719, AP010730, AP010739, AP010747, AP010750, AP010758, AP010763	Japan	[73]
AP010824, AP010825, AP010826, AP010827, AP010997	Japan	[33]
AP012360, AP012363	East Malaysia (Borneo)	[74]
AP012419, AP012426	Peninsular Malaysia	[74]
AY255146	Inner Mongolia	[75]
AY255158	Liaoning, China	[75]
AY255159	Hunan, China	[75]
AY255171	Shandong, China	[75]
AY255173	Xinjiang, China	[75]
AY289097, AY289098	Taiwanese Indian	[76]
DQ272117, DQ272126	China - Guizhou	[77]
DQ372868	Taiwan	[78]
DQ372876	Micronesia: Majuro Atoll	[78]
EF153777, EF153781, EF153782, EF153789,EF153790, EF153817, EF153818, EF153820	South Siberia	[79]
EF153810	Czech Republic	[79]
EF153823, EF397561	South Korea	[79]
EU007890	Mongolia	[80]
EU597541	China	[81]
FJ748706, FJ748715	Tibet	[82]
GQ119018, GQ119023	Philippines	[55]
GU123012	Volga-Ural - Russia	[83]
GU392071, GU392103	China	[84]
GU733735, GU733736	Philippines - Mamanwa	[85]

GU733762, GU733766, GU733767, GU733771, GU733772, GU733777, GU733788, GU733792, GU733799	Philippines - Manobo	[85]
GU733804	Philippines - Surigaonon	[85]
GU810069	Sea nomads of Thailand	Unp.
HG00403, HG00410, HG00448, HG00501, HG00512, HG00524, HG00525, HG00593, HG00611, HG00650, HG00689, HG00692, HG00701	South Han Chinese	[86; 87]
HG00759, HG01028, HG01029, HG01810, HG01817, HG02156, HG02166, HG02180, HG02185, HG02187, HG02355, HG02367, HG02371, HG02384, HG02389, HG02390, HG02396, HG02401	Chinese Dai in Xishuangbanna	[87]
HG01596, HG01599, HG01840, HG01841, HG01843, HG01846, HG01851, HG01861, HG01871, HG02019, HG02031, HG02046, HG02048, HG02057, HG02060, HG02067, HG02075, HG02079, HG02084, HG02085, HG02088, HG02121, HG02127, HG02137, HG02141	Kinh in Ho Chi Minh City, Vietnam	[87]
HM030506	China -Sichuan	[88]
HM030509, HM030514, HM030523	China - Yunnan	[88]
HM030527,HM030547	China- Guangxi	[88]
HM030531	China - Qinghai	[88]
HM030532	China - Guizhou	[88]
HM238203, HM238206	Philippine Islanders - Ivatan	[89]
HM238210, HM238218	Orchid Islands - Yami	[89]
HM357815, HM357816, HM357819, HM357821	China- Guangxi	[90]
HM596649, HM596650, HM596659, HM596662, HM596663, HM596664, HM596668, HM596669, HM596673, HM596674, HM596678, HM596685, HM596714	Sumatra	[91]
HM852807	Azeri	[92]
HQ157976, HQ157980, HQ157984	China - Hainan	[93]
JQ705503	Japan	[94]
JQ702069, JQ702126, JQ703812, JQ705461, JQ705619	Unknown	[94]
JQ702664, JQ704806, JQ705375	China	[94]
JQ703844	Philippines	[94]
JX390633	Philippines	FT- DS
KC993909, KC993919, KC993930	Philippines - Abaknon	[95]
KC993937	Philippines - Aeta_Bataan	[95]
KC993974, KC993977, KC993981, KC993983, KC993984, KC993985, KC993987, KC993993, KC993995, KC993998, KC994002	Philippines - Bugkalot	[95]
KC994005, KC994006, KC994008, KC994011, KC994012, KC994013, KC994016, KC994025, KC994026	Philippines - Ibaloi	[95]
KC994032, KC994033, KC994034, KC994038, KC994041, KC994044, KC994051, KC994052	Philippines - Ifugao	[95]
KC994065, KC994071, KC994079	Philippines - Ivatan	[95]
KC994088, KC994089, KC994090, KC994096, KC994098, KC994099, KC994100, KC994102, KC994103, KC994106,	Philippines - Kalangoya	[95]

KC994110, KC994113		
KC994116, KC994121, KC994122, KC994126, KC994133, KC994142	Philippines - Kankanaey	[95]
KC994153, KC994158	Philippines - Maranao	[95]
KF540506, KF540507, KF540510, KF540511, KF540518, KF540526, KF540527, KF540531, KF540535, KF540536, KF540537, KF540539, KF540540, KF540545, KF540546,KF540548, KF540553, KF540555	Taiwan - Ami	[96]
KF540556, KF540557, KF540562, KF540564, KF540565, KF540567, KF540570, KF540571, KF540574, KF540576, KF540578, KF540579, KF540580, KF540583, KF540589, KF540593, KF540597, KF540598, KF540602, KF540604	Taiwan - Atayal	[96]
KF540606, KF540609, KF540631	Taiwan - Bunun	[96]
KF540664, KF540667, KF540669, KF540687, KF540700	Taiwan - Hakka	[96]
KF540705, KF540710, KF540721, KF540728, KF540731, KF540736, KF540742, KF540749, KF540750	Taiwanese Han	[96]
KF540753, KF540754, KF540759, KF540760, KF540762, KF540772, KF540776, KF540781, KF540782, KF540790, KF540791, KF540793	Taiwan - Paiwan	[96]
KF540801, KF540809, KF540820, KF540823, KF540826, KF540828, KF540833, KF540846, KF540849	Taiwan - Makatao	[96]
KF540858, KF540863, KF540877, KF540879	Taiwan - Puyuma	[96]
KF540892, KF540907, KF540912	Taiwan - Rukai	[96]
KF540942, KF540946, KF540951, KF540953, KF540955, KF540958, KF540960, KF540961, KF540963	Taiwan - Saisiat	[96]
KF540966, KF540977, KF540980, KF540982	Taiwan - Tao	[96]
KF541008, KF541015, KF541048, KF541052, KF541053	Taiwan - Tsou	[96]
KC252344, KC252345, KC252348, KC252349, KC252350, KC252351, KC252353, KC252365, KC252371, KC252378, KC252379, KC252397, KC252398, KC252402, KC252406, KC252421, KC252427, KC252428, KC252431, KC252433, KC252439, KC252448, KC252455, KC252456, KC252458, KC252461, KC252462, KC252463, KC252468, KC252470, KC252471, KC252473, KC252479, KC252480, KC252483, KC252484, KC252490, KC252491, KC252501, KC252505, KC252509, KC252510, KC252523, KC252527, KC252531, KC252537, KC252552, KC252553, KC252555, KC252558, KC252559, KC252569, KC252573	South Taiwan (mixed)	[97]
KJ154325	Solomon Islands: Tuvalu	[98]
KJ154750, KJ154751, KJ154752, KJ154753, KJ154754, KJ154755, KJ154756, KJ154757	Solomon Islands: Ontong Java	[98]
KJ154775,KJ154941	Solomon Islands: Vella Lavella	[98]
NA17969, NA18126, NA18138, NA18149, NA18152, NA18674, NA18707	Chinese in Denver, USA	[86]
NA17971, NA18124, NA18550, NA18574, NA18582, NA18618, NA18636, NA18638, NA18639, NA18644, NA18756, NA18769, NA18771	Han Chinese in Beijing	[86]
NA18755	Beijing Han Chinese	[86]
NA18940, NA18943, NA18952, NA18953, NA18965,	Japan	[86]

NA18999, NA19001, NA19075, NA19548, NA19558, NA19566, NA19573		
NA19011, NA19090	Japan	[87]
SSM041, SSM047, SSM057, SSM062, SSM072, SSM076, SSM086	Malaysia	[99]
BRU18, BRU49, BRU53	Brunei (Borneo)	This study
BUR1	Myanmar	This study
Fuj5274, FujP91043M	China - Fujian	This study
HA056, HA064	Hakka	This study
ALO193, ALORX	Indonesia - Alor	This study
BAL38	Indonesia - Bali	This study
BAN4	Indonesia - Banjarmasin (Borneo)	This study
IN159, IN170, In197, IN246, IN251, IN370	Indonesia - Java	This study
MND48	Indonesia -Manadu	This study
PAD11	Indonesia - Padang	This study
PRY100, PRY65	Indonesia - Palangkaraya (Borneo)	This study
WAI48, WAI56	Indonesia - Waigapu (Sumba)	This study
LAO236, LAO245, LAO276, LAO318, LAO419, LAO442	Laos	This study
AC06	Malaysia - Acheh - Kedah Yan	This study
BJ120, BJ136	Malaysia - Banjar-Perak Kuala Kurau	This study
BG104	Malaysia - Bugis- Johor Pontian	This study
JW78	Malaysia - Johor Muar - Jawa	This study
JW73	Malaysia - Johor Semerah - Jawa	This study
MB15	Malaysia - Kelantan Kota Bahru	This study
RP04, RP26	Malaysia - Kelantan RantauPanjang	This study
KK136, KK172, KK2, KK23, KK48, KK49, KK96	Malaysia - Kota Kinabalu (Borneo)	This study
MI51, MI58	Malaysia - Minangkabau - Negeri Sembilan Lenggeng	This study
100B	Malaysia - Semelai	This study
KB23, KB31	Micronesia - Kiribati	This study
NAU29, NAU31	Micronesia - Nauru	This study
PE003, PE009, PE010, sbb043, SD10362, P91043M, AD269	Taiwan - Minnan	This study
PZ003, PZ022, PZ078, PZ102	Taiwan - Pazeh	This study
PH277	Philippines - Luzon	This study
FIL34	Philippines (general)	This study
PU018	Taiwan - Puyuma	This study
DM006, DM007	Taiwan - Siraya western Plain	This study

	tribe/Pimpu	
Am002, AM009, Am051, AMI21, KA28, KA43, KA65, KA72	Taiwan - Ami	This study
AT033, ATA20	Taiwan - Atayal	This study
BUN20	Taiwan - Bunun	This study
KP24, KP30, Pw034	Taiwan - Paiwan	This study
Sa004, Sa027	Taiwan - Saisiat	This study
SL017, SL273, SL495, SL588	Taiwan - Siraya	This study
AD014, AD183, AD203, AD232	Taiwan Han	This study
Thai142	Thailand	This study
DKX3729, DKX4103, DKX4440, DKX4468, DOX2001, DOX2198, DOX4692, DOX6353, VNM184, VNM201, VNM253, VNM264, VNM271, VNM274, VNM313, VNM363, VNM202, VNM224, VNM237, VNM340	Vietnam	This study

Table S8. M9/E sequences used in the phylogenetic reconstruction

Sequence (accession number)	Location/ group	Reference
AF346972	China	[100]
AP008353, AP008378, AP008629, AP008677, AP008702, AP008704, AP008710, AP008766, AP008815, AP008860, AP008863, AP010662, AP010687, AP010767, AP011019	Japan	[72]
AY255153	Xinjiang, China	[75]
AY289070	Philippines	[76]
AY963582	Malay (Melayu)	[53]
DQ272112	China	[77]
EF061148, EF061150	North New Ireland	[66]
EF061149, EF061151, EF061152	West New Britain	[66]
EF093535, EF093536, EF093537, EF093538	Taiwan - Ami	[101]
EF093539	Taiwan - Atayal	[101]
EF093544, EF185810	Taiwan - Bunun	[101]
EF093552	Taiwan - Puyuma	[101]
EF093553	Taiwan - Saisiat	[101]
EF093540, EF093541, EF093542, EF093543, EF093547, EF093548, EF093549, EF093550, EF093551	Philippines	[101]
EF093545, EF185793	Vietnam	[101]
EF093546	New Guinea	[101]
EF093554, EF093555	Taiwan - Siraya	[101]
EF093556	Taiwan - Thao	[101]
EF093557, EF093558	Taiwan - Tsou	[101]
EF185794	Indonesia - Ambon	[101]
EF185795, EF185796, EF185797, EF185798, EF185799	Indonesia - Banjarmasin	[101]
EF185800	Malaysia - Kota Kinabalu	[101]
EF185801, EF185802, EF185803	Indonesia - Manado	[101]
EF185804, EF185805, EF185806	Indonesia - Ujung Padang	[101]
EF185807, EF185815	Indonesia - Waingapu	[101]
EF185808	Indonesia - Bali	[101]

EF185809	Indonesia - Bangka	[101]
EF185811, EF185812, EF185813	Indonesia - Kota Kinabalu	[101]
EF185814	Indonesia - Toraja	[101]
EF185816	Indonesia - Palangkaraya	[101]
EU007852	Nivkchi; North Asia	[80]
FJ383310, FJ383311, FJ383312, FJ383313, FJ383314, FJ383315, FJ383316, FJ383317, FJ383318, FJ383319, FJ383320, FJ383321, FJ383322, FJ383323, FJ383324, FJ383325, FJ383326, FJ383327, FJ383328, FJ383329, FJ383330	India	[102]
FJ428235, FJ428236	Papua New Guinea	[101]
FJ544236, FJ748723, FJ748729, FJ748735, FJ748743, FJ748744, FJ748755, FJ748758, FJ968772, FJ968774, FJ968775	Tibet	[82]
GQ119027, GQ119043, GQ119047	Philippines	[55]
GQ337542	Bangladesh	[103]
GQ337575	West Bengal	[103]
GQ337588	Himalayas	[103]
GQ895140, GQ895143, GQ895145, GQ895146, GQ895148, GQ895150, GQ895151, GQ895159, GQ895160	Tibet	[104]
GU012637	Philippines	FT-DS
GU014567	Tibet	[11]
GU733721, GU733723, GU733727, GU733741, GU733749, GU733756	Philippines - Mamanwa; negrito group	[85]
GU733757, GU733758, GU733761, GU733763, GU733769, GU733774, GU733775, GU733778, GU733779, GU733780, GU733781, GU733784, GU733789, GU733791	Philippines - Manobo	[85]
GU733806, GU733807, GU733808, GU733809, GU733810, GU733816, GU733820	Philippines - Surigaonon	[85]
GU810007, GU810031, GU810035, GU810036, GU810040, GU810041, GU810063, GU810064, GU810070	sea nomads of Thailand	Unp
HG02081, HG02522	Kinh in Vietnam	[87]
HG02379	Chinese Dai	[87]
HM036540, HM036545, HM036546, HM036547, HM036552, HM036568, HM036569, HM036570, HM036572, HM036573	Great Himalayas	Unp
HM238216	Orchid Islands - Yami	[89]
HM346881, HM346889, HM346886, HM346885, HM346883	Vietnam	[103]
HM346882, HM346932, HM346933	China- Shandong	[103]
HM346884, HM346888	China -Guangxi	[103]
HM346887	China- Hainan	[103]
HM346890, HM346891, HM346912	China- Guangdong	[103]
HM346892	China - Hunan	[103]
HM346893, HM346934	China- Liaoning	[103]
HM346894	China- Gansu	[103]
HM346895, HM346896	Myanmar	[103]

HM346897, HM346902, HM346898, HM346916, HM346917, HM346918, HM346919, HM346920, HM346921, HM346922, HM346923, HM346924, HM346925, HM346926, HM346927, HM346928	Tibet	[103]
HM346899, HM346909, HM346915, HM346931, HM346936	China- Sichuan	[103]
HM346900	India	[103]
HM346901, HM346911	China- Qinghai	[103]
HM346903, HM346913	China - Xinjiang	[103]
HM346904, HM346910, HM346930	China- Yunnan	[103]
HM346905	China- Shaanxi	[103]
HM346906, HM346907, HM346914	China - Henan	[103]
HM346908, HM346929, HM346935	Inner Mongolia	[103]
HM036548	Ladakh tribe of the Great Himalayas	Unp
HM596647, HM596651, HM596652, HM596658, HM596660, HM596661, HM596666, HM596688	Indonesia - Sumatra	[91]
HQ700841, HQ700842, HQ700843, HQ700844, HQ700845, HQ700846, HQ700847, HQ700848, HQ700849, HQ700850, HQ700851, HQ700852, HQ700853, HQ700854, HQ700855, HQ700856, HQ700857, HQ700858, HQ700859, HQ700860, HQ700861, HQ700862, HQ700863, HQ700864, HQ700865, HQ700866, HQ700867, HQ700868, HQ700869, HQ700870	Guam	[105]
JN857018, JN857047, JN857054, JN857063	Russia: South Siberia	[106]
JN857048, JN857049	Russia: Kalmyk Republic	[106]
JN857050, JN857051	South Korea	[106]
JN857056	Mongolia	[106]
JQ703727	Netherlands	[94]
KC896622	Burma: Rangoon	FT-DS
KF006361	Philippines	FT-DS
KF540505, KF540514, KF540515, KF540516, KF540524, KF540525, KF540532, KF540543, KF540549	Taiwan - Ami	[96]
KF540559, KF540568, KF540569, KF540581, KF540582, KF540588, KF540599, KF540601	Taiwan - Atayal	[96]
KF540615, KF540618, KF540645	Taiwan - Bunun	[96]
KF540656, KF540693	Taiwan - Hakka	[96]
KF540711, KF540714	Taiwanese Han	[96]
KF540780	Taiwan - Paiwan	[96]
KF540805, KF540812, KF540819, KF540824, KF540827, KF540832, KF540844	Taiwan - Makatao	[96]
KF540851, KF540852, KF540854, KF540859, KF540861, KF540869, KF540870, KF540876, KF540880, KF540883, KF540886, KF540887, KF540888	Taiwan - Puyuma	[96]
KF540944, KF540949, KF540950, KF540952	Taiwan - Saisiat	[96]
KF540968	Taiwan - Tao	[96]
KF541014, KF541034, KF541036	Taiwan - Tsou	[96]
NA17965, NA18115	Chinese in Denver	[86]
NA18593	Han Chinese	[86]
NA18956, NA18969, NA19563	Japan	[86]

Table S9. B4a1a sequences used in the phylogenetic reconstruction

Sequence (accession number)	Location/ group	Reference
AF346993	Korea	[100]
AF347007	Samoa	[100]
AJ842744, AJ842745, AJ842748,AJ842749	Taiwan (Ami)	[45]
AJ842746	Taiwan (Atayal)	[45]
AJ842747, AJ842751	Taiwan - Tao	[45]
AJ842750	Taiwan - Paiwan	[45]
AP008257, AP008412, AP008415, AP008521, AP008567, AP008595, AP008597, AP008640, AP008650, AP008661, AP008842, AP008889, AP008912	Japan	[72]
AP009463	Japan	[107]
AP010705, AP010757	Japan	[73]
AY195770	Asia	
AY195770	Asia	[108]
AY255133	China - Guangdong	[75]
AY289068, AY289069	Cook Islander	[76]
AY289076, AY289077, AY289080, AY289083	Coastal New Guinea	[76]
AY289093, AY289094	Samoan	[76]
AY289102	Tonga	[76]
AY519492	Tofalar, Russia: Siberia	[39]
AY519495	Tuvan; Russia: Siberia	[39]
AY963574	Melanesia Bougainville	[53]
(20 sequences not deposited in GenBank)	Maori, New Zealand	[109]
DQ272120	China	[77]
DQ372871, DQ372873	Papua New Guinea: Trobriand Islands	[78]
DQ372874, DQ372875	Micronesia: Kapingamarangi Atoll	[78]
DQ372877	Micronesia: Majuro Atoll	[78]
DQ372878, DQ372881	Vanuatu	[78]
DQ372886	Tonga	[78]
EU597505	Mongolian; China	[81]
EU597506	South China	[81]
EU597531, EU597555	Melanesian, Boungainville	[81]
FJ748745	Tibet	[82]
FJ767910, FJ767911, FJ767912	Madagascar	[110]
GQ119021, GQ119029	Philippines	[55]
GQ214523	Kiribati	[111]
GU733730, GU733732	Philippines - Mamanwa; negrito group	[85]
GU733764, GU733797	Philippines - Manobo	[85]
GU733802, GU733812, GU733824	Philippines - Surigaonon	[85]
GU810060, GU810061, GU810067, GU810068	sea nomads of	Unp

	Thailand	
HG00419, HG00452, HG00537	South Chinese	[86]
HG00599, HG00608, HG00631, HG00654, HG00729	South Chinese	[87]
HG01869, HG02072, HG02122, HG02134	Kinh in Ho Chi Minh City, Vietnam	[87]
HG02399	Chinese Dai	[87]
HM238197, HM238202, HM238207	Philippine Islanders - Ivatan	[89]
HM238212, HM238213	Orchid Islands - Yami	[89]
HM596665, HM596684, HM596686, HM596696, HM596699, HM596700, HM596704	Indonesia - Sumatra	[91]
HQ700839, HQ700840	Guam - Micronesia	[105]
HQ873489, HQ873495, HQ873566	Vietnam	[112]
HQ873496, HQ873497, HQ873498, HQ873500, HQ873501, HQ873502, HQ873503, HQ873504, HQ873505, HQ873506, HQ873507, HQ873508, HQ873509, HQ873510, HQ873511, HQ873512, HQ873513, HQ873514, HQ873515, HQ873516, HQ873517, HQ873518, HQ873519	Bismarch Archipelago	[112]
HQ873564, HQ873568, HQ873569	China	[112]
HQ873546, HQ873550, HQ873552, HQ873554	Indonesia - Ambon	[112]
HQ873538, HQ873544, HQ873545, HQ873556	Indonesia - Banjarmasin	[112]
HQ873559	Indonesia - Java	[112]
HQ873493, HQ873541, HQ873555	Indonesia - Manado	[112]
HQ873540, HQ873548, HQ873553	Indonesia - Mataran	[112]
HQ873549	Indonesia - Palangkaraya	[112]
HQ873494, HQ873551	Indonesia - Toraja	[112]
HQ873499, HQ873547	Indonesia - Ujung Padang	[112]
HQ873539	Indonesia - Waingapu	[112]
HQ873542, HQ873543	Malaysia - Kota Kinabalu	[112]
HQ873490, HQ873491, HQ873492, HQ873530, HQ873531, HQ873533, HQ873534, HQ873535	Papua New Guinea	[112]
HQ873536, HQ873537	Philippine	[112]
HQ873557, HQ873558	Taiwan - Ami	[112]
HQ873560, HQ873565	Taiwan - Siraya	[112]
HQ873561	Taiwan - Tsou	[112]
HQ873563	Taiwan - Saisiat	[112]
HQ873562	Thailand	[112]
HQ873520, HQ873521, HQ873522, HQ873523, HQ873524, HQ873525, HQ873526, HQ873527, HQ873528, HQ873529	Vanuatu	[112]
HQ873532	West New Guinea	[112]
JQ411478, JQ411479	Chinese	Unp
JQ703874	Thailand	[94]
JQ704922, JQ705700	Hawai'i	[94]
JX893364, JX893365	Maori - Ancient DNA	[113]
JX900327, JX900328, JX900329, JX900330, JX900331, JX900332, JX900333, JX900334, JX900335, JX900336,	Solomon Islands: Bellona	[114]

JX900337, JX900338, JX900339, JX900340, JX900341, JX900342, JX900343, JX900344, JX900345, JX900346, JX900347, JX900348, JX900349, JX900350, JX900351, JX900352, JX900353, JX900354, JX900355, JX900356, JX900357, JX900358, JX900359, JX900360, JX900361, JX900362, JX900363, JX900364, JX900365, JX900366, JX900367, JX900368, JX900369		
JX900370, JX900371	Solomon Islands: Choiseul	[114]
JX900372, JX900373, JX900374, JX900375, JX900376, JX900377, JX900378, JX900380, JX900381, JX900382, JX900383, JX900384, JX900385, JX900386, JX900387, JX900388, JX900389, JX900390, JX900391, JX900392, JX900393, JX900394, JX900395, JX900396, JX900397, JX900398	Solomon Islands: Gela	[114]
JX900399, JX900400, JX900401, JX900402, JX900403, JX900404, JX900405, JX900406, JX900407, JX900408, JX900409, JX900410, JX900411, JX900412, JX900413, JX900414, JX900415, JX900416, JX900417, JX900418, JX900419, JX900420, JX900422, JX900423, JX900424, JX900425, JX900426, JX900427, JX900428, JX900429, JX900689, JX900690, JX900691, JX900692, JX900693, JX900694, JX900695, JX900696, JX900697, JX900698, JX900699, JX900700, JX900701, JX900702, JX900703, JX900704, JX900705, JX900706, JX900707, JX900708, JX900709, JX900710, JX900711, JX900712, JX900713, JX900714, JX900715, JX900716, JX900717, JX900718, JX900719, JX900720, JX900721, JX900722, JX900723, JX900724, JX900725, JX900727, JX900728	Solomon Islands: Russell	[114]
JX900430, JX900431, JX900432, JX900433, JX900434, JX900435, JX900436, JX900437, JX900438, JX900439, JX900440, JX900441, JX900831, JX900832, JX900833, JX900834, JX900835, JX900836, JX900837, JX900838, JX900839, JX900840, JX900841, JX900842, JX900843, JX900844, JX900845, JX900847, JX900848, JX900849, JX900850, JX900851, JX900852, JX900853, JX900854, JX900855, JX900856, JX900857, JX900858, JX900859, JX900860, JX900861, JX900862	Solomon Islands: Vella Lavella	[114]
JX900442, JX900443, JX900444, JX900445, JX900446, JX900447, JX900448, JX900449, JX900450, JX900451, JX900452, JX900453, JX900454, JX900455, JX900456, JX900457, JX900458, JX900459, JX900460, JX900461, JX900462, JX900463, JX900464, JX900465, JX900466, JX900467, JX900468, JX900469, JX900470, JX900471, JX900472, JX900754, JX900755, JX900756, JX900758, JX900759, JX900760, JX900761, JX900762, JX900763, JX900764, JX900765, JX900766, JX900767, JX900768	Solomon Islands: Isabel	[114]
JX900474, JX900475, JX900476, JX900477, JX900478, JX900479, JX900480, JX900483, JX900484, JX900485, JX900486	Solomon Islands: Makira	[114]
JX900487, JX900488, JX900489, JX900490, JX900491,	Solomon Islands:	[114]

JX900492, JX900493, JX900494, JX900495, JX900497, JX900498, JX900499, JX900500, JX900501, JX900502, JX900503, JX900504, JX900505, JX900506, JX900507, JX900508, JX900509, JX900511, JX900512, JX900513, JX900514, JX900515, JX900516, JX900517, JX900518, JX900519, JX900520, JX900521, JX900522, JX900523, JX900524, JX900525, JX900526, JX900527	Shortlands	
JX900528, JX900529, JX900530, JX900531, JX900532, JX900533, JX900534, JX900535, JX900536, JX900537, JX900538, JX900539, JX900540, JX900541, JX900542, JX900543, JX900544, JX900545, JX900546, JX900547, JX900548, JX900550, JX900551, JX900552, JX900553, JX900554, JX900555, JX900556, JX900557, JX900558, JX900559, JX900560, JX900561, JX900562, JX900563, JX900564, JX900565, JX900566, JX900567, JX900568, JX900569, JX900570, JX900572, JX900573, JX900574, JX900846	Solomon Islands: Malaita	[114]
JX900575, JX900576, JX900577, JX900578, JX900579, JX900580, JX900581, JX900582, JX900583, JX900584, JX900585, JX900586, JX900587, JX900588, JX900589, JX900590, JX900591, JX900592, JX900593, JX900594, JX900595, JX900596, JX900597, JX900598	Solomon Islands: Ontong Java	[114]
JX900599, JX900600, JX900601, JX900602, JX900603, JX900604, JX900605, JX900606, JX900607, JX900608, JX900609, JX900610, JX900611, JX900612, JX900613, JX900614, JX900615, JX900616, JX900617, JX900618, JX900619, JX900620, JX900621, JX900622, JX900623, JX900624, JX900625, JX900626, JX900627, JX900628, JX900629, JX900630, JX900631, JX900632, JX900633, JX900634, JX900635, JX900636, JX900637, JX900638, JX900639, JX900640, JX900641, JX900642, JX900643, JX900644, JX900645, JX900646, JX900647, JX900648, JX900649, JX900650	Solomon Islands: Ranongga	[114]
JX900651, JX900652, JX900653, JX900654, JX900655, JX900656, JX900657, JX900658, JX900659, JX900660, JX900661, JX900662, JX900663, JX900664, JX900665, JX900666, JX900667, JX900668, JX900669, JX900670, JX900671, JX900672, JX900673, JX900674, JX900675, JX900676, JX900677, JX900678, JX900679, JX900680, JX900681, JX900682, JX900683, JX900684, JX900685, JX900686, JX900687, JX900688	Solomon Islands: Rennell	[114]
JX900729, JX900730, JX900731, JX900732, JX900733, JX900735, JX900736, JX900737, JX900738, JX900739, JX900740, JX900741, JX900742, JX900743, JX900744, JX900745, JX900746, JX900747, JX900748, JX900749, JX900750, JX900751, JX900752, JX900753, JX900811, JX900812, JX900813, JX900814, JX900815, JX900816, JX900817, JX900818, JX900819, JX900820, JX900821, JX900822, JX900823, JX900824, JX900825, JX900826, JX900827, JX900828, JX900829, JX900830	Solomon Islands: Tikopia	[114]
JX900769, JX900770, JX900771, JX900772, JX900773,	Solomon Islands: Savo	[114]

JX900774, JX900775, JX900776, JX900777, JX900778, JX900779, JX900780, JX900781, JX900782, JX900783, JX900784, JX900785, JX900786, JX900787, JX900788, JX900789, JX900790, JX900791, JX900792, JX900793, JX900794, JX900795, JX900796, JX900797, JX900798, JX900799, JX900801, JX900802, JX900803, JX900804, JX900805		
JX900806, JX900807, JX900808, JX900809, JX900810	Solomon Islands: Santa Cruz	[114]
KC993914, KC993917, KC993918, KC993922	Philippines - Abaknon	[95]
KC994020, KC994021, KC994024, KC994027, KC994029	Philippines - Ibaloi	[95]
KC994045, KC994047, KC994049, KC994050, KC994053	Philippines - Ifugao	[95]
KC994063, KC994064, KC994073, KC994074, KC994080	Philippines - Ivatan	[95]
KC994091, KC994097, KC994101, KC994104, KC994105, KC994107, KC994109	Philippines - Kalangoya	[95]
KC994114, KC994115, KC994119, KC994132, KC994136	Philippines - Kankanaey	[95]
KC994155, KC994161	Philippines - Maranao	[95]
KF540509, KF540512, KF540513, KF540517, KF540521, KF540522, KF540528, KF540534, KF540538, KF540551, KF540554	Taiwan - Ami	[96]
KF540572, KF540590	Taiwan - Atayal	[96]
KF540665, KF540673, KF540685, KF540688, KF540689, KF540690, KF540699	Taiwan - Hakka	[96]
KF540706, KF540707, KF540708, KF540716	Taiwanese Han	[96]
KF540756, KF540761, KF540763, KF540764, KF540765, KF540767, KF540773, KF540774, KF540777, KF540779, KF540784, KF540787, KF540792, KF540796, KF540797, KF540798	Taiwan - Paiwan	[96]
KF540814, KF540829, KF540836, KF540843	Taiwan - Makatao	[96]
KF540894, KF540897, KF540905, KF540913	Taiwan - Rukai	[96]
KF540954	Taiwan - Saisiat	[96]
KF540967, KF540983, KF540987, KF540991, KF540997, KF540998, KF541000, KF541001	Taiwan - Tao	[96]
KF541020, KF541032, KF541042, KF541047	Taiwan - Tsou	[96]
NA17990, NA18109, NA18141	Chinese in Dever, USA	[86]
NA18528, NA18537, NA18541, NA18567, NA18614, NA18617, NA18770, NA18794	China-Beijing	[86]
NA18948, NA18975, NA19551	Japan	[86]
SSM091	Malaysia	[99]

Table S10. Sequences used in the ancient DNA fossil calibration with BEAST

Sequence (accession number)	Reference
AF346995	[100]
AP008482	[72]
AY714005	[115]
AY882379	[116]
DQ200802	[117]
DQ304925	[118]
DQ304954	[118]
DQ305010	[118]
DQ305018	[118]
DQ341063	[119]
DQ341074	[119]
DQ341075	[119]
DQ341080	[119]
DQ341081	[119]
DQ341089	[119]
EF093542	[101]
EF093547	[101]
EF093548	[101]

EF185794	[101]
EF185804	[101]
EF222234	[120]
EF556173	[121]
EU092660	[122]
EU092661	[122]
EU092678	[122]
EU092686	[122]
EU092699	[122]
EU092708	[122]
EU092712	[122]
EU092715	[122]
EU092717	[122]
EU092724	[122]
EU092736	[122]
EU092740	[122]
EU092748	[122]
EU092752	[122]
EU092766	[122]
EU092770	[122]
EU092773	[122]
EU092774	[122]
EU092776	[122]
EU092792	[122]
EU092802	[122]
EU092817	[122]
EU092818	[122]
EU092822	[122]
EU092824	[122]
EU092831	[122]
EU092837	[122]
EU092838	[122]
EU092848	[122]
EU092851	[122]
EU092870	[122]
EU092878	[122]
EU092886	[122]
EU092888	[122]
EU092891	[122]
EU092902	[122]
EU092913	[122]
EU092915	[122]
EU092921	[122]

EU092923	[122]
EU092934	[122]
EU092935	[122]
EU092941	[122]
EU092942	[122]
EU092949	[122]
EU092964	[122]
EU273489	[123]
EU273493	[123]
EU273499	[123]
EU330890	FT-DS
EU439939	[124]
EU597502	[81]
EU597570	[81]
EU935440	[125]
FJ004823	[126]
FJ383248	[102]
FJ383712	[127]
FJ460520	[128]
FJ460531	[128]
FJ625856	[129]
FJ951545	[130]
HM185239	[131]
HM596698	[91]
HM596745	FT - DS
HM771114	[132]
HM771162	[132]
HM771166	[132]
HM771184	[132]
HM771203	[132]
HM771211	[132]
HM771233	[132]
HQ012103	[133]
HQ873562	[112]
JN655776	[134]
JN655780	[134]
JN655784	[134]
JN655785	[134]
JN655786	[134]
JN655787	[134]
JN655788	[134]
JN655794	[134]
JN655798	[134]

JN655803	[134]
JN655813	[134]
JN655815	[134]
JN655825	[134]
JN655830	[134]
JN655837	[134]
JQ044811	[135]
JQ044816	[135]
JQ044829	[135]
JQ044831	[135]
JQ044834	[135]
JQ044858	[135]
JQ044882	[135]
JQ044907	[135]
JQ044922	[135]
JQ044936	[135]
JQ045008	[135]
JQ045026	[135]
JQ045062	[135]
JQ045080	[135]
JQ045092	[135]
JQ045101	[135]
JQ701834	[94]
JQ702441	[94]
JQ702617	[94]
JQ702659	[94]
JQ702802	[94]
JQ703793	[94]
JQ704286	[94]
JQ704875	[94]
JQ704919	[94]
JQ705000	[94]
JQ705310	[94]
JQ705673	[94]
KC417443[a]	[136]
KC521454[b]	[137]
KC911536	[138]
KF540505[c]	[96]

[a] Ancient sample: 39475 years
[b] Ancient sample: 8180 years
[c] Ancient sample: 7900 years

Table S11. List of founders under the *f1* and *f2* criteria [139] including the effective number of samples for each founder, the age estimate and its standard error. Fifty networks displayed relevant founders for ISEA. The positions of the variants at the root for each network against the rCRS (less 16,000) are indicated, but note that the classification of the lineages was based in many cases on additional coding-region typing.

Network (root variants against rCRS)	Variants in network from root to founder	*f1* criterion			*f2* criterion		
		n	Age estimate (years)	Standard error	n	Age estimate (years)	Standard error
**A4 (223, 290, 319, 362)**	**root**	1	0	0	1	0	0
**B4a (189, 217, 261)**	**root**	142	8691	3486	172	11344	3481
	**168 311**	1	0	0			
	**242**	1	0	0	1	0	0
	**92**	10	15009	10678			
	**286**	1	0	0			
	**178**	2	0	0			
	**278**	1	0	0			
	**223**	16	5212	2331			
	**311**	2	0	0	3	5559	5559
	**324**	4	0	0	4	0	0
	**93**	1	0	0	1	0	0
	**129**	2	0	0	2	0	0
**B4b (136,189,217)**	**root**	64	4951	1669	79	7389	2728
	**92**	1	0	0			
	**217**	1	0	0			
	**300**	11	0	0			
	**86**	2	0	0	2	0	0
	**261**	2	0	0			
**B4 (189, 217)**	**root**	3	16677	9628	8	37523	18411
	**311 92 274 140 335**	1	0	0			
	**92 274 140 335**	1	0	0			
	**129 274 140 335**	1	0	0			
	**136 274 140 335**	1	0	0			
	**311 274 140 335**	7	2382	2382			
	**274 140 335**	54	1853	874	65	5645	2578
	**184A 235 147**	11	15161	6780	11	15161	6780
	**274 335**	1	0	0			
	**140 335**	2	8339	8339			
	**362 140**	1	0	0			
	**274 140**	6	2779	2779	6	2779	2779
	**235 147**	13	8980	3394	13	8980	3394
	**235**	5	0	0	3	22236	13617
	**147**	9	7412	3706	9	7412	3706
	**140**				1	0	0
**B5 (140,189)**	**root**	5	6671	4717	5	6671	4717
	**129 111 234 243**	10	15009	13445			
	**111 234 243**	2	0	0	12	26405	17852
	**309 243**	1	0	0			
	**218 243**	3	0	0			
	**355 243**	2	0	0	2	0	0
	**234 243**	1	0	0	1	0	0
	**145 266A**	2	0	0			

	**260 266A**	1	0	0	1	0	0
	**266G 266A**	1	0	0	1	0	0
	**140 266A**	1	0	0	1	0	0
	**261 266A**	5	6671	4717	5	6671	4717
	**243**	56	3276	1298	60	4169	1497
	**266A**	46	3988	1202	48	5212	1514
**B (189)**	**root**	20	10840	6295	21	13500	6202
	**129**	1	0	0	1	0	0
	**93**	1	0	0			
	**51**	2	0	0	2	0	0
**C (223, 298, 327)**	**root**	5	6671	4717	6	8339	4814
	**298**	1	0	0	1	0	0
	**51**	1	0	0			
**D2 (129, 223, 271, 362)**	**129**	1	0	0	1	0	0
**D4b2b2b (172, 362)**	**root**	1	0	0	1	0	0
**D4e1 (092, 223, 362)**	**root**	1	0	0	1	0	0
**D with 274 (223, 274, 362)**	**root**	5	86720	33354	10	71711	20628
	**278**	1	0	0			
	**129**	4	4169	4169			
	**192**	2	0	0	2	0	0
	**311**	1	0	0	1	0	0
**D4i (223, 294, 362)**	**root**	1	0	0	1	0	0
**D4j2 (223, 291, 362)**	**root**	2	0	0	2	0	0
**D4 (223, 362)**	**root**	22	7580	3556	31	13987	5942
	**311 189**	1	0	0			
	**261**	7	0	0			
	**209**	1	0	0			
	**355**	1	0	0	1	0	0
	**301**	2	0	0	2	0	0
	**234**	1	0	0	1	0	0
	**286**	1	0	0			
	**311**	2	0	0	2	0	0
	**93**	2	0	0	2	0	0
	**189**				1	0	0
**D5 (189, 223, 362)**	**root**	6	2779	2779	6	2779	2779
	**92 148**	16	3127	1805	16	3127	1805
	**311**	2	0	0	2	0	0
	**172**	1	0	0	1	0	0
	**148**	8	4169	2948	8	4169	2948
**E (223, 362, 390)**	**root**	63	20383	12593	107	24782	10382
	**185 51**	10	3335	3335			
	**51**	34	12753	5678			
	**291**	179	8665	1770	179	8665	1770
**F1 (129, 304)**	**266**	2	16677	11792	2	16677	11792
**F1a (129, 172, 304)**	**root**	103	14572	7792	105	14612	7650
	**362 294**	91	4032	1533	91	4032	1533
	**189 129**	1	0	0	1	0	0
	**301**	2	0	0			
	**362**	7	9530	6739	7	9530	6739
	**294**	1	0	0	1	0	0

	**295**	2	16677	11792	2	16677	11792
	**129**	1	0	0	1	0	0
**F1a1a (129, 162, 172, 304)**	**root**	7	4765	3369	9	11118	5860
	**189**	2	0	0			
	**399**	2	8339	8339	2	8339	8339
**F1a1a1 (108, 129, 162, 172, 304)**	**root**	44	5306	1857	45	5559	1853
	**398**	3	16677	12430	3	16677	12430
	**391**	2	0	0	2	0	0
	**293**	1	0	0			
	**304**	1	0	0	1	0	0
**F3 (298, 362)**	**root**	1	0	0	1	0	0
	**311 93 265 220C**	2	0	0	2	0	0
	**93 265 220C**	2	0	0			
	**93 260 355**	1	0	0	1	0	0
	**265 220C**	35	22395	10918	37	22086	10367
	**93 220C**	5	0	0			
	**220C**	9	31501	19871	14	26207	14095
	**355**	1	0	0	1	0	0
**F4b (218, 304, 311)**	**root**	5	13342	9434	5	13342	9434
**G2a (223, 227, 278, 362)**	**root**	1	0	0	1	0	0
	**227 189**	1	0	0	1	0	0
**M10a1 (129, 223, 311)**	**129 93 193**	2	66708	31200	2	66708	31200
**M11a2 (173, 223)**	**root**	5	56702	29268	5	56702	29268
**M12 (223, 234, 290)**	**root**	1	0	0	1	0	0
	**362 93 311 129 362**	1	0	0			
	**249 189 172**	1	0	0			
	**311 129 362**	4	0	0			
	**189 172**	1	0	0	2	25016	14443
	**129 172**	1	0	0			
	**261**	2	16677	11792			
	**129 362**				5	40025	22125
	**172**				1	0	0
	**261**				2	16677	11792
**M13b1 (129, 223, 263)**	**root**	18	50031	16522	18	50031	16522
**M26 (214A, 223, 256, 278)**	**root**	6	11118	8790	6	11118	8790
**M71 (223, 271)**	**root**	1	0	0	7	54796	16333
	**140 129**	2	25016	14443	2	25016	14443
	**129**	4	41693	17689			
	**269**	9	9265	9265	9	9265	9265
	**311**	2	8339	8339			
**M74 (223, 311, 362)**	**root**	41	13423	5621	41	13423	5621
**M76 (189, 193C, 362)**	**124**	1	0	0	1	0	0
**M7a (209, 223)**	**root**	1	0	0	1	0	0
**M7b (129, 223, 297)**	**root**	1	0	0	2	16677	11792
	**189 129**	1	0	0	1	0	0
	**191**	1	0	0			
	**189**	5	3335	3335	5	3335	3335
	**129**	1	0	0	1	0	0
**M7b1 (129, 192,**	**root**	3	16677	9628	13	24374	11546

**223, 297)**	**129**	2	25016	18645			
	**126**	8	6254	4661			
	**189**	12	8339	5896	12	8339	5896
**M7b3 (086, 129, 297)**	**root**	26	6414	3009	26	6414	3009
**M7c3 (223, 295)**	**root**	1	0	0	3	22236	15723
	**274 362**	1	0	0			
	**93 362**	6	19456	14443			
	**311 362**	4	8339	8339			
	**168 362**	9	27795	11572	9	27795	11572
	**295 319**	9	29648	18714			
	**311**	2	0	0			
	**86**	1	0	0	1	0	0
	**362**	155	5164	1282	166	5626	1714
	**319**				9	46325	25067
**M7 (223)**	**root**	1	0	0	2	41693	18645
	**129**	1	0	0			
	**362**	9	0	0	9	0	0
	**223**	1	0	0	1	0	0
**M8a (223, 298, 319)**	**root**	1	0	0	1	0	0
**M9 (223, 234, 362)**	**158**	1	0	0	1	0	0
**M (223)**	**root**	31	42499	6784	78	58156	6569
	**278 172 189 140**	2	0	0	2	0	0
	**381 344 304**	1	0	0			
	**319 311 278 243**	1	0	0			
	**181 304 291 145**	3	22236	17579			
	**192 304 291 145**	2	0	0	2	0	0
	**209 325**	7	23824	15440			
	**86 272**	7	42884	21309			
	**209 129 272**	12	4169	3108			
	**311 249**	1	0	0			
	**304 291 145**	7	2382	2382	10	13342	7458
	**311 278**	1	0	0			
	**140**	4	29185	11031	4	29185	11031
	**325**	1	0	0			
	**305**	9	72267	29240			
	**299**	1	0	0			
	**295**	1	0	0			
	**291**	1	0	0			
	**287**	3	22236	15723			
	**284**	5	20012	14151			
	**272**	2	58370	22062	21	46854	16449
	**259**	4	83385	29481	4	83385	29481
	**233**	7	83385	23464			
	**219**	3	0	0	3	0	0
	**209**	1	0	0			
	**193**	1	0	0	1	0	0
	**172**	2	16677	11792	2	16677	11792
	**166**	1	0	0			
	**148**	3	38913	18437			
	**124**	1	0	0	1	0	0
	**147**	3	33354	17579			
	**278**	20	29185	8544	21	28589	8176

	**184A**	6	30574	13330	6	30574	13330
	**311**	7	0	0	7	0	0
	**362**	3	11118	11118	3	11118	11118
	**129**	2	0	0			
	**234**	4	54200	23951	4	54200	23951
	**344 304**				1	0	0
**N9a (223, 257A, 261)**	**292**	9	37060	17383	15	28907	12380
	**189 292**	6	0	0			
**N9b (189, 223)**	**root**	2	33354	16677	2	33354	16677
**N(223)**	**root**	9	40766	16365	17	43164	12095
	**357 311 343 274 263**	12	19456	13758			
	**224 319 274 263**	1	0	0			
	**249 168**	5	20012	12480			
	**111 172**	5	73379	27906			
	**291**	1	0	0			
	**213**	2	0	0			
	**311 343 274 263**				12	19456	13758
	**274 263**				1	0	0
	**172**				5	90056	32509
**R9 with 189 (189, 304)**	**root**	2	16677	11792	3	22236	11118
	**284**	1	0	0	1	0	0
	**311**	1	0	0			
**R9b (304, 309, 390)**	**192 309 288**	1	0	0			
	**172 390**	1	0	0			
	**192 288**	10	16677	10808	10	16677	10808
	**309**	1	0	0	1	0	0
	**288**	3	0	0	4	12508	7221
	**390**				1	0	0
**R9c (157, 304)**	**root**	5	10006	10006	6	11118	8790
	**311 335 256**	4	12508	12508			
	**335 256**	50	7004	2187	54	8647	2547
	**256**	1	0	0			
**R9 (304)**	**root**	5	23348	12026	13	43617	14739
	**233**	5	53366	29077			
	**362**	2	0	0	2	0	0
	**209**	3	0	0			
**R (0)**	**root**	7	33354	11175	14	35736	10521
	**301 390 304 249 288**	3	0	0	3	0	0
	**390 304 249 288**	9	0	0			
	**304 249 288**	5	13342	8170	14	15486	11111
	**249 288**	4	20846	11031			
	**355**	1	0	0			
	**288**	2	58370	27656	6	44472	16207
	**192**	1	0	0			
	**172**	1	0	0	1	0	0
	**256**	5	3335	3335			
	**189**	11	1516	1516	11	1516	1516
**Y (126, 231)**	**root**				1	0	0
	**209**	1	0	0			
	**189**	1	0	0	1	0	0
	**311**	58	5463	2620	58	5463	2620
**Z (185, 223, 260, 298)**	**root**	1	0	0	3	16677	12430
	**185**	6	13898	13898	6	13898	13898
	**129**	2	8339	8339			

Table S12. Relevant age estimates of three clades for the phylogeographic parameters defined in the main text using the traditional 95% confidence interval (CI) and the expanded 95% CI calculated as in Mellars et al. [140].

	Clade	Age estimate (years)	95% confidence interval	Expanded 95% confidence interval
**Founder age estimate of putative migration Taiwan to ISEA**	**B4a1a**	7270	[5210; 9370]	[4920;9660]
	**E**	8770	[5980; 11600]	[5670;11920]
	**M7c3c**	4460	[3220; 5720]	[3040;5900]
**Founder age estimate of putative migration Taiwan and Philippines to rest of ISEA**	**B4a1a**	8520	[4770; 12340]	[4550;12580]
	**E**	6400	[4780; 8030]	[4500;8320]
	**M7c3c**	4200	[2520; 5890]	[2400;6020]
**Age estimate of clade**	**B4a1**	14700	[11020; 18460]	[10350;19150]
	**M9**	39160	[26870; 51960]	[25350;53620]
	**M7c3**	11830	[3880; 20220]	[3680;18270]
	**B4a1a**	9940	[5530; 14460]	[5270;14740]
	**E**	23950	[14470; 33840]	[13740;34630]
	**M7c3c**	5230	[4000; 6470]	[3760;6720]

Table S13. Increment period, peak of increment and ratio of increment in the Bayesian skyline plots (BSPs) for mtDNA haplogroups B4a1a, E and M7c3c in ISEA and Taiwan

**Clade**	**Location**	**Increment period**	**Ratio of increment**	**Peak**
B4a1a	*ISEA*	3.5-10.2 ka	21x	6.7 ka
	Taiwan	0.4-9.3 ka	85x	6.7 ka; 1.5 ka
E	*ISEA*	3.8-7.7 ka	11.5x	6.1 ka
	Taiwan	3.1-7.4 ka	8.9x	5.2 ka
M7c3c	*ISEA*	2.2-5.2 ka	7.6x	4 ka
	Taiwan	3.6-7.6 ka	2.9x	5.2 ka

References

1. Jin HJ, Tyler-Smith C, Kim W (2009) The peopling of Korea revealed by analyses of mitochondrial DNA and Y-chromosomal markers. PLoS ONE 4: e0004210.
2. Yao YG, Nie L, Harpending H, Fu YX, Yuan ZG, Zhang YP (2002) Genetic relationship of Chinese ethnic populations revealed by mtDNA sequence diversity. American Journal of Physical Anthropology 118: 63-76.
3. Li H, Cai X, Winograd-Cort ER, Wen B, Cheng X, Qin Z, Liu W, Liu Y, Pan S, Qian J, Tan CC, Jin L (2007) Mitochondrial DNA diversity and population differentiation in southern East Asia. American Journal of Physical Anthropology 134: 481-488.
4. Gan RJ, Pan SL, Mustavich LF, Qin ZD, Cai XY, Qian J, Liu CW, Peng JH, Li SL, Xu JS, Jin L, Li H (2008) Pinghua population as an exception of Han Chinese's coherent genetic structure. Journal of Human Genetics 53: 303-313.
5. Wen B, Li H, Gao S, Mao X, Gao Y, Li F, Zhang F, He Y, Dong Y, Zhang Y, Huang W, Jin J, Xiao C, Lu D, Chakraborty R, Su B, Deka R, Jin L (2005) Genetic structure of Hmong-Mien speaking populations in East Asia as revealed by mtDNA lineages. Molecular Biology and Evolution 22: 725-734.
6. Wen B, Hui L, Lu D, Song X, Zhang F, He Y, Li F, Gao Y, Mao X, Zhang L, Qian J, Tan J, Jin J, Huang W, Deka R, Su B, Chakraborty R, Jin L (2004) Genetic evidence supports demic diffusion of Han culture. Nature 431: 302-305.
7. Liu C, Wang SY, Zhao M, Xu ZY, Hu YH, Chen F, Zhang RZ, Gao GF, Yu YS, Kong QP (2011) Mitochondrial DNA polymorphisms in Gelao ethnic group residing in Southwest China. Forensic Science International: Genetics 5: e4-e10.
8. Li B, Zhong F, Yi H, Wang X, Li L, Wang L, Qi X, Wu L (2007) Genetic Polymorphism of Mitochondrial DNA in Dong, Gelao, Tujia, and Yi Ethnic Populations from Guizhou, China. Journal of Genetics and Genomics 34: 800-811.
9. Tajima A, Hayami M, Tokunaga K, Juji T, Matsuo M, Marzuki S, Omoto K, Horai S (2004) Genetic origins of the Ainu inferred from combined DNA analyses of maternal and paternal lineages. Journal of Human Genetics 49: 187-193.
10. Wen B, Xie X, Gao S, Li H, Shi H, Song X, Qian T, Xiao C, Jin J, Su B, Lu D, Chakraborty R, Jin L (2004) Analyses of Genetic Structure of Tibeto-Burman Populations Reveals Sex-Biased Admixture in Southern Tibeto-Burmans. American Journal of Human Genetics 74: 856-865.
11. Zhao M, Kong QP, Wang HW, Peng MS, Xie XD, Wang WZ, Jiayang, Duan JG, Cai MC, Zhao SN, Cidanpingcuo, Tu YQ, Wu SF, Yao YG, Bandelt H-J, Zhang YP (2009) Mitochondrial genome evidence reveals successful Late Paleolithic settlement on the Tibetan Plateau. Proceedings of the National Academy of Sciences of the United States of America 106: 21230-21235.
12. Nishimaki Y, Sato K, Fang L, Ma M, Hasekura H, Boettcher B (1999) Sequence polymorphism in the mtDNA HV1 region in Japanese and Chinese. Legal Medicine 1: 238-249.
13. Cheng B, Tang W, He L, Dong Y, Lu J, Lei Y, Yu H, Zhang J, Xiao C (2008) Genetic imprint of the Mongol: Signal from phylogeographic analysis of mitochondrial DNA. Journal of Human Genetics 53: 905-913.
14. Yao YG, Zhang YP (2002) Phylogeographic analysis of mtDNA variation in four ethnic populations from Yunnan Province: New data and a reappraisal. Journal of Human Genetics 47: 311-318.
15. Qian YP, Chu ZT, Dai Q, Wei CD, Chu JY, Tajima A, Horai S (2001) Mitochondrial DNA polymorphisms in Yunnan nationalities in China. Journal of Human Genetics 46: 211-220.

16. Yao YG, Kong QP, Bandelt H-J, Kivisild T, Zhang YP (2002) Phylogeographic differentiation of mitochondrial DNA in Han Chinese. American Journal of Human Genetics 70: 635-651.
17. Yao YG, Lü XM, Luo HR, Li WH, Zhang YP (2000) Gene admixture in the Silk Road region of China: Evidence from mtDNA and melanocortin 1 receptor polymorphism. Genes and Genetic Systems 75: 173-178.
18. Yao YG, Kong QP, Wang CY, Zhu CL, Zhang YP (2004) Different matrilineal contributions to genetic structure of ethnic groups in the Silk Road region in China. Molecular Biology and Evolution 21: 2265-2280.
19. Oota H, Kitano T, Jin F, Yuasa I, Wang L, Ueda S, Saitou N, Stoneking M (2002) Extreme mtDNA homogeneity in ontinental Asian populations. American Journal of Physical Anthropology 118: 146-153.
20. Zhang Y, Xu Q, Cui H, Cui Y, Lin H, Kim K, Lee J (2005) Haplotype diversity in mitochondrial DNA hypervariable region I, II and III in a Korean ethnic group from northeast China. Forensic Science International 151: 299-301.
21. Zhang YJ, Xu QS, Zheng ZJ, Lin HY, Lee JB (2005) Haplotype diversity in mitochondrial DNA hypervariable region I, II and III in northeast China Han. Forensic Science International 149: 267-269.
22. Kivisild T, Tolk HV, Parik J, Wang Y, Papiha SS, Bandelt H-J, Villems R (2002) The emerging limbs and twigs of the East Asian mtDNA tree. Molecular Biology and Evolution 19: 1737-1751.
23. Wang WZ, Wang CY, Cheng YT, Xu AL, Zhu CL, Wu SF, Kong QP, Zhang YP (2010) Tracing the origins of Hakka and Chaoshanese by mitochondrial DNA analysis. American Journal of Physical Anthropology 141: 124-130.
24. Wang Q, Wang P, Li S, Xiao X, Jia X, Guo X, Kong QP, Yao YG, Zhang Q (2010) Mitochondrial DNA haplogroup distribution in Chaoshanese with and without myopia. Molecular Vision 16: 303-309.
25. Betty DJ, Chin-Atkins AN, Croft L, Sraml M, Easteal S (1996) Multiple independent origins of the COII/tRNA(Lys) intergenic 9-bp mtDNA deletion in aboriginal Australians [4]. American Journal of Human Genetics 58: 428-433.
26. Irwin JA, Saunier JL, Beh P, Strouss KM, Paintner CD, Parsons TJ (2009) Mitochondrial DNA control region variation in a population sample from Hong Kong, China. Forensic Science International: Genetics 3: e119-e125.
27. Kolman CJ, Sambuughin N, Bermingham E (1996) Mitochondrial DNA analysis of mongolian populations and implications for the origin of new world founders. Genetics 142: 1321-1334.
28. Comas D, Calafell F, Mateu E, Pérez-Lezaun A, Bosch E, Martínez-Arias R, Clarimon J, Facchini F, Fiori G, Luiselli D, Pettener D, Bertranpetit J (1998) Trading genes along the silk road: mtDNA sequences and the origin of central Asian populations. American Journal of Human Genetics 63: 1824-1838.
29. Pakendorf B, Novgorodov IN, Osakovskij VL, Danilova AP, Protod'jakonov AP, Stoneking M (2006) Investigating the effects of prehistoric migrations in Siberia: Genetic variation and the origins of Yakuts. Human Genetics 120: 334-353.
30. Horai S, Murayama K, Hayasaka K, Matsubayashi S, Hattori Y, Fucharoen G, Harihara S, Park KS, Omoto K, Pan IH (1996) mtDNA polymorphism in East Asian populations, with special reference to the peopling of Japan. American Journal of Human Genetics 59: 579-590.
31. Imaizumi K, Parsons TJ, Yoshino M, Holland MM (2002) A new database of mitochondrial DNA hypervariable regions I and II sequences from 162 Japanese individuals. International Journal of Legal Medicine 116: 68-73.

32. Nagai A, Nakamura I, Shiraki F, Bunai Y, Ohya I (2003) Sequence polymorphism of mitochondrial DNA in Japanese individuals from Gifu Prefecture. Legal Medicine 5: S210-S213.
33. Nohira C, Maruyama S, Minaguchi K (2010) Phylogenetic classification of Japanese mtDNA assisted by complete mitochondrial DNA sequences. International Journal of Legal Medicine 124: 7-12.
34. Seo Y, Stradmann-Bellinghausen B, Rittner C, Takahama K, Schneider PM (1998) Sequence polymorphism of mitochondrial DNA control region in Japanese. Forensic Science International 97: 155-164.
35. Torroni A, Schurr TG, Cabell MF, Brown MD, Neel JV, Larsen M, Smith DG, Vullo CM, Wallace DC (1993) Asian affinities and continental radiation of the four founding native American mtDNAs. American Journal of Human Genetics 53: 563-590.
36. Pfeiffer H, Steighner R, Fisher R, Mörnstad H, Yoon CL, Holland MM (1998) Mitochondrial DNA extraction and typing from isolated dentin- experimental evaluation in a Korean population. International Journal of Legal Medicine 111: 309-313.
37. Jin HJ, Kwak KD, Hong SB, Shin DJ, Han MS, Tyler-Smith C, Kim W (2006) Forensic genetic analysis of mitochondrial DNA hypervariable region I/II sequences: An expanded Korean population database. Forensic Science International 158: 125-130.
38. Derenko MV, Malyarchuk BA, Dambueva IK, Zakharov IA (2003) Structure and diversity of the mitochondrial gene pools of south Siberians. Doklady biological sciences : proceedings of the Academy of Sciences of the USSR, Biological sciences sections / translated from Russian 393: 557-561.
39. Starikovskaya EB, Sukernik RI, Derbeneva OA, Volodko NV, Ruiz-Pesini E, Torroni A, Brown MD, Lott MT, Hosseini SH, Huoponen K, Wallace DC (2005) Mitochondrial DNA diversity in indigenous populations of the southern extent of Siberia, and the origins of Native American haplogroups. Annals of Human Genetics 69: 67-89.
40. Pakendorf B, Wiebe V, Tarskaia LA, Spitsyn VA, Soodyall H, Rodewald A, Stoneking M (2003) Mitochondrial DNA evidence for admixed origins of Central Siberian populations. American Journal of Physical Anthropology 120: 211-224.
41. Schurr TG, Sukernik RI, Starikovskaya YB, Wallace DC (1999) Mitochondrial DNA variation in Koryaks and Itel'men: Population replacement in the Okhotsk Sea-Bering sea region during the neolithic. American Journal of Physical Anthropology 108: 1-39.
42. Shields GF, Schmiechen AM, Frazier BL, Redd A, Voevoda MI, Reed JK, Ward RH (1993) mtDNA sequences suggest a recent evolutionary divergence for Beringian and Northern North American populations. American Journal of Human Genetics 53: 549-562.
43. Starikovskaya YB, Sukernik RI, Schurr TG, Kogelnik AM, Wallace DC (1998) mtDNA diversity in Chukchi and Siberian Eskimos: Implications for the genetic history of ancient Beringia and the peopling of the New World. American Journal of Human Genetics 63: 1473-1491.
44. Tajima A, Sun CS, Pan IH, Ishida T, Saitou N, Horai S (2003) Mitochondrial DNA polymorphisms in nine aboriginal groups of Taiwan: Implications for the population history of aboriginal Taiwanese. Human Genetics 113: 24-33.
45. Trejaut JA, Kivisild T, Jun HL, Chien LL, Chun LH, Chia JH, Zheng YL, Lin M (2005) Traces of archaic mitochondrial lineages persist in Austronesian-speaking Formosan populations. PLoS Biology 3: e376.
46. Hill C, Soares P, Mormina M, Macaulay V, Clarke D, Blumbach PB, Vizuete-Forster M, Forster P, Bulbeck D, Oppenheimer S, Richards M (2007) A mitochondrial stratigraphy for Island Southeast Asia. American Journal of Human Genetics 80: 29-43.
47. Fucharoen G, Fucharoen S, Horai S (2001) Mitochondrial DNA polymorphisms in Thailand. Journal of Human Genetics 46: 115-125.

48. Zimmermann B, Bodner M, Amory S, Fendt L, Röck A, Horst D, Horst B, Sanguansermsri T, Parson W, Brandstätter A (2009) Forensic and phylogeographic characterization of mtDNA lineages from northern Thailand (Chiang Mai). International Journal of Legal Medicine 123: 495-501.
49. Oota H, Pakendorf B, Weiss G, Von Haeseler A, Pookajorn S, Settheetham-Ishida W, Tiwawech D, Ishida T, Stoneking M (2005) Recent origin and cultural reversion of a hunter-gatherer group. PLoS Biology 3: 0536-0542.
50. Irwin JA, Saunier JL, Strouss KM, Diegoli TM, Sturk KA, O'Callaghan JE, Paintner CD, Hohoff C, Brinkmann B, Parsons TJ (2008) Mitochondrial control region sequences from a Vietnamese population sample. International Journal of Legal Medicine 122: 257-259.
51. Mona S, Grunz KE, Brauer S, Pakendorf B, Castr L, Sudoyo H, Marzuki S, Barnes RH, Schmidtke J, Stoneking M, Kayser M (2009) Genetic admixture history of eastern indonesia as revealed by Y-chromosome and mitochondrial DNA analysis. Molecular Biology and Evolution 26: 1865-1877.
52. Hill C, Soares P, Mormina M, Macaulay V, Meehan W, Blackburn J, Clarke D, Raja JM, Ismail P, Bulbeck D, Oppenheimer S, Richards M (2006) Phylogeography and ethnogenesis of aboriginal Southeast Asians. Molecular Biology and Evolution 23: 2480-2491.
53. Macaulay V, Hill C, Achilli A, Rengo C, Clarke D, Meehan W, Blackburn J, Semino O, Scozzari R, Cruciani F, Taha A, Shaari NK, Raja JM, Ismail P, Zainuddin Z, Goodwin W, Bulbeck D, Bandelt H-J, Oppenheimer S, Torroni A, Richards M (2005) Single, rapid coastal settlement of Asia revealed by analysis of complete mitochondrial genomes. Science 308: 1034-1036.
54. Redd AJ, Stoneking M (1999) Peopling of Sahul: mtDNA variation in Aboriginal Australian and Papua New Guinean populations. American Journal of Human Genetics 65: 808-828.
55. Tabbada KA, Trejaut J, Loo JH, Chen YM, Lin M, Mirazón-Lahr M, Kivisild T, De Ungria MCA (2010) Philippine mitochondrial DNA diversity: A populated viaduct between Taiwan and Indonesia? Molecular Biology and Evolution 27: 21-31.
56. Thangaraj K, Singh L, Reddy AG, Rao VR, Sehgal SC, Underhill PA, Pierson M, Frame IG, Hagelberg E (2003) Genetic affinities of the Andaman Islanders, a vanishing human population. Current Biology 13: 86-93.
57. Van Holst Pellekaan SM, Frommer M, Sved JA, Boettcher B (1998) Mitochondrial control-region sequence variation in aboriginal Australians. American Journal of Human Genetics 62: 435-449.
58. Hudjashov G, Kivisild T, Underhill PA, Endicott P, Sanchez JJ, Lin AA, Shen P, Oefner P, Renfrew C, Villems R, Forster P (2007) Revealing the prehistoric settlement of Australia by Y chromosome and mtDNA analysis. Proceedings of the National Academy of Sciences of the United States of America 104: 8726-8730.
59. Zainuddin Z, Goodwin W (2004) Mitochondrial DNA profiling of modern Malay and Orang Asli populations in peninsular Malaysia. International Congress Series 1261: 428-430.
60. Nur Haslindawaty AR, Panneerchelvam S, Edinur HA, Norazmi MN, Zafarina Z (2010) Sequence polymorphisms of mtDNA HV1, HV2, and HV3 regions in the Malay population of Peninsular Malaysia. International Journal of Legal Medicine 124: 415-426.
61. Maruyama S, Nohira-Koike C, Minaguchi K, Nambiar P (2010) MtDNA control region sequence polymorphisms and phylogenetic analysis of Malay population living in or around Kuala Lumpur in Malaysia. International Journal of Legal Medicine 124: 165-170.

62. Wise CA, Sullivan SG, Black ML, Erber WN, Bittles AH (2005) Y-chromosome and mitochondrial DNA studies on the population structure of the Christmas Island Community. American Journal of Physical Anthropology 128: 670-677.
63. Ricaut FX, Thomas T, Arganini C, Staughton J, Leavesley M, Bellatti M, Foley R, Lahr MM (2008) Mitochondrial DNA variation in Karkar Islanders. Annals of Human Genetics 72: 349-367.
64. Vilar MG, Kaneko A, Hombhanje FW, Tsukahara T, Hwaihwanje I, Lum JK (2008) Reconstructing the origin of the Lapita Cultural Complex: mtDNA analyses of East Sepik Province, PNG. Journal of Human Genetics 53: 698-708.
65. Ohashi J, Naka I, Tokunaga K, Inaoka T, Ataka Y, Nakazawa M, Matsumura Y, Ohtsuka R (2006) Brief communication: Mitochondrial DNA variation suggests extensive gene flow from Polynesian ancestors to indigenous Melanesiens in the northwestern Bismarck Archipelago. American Journal of Physical Anthropology 130: 551-556.
66. Friedlaender JS, Friedlaender FR, Hodgson JA, Stoltz M, Koki G, Horvat G, Zhadanov S, Schurr TG, Merriwether DA (2007) Melanesian mtDNA complexity. PLoS ONE 2: e248.
67. Hagelberg E, Goldman N, Lió P, Whelan S, Schiefenhövel W, Clegg JB, Bowden DK (1999) Evidence for mitochondrial DNA recombination in a human population of island Melanesia. Proceedings of the Royal Society B: Biological Sciences 266: 485-492.
68. Deguilloux MF, Pemonge MH, Dubut V, Hänni C, Hughes S, Chollet L, Conte E, Murail P (2011) Human ancient and extant mtDNA from the Gambier Islands (French polynesia): Evidence for an early Melanesian maternal contribution and new perspectives into the settlement of Easternmost Polynesia. American Journal of Physical Anthropology 144: 248-257.
69. Sánchez-Diz P, Alves C, Carvalho E, Carvalho M, Espinheira R, García O, Pinheiro MF, Pontes L, Porto MJ, Santapa O, Silva C, Sumita D, Valente S, Whittle M, Yurrebaso I, Carracedo A, Amorim A, Gusmão L (2008) Population and segregation data on 17 Y-STRs: results of a GEP-ISFG collaborative study. International Journal of Legal Medicine 122: 529-533. doi: 10.1007/s00414-008-0265-z
70. White PS, Tatum OL, Deaven LL, Longmire JL (1999) New, Male-Specific Microsatellite Markers from the Human Y Chromosome. Genomics 57: 433-437.
71. Ayub Q, Mohyuddin A, Qamar R, Mazhar K, Zerjal T, Mehdi SQ, Tyler-Smith C (2000) Identification and characterisation of novel human Y-chromosomal microsatellites from sequence database information. Nucleic Acids Research 28: e8. doi: 10.1093/nar/28.2.e8
72. Tanaka M, Cabrera VM, González AM, Larruga JM, Takeyasu T, Fuku N, Guo LJ, Hirose R, Fujita Y, Kurata M, Shinoda KI, Umetsu K, Yamada Y, Oshida Y, Sato Y, Hattori N, Mizuno Y, Arai Y, Hirose N, Ohta S, Ogawa O, Tanaka Y, Kawamori R, Shamoto-Nagai M, Maruyama W, Shimokata H, Suzuki R, Shimodaira H (2004) Mitochondrial genome variation in Eastern Asia and the peopling of Japan. Genome Research 14: 1832-1850.
73. Bilal E, Rabadan R, Alexe G, Fuku N, Ueno H, Nishigaki Y, Fujita Y, Ito M, Arai Y, Hirose N, Ruckenstein A, Bhanot G, Tanaka M (2008) Mitochondrial DNA haplogroup D4a is a marker for extreme longevity in Japan. PLoS ONE 3: e2421.
74. Jinam TA, Hong LC, Phipps ME, Stoneking M, Ameen M, Edo J, Saitou N (2012) Evolutionary history of continental southeast asians: Early train hypothesis based on genetic analysis of mitochondrial and autosomal DNA data. Molecular Biology and Evolution 29: 3513-3527.
75. Kong QP, Yao YG, Sun C, Bandelt H-J, Zhu CL, Zhang YP (2003) Phylogeny of East Asian mitochondrial DNA lineages inferred from complete sequences. American Journal of Human Genetics 73: 671-676.
76. Ingman M, Gyllensten U (2003) Mitochondrial genome variation and evolutionary history of Australian and New Guinean aborigines. Genome Research 13: 1600-1606.

77. Kong QP, Bandelt H-J, Sun C, Yao YG, Salas A, Achilli A, Wang CY, Zhong L, Zhu CL, Wu SF, Torroni A, Zhang YP (2006) Updating the East Asian mtDNA phylogeny: A prerequisite for the identification of pathogenic mutations. Human Molecular Genetics 15: 2076-2086.
78. Pierson MJ, Martinez-Arias R, Holland BR, Gemmell NJ, Hurles ME, Penny D (2006) Deciphering past human population movements in Oceania: Provably optimal trees of 127 mtDNA genomes. Molecular Biology and Evolution 23: 1966-1975.
79. Derenko M, Malyarchuk B, Grzybowski T, Denisova G, Dambueva I, Perkova M, Dorzhu C, Luzina F, Hong KL, Vanecek T, Villems R, Zakharov I (2007) Phylogeographic analysis of mitochondrial DNA in northern Asian populations. American Journal of Human Genetics 81: 1025-1041.
80. Ingman M, Gyllensten U (2007) Rate variation between mitochondrial domains and adaptive evolution in humans. Human Molecular Genetics 16: 2281-2287.
81. Hartmann A, Thieme M, Nanduri LK, Stempfl T, Moehle C, Kivisild T, Oefner PJ (2009) Validation of microarray-based resequencing of 93 worldwide mitochondrial genomes. Human Mutation 30: 115-122.
82. Ji F, Sharpley MS, Derbeneva O, Alves LS, Qian P, Wang Y, Chalkia D, Lvova M, Xu J, Yao W, Simon M, Platt J, Xu S, Angelin A, Davila A, Huang T, Wang PH, Chuang LM, Moore LG, Qian G, Wallace DC (2012) Mitochondrial DNA variant associated with Leber hereditary optic neuropathy and high-altitude Tibetans. Proceedings of the National Academy of Sciences of the United States of America 109: 7391-7396.
83. Malyarchuk B, Derenko M, Denisova G, Kravtsova O (2010) Mitogenomic diversity in Tatars from the Volga-Ural region of Russia. Molecular Biology and Evolution 27: 2220-2226.
84. Wang CY, Li H, Hao XD, Liu J, Wang JX, Wang WZ, Kong QP, Zhang YP (2011) Uncovering the profile of somatic mtDNA mutations in Chinese Colorectal cancer patients. PLoS ONE 6: e21613.
85. Gunnarsdo ED, Li M, Bauchet M, Finstermeier K, Stoneking M (2011) High-throughput sequencing of complete human mtDNA genomes from the Philippines. Genome Research 21: 1-11.
86. Zheng HX, Yan S, Qin ZD, Wang Y, Tan JZ, Li H, Jin L (2011) Major population expansion of East Asians began before neolithic time: Evidence of mtDNA genomes. PLoS ONE 6: e25835.
87. Altshuler DM, Durbin RM, Abecasis GR, Bentley DR, Chakravarti A, Clark AG, Donnelly P, Eichler EE, Flicek P, Gabriel SB, Gibbs RA, Green ED, Hurles ME, Knoppers BM, Korbel JO, Lander ES, Lee C, Lehrach H, Mardis ER, Marth GT, McVean GA, Nickerson DA, Schmidt JP, Sherry ST, Wang J, Wilson RK, Dinh H, Kovar C, Lee S, Lewis L, Muzny D, Reid J, Wang M, Fang X, Guo X, Jian M, Jiang H, Jin X, Li G, Li J, Li Y, Li Z, Liu X, Lu Y, Ma X, Su Z, Tai S, Tang M, Wang B, Wang G, Wu H, Wu R, Yin Y, Zhang W, Zhao J, Zhao M, Zheng X, Zhou Y, Gupta N, Clarke L, Leinonen R, Smith RE, Zheng-Bradley X, Grocock R, Humphray S, James T, Kingsbury Z, Sudbrak R, Albrecht MW, Amstislavskiy VS, Borodina TA, Lienhard M, Mertes F, Sultan M, Timmermann B, Yaspo ML, Fulton L, Fulton R, Weinstock GM, Balasubramaniam S, Burton J, Danecek P, Keane TM, Kolb-Kokocinski A, McCarthy S, Stalker J, Quail M, Davies CJ, Gollub J, Webster T, Wong B, Zhan Y, Auton A, Yu F, Bainbridge M, Challis D, Evani US, Lu J, Nagaswamy U, Sabo A, et al. (2012) An integrated map of genetic variation from 1,092 human genomes. Nature 491: 56-65.
88. Kong QP, Sun C, Wang HW, Zhao M, Wang WZ, Zhong L, Hao XD, Pan H, Wang SY, Cheng YT, Zhu CL, Wu SF, Liu LN, Jin JQ, Yao YG, Zhang YP (2011) Large-Scale mtDNA screening reveals a surprising matrilineal complexity in East Asia and Its implications to the peopling of the region. Molecular Biology and Evolution 28: 513-522.

89. Loo JH, Trejaut JA, Yen JC, Chen ZS, Lee CL, Lin M (2011) Genetic affinities between the Yami tribe people of Orchid Island and the Philippine Islanders of the Batanes archipelago. BMC Genetics 12: 21.
90. Yang X, Wang X, Yao H, Deng J, Jiang Q, Guo Y, Lan G, Liao DJ, Jiang H (2012) Mitochondrial DNA polymorphisms are associated with the longevity in the Guangxi Bama population of China. Molecular Biology Reports 39: 9123-9131.
91. Gunnarsdóttir ED, Nandineni MR, Li M, Myles S, Gil D, Pakendorf B, Stoneking M (2011) Larger mitochondrial DNA than Y-chromosome differences between matrilocal and patrilocal groups from Sumatra. Nature Communications 2: 228.
92. Schönberg A, Theunert C, Li M, Stoneking M, Nasidze I (2011) High-throughput sequencing of complete human mtDNA genomes from the Caucasus and West Asia: High diversity and demographic inferences. European Journal of Human Genetics 19: 988-994.
93. Peng MS, He JD, Liu HX, Zhang YP (2011) Tracing the legacy of the early Hainan Islanders - A perspective from mitochondrial DNA. BMC Evolutionary Biology 11: 46.
94. Behar DM, Van Oven M, Rosset S, Metspalu M, Loogväli EL, Silva NM, Kivisild T, Torroni A, Villems R (2012) A "copernican" reassessment of the human mitochondrial DNA tree from its root. American Journal of Human Genetics 90: 675-684.
95. Delfin F, Min-Shan Ko A, Li M, Gunnarsdóttir ED, Tabbada KA, Salvador JM, Calacal GC, Sagum MS, Datar FA, Padilla SG, De Ungria MCA, Stoneking M (2014) Complete mtDNA genomes of Filipino ethnolinguistic groups: A melting pot of recent and ancient lineages in the Asia-Pacific region. European Journal of Human Genetics 22: 228-237.
96. Ko AMS, Chen CY, Fu Q, Delfin F, Li M, Chiu HL, Stoneking M, Ko YC (2014) Early Austronesians: Into and out of Taiwan. American Journal of Human Genetics 94: 426-436.
97. Loo J-H, Trejaut J, Yen J-C, Chen Z-S, Ng W-M, Huang C-Y, Hsu K-N, Hung K-H, Hsiao Y, Wei Y-H, Lin M (2014) Mitochondrial DNA association study of type 2 diabetes with or without ischemic stroke in Taiwan. BMC Research Notes 7: 223.
98. Duggan AT, Evans B, Friedlaender FR, Friedlaender JS, Koki G, Merriwether DA, Kayser M, Stoneking M (2014) Maternal history of oceania from complete mtDNA genomes: Contrasting ancient diversity with recent homogenization due to the Austronesian expansion. American Journal of Human Genetics 94: 721-733.
99. Wong L-P, Ong Rick T-H, Poh W-T, Liu X, Chen P, Li R, Lam Kevin K-Y, Pillai Nisha E, Sim K-S, Xu H, Sim N-L, Teo S-M, Foo J-N, Tan Linda W-L, Lim Y, Koo S-H, Gan Linda S-H, Cheng C-Y, Wee S, Yap Eric P-H, Ng Pauline C, Lim W-Y, Soong R, Wenk Markus R, Aung T, Wong T-Y, Khor C-C, Little P, Chia K-S, Teo Y-Y (2013) Deep Whole-Genome Sequencing of 100 Southeast Asian Malays. The American Journal of Human Genetics 92: 52-66.
100. Ingman M, Kaessmann H, Pääbo S, Gyllensten U (2000) Mitochondrial genome variation and the origin of modem humans. Nature 408: 708-713.
101. Soares P, Trejaut JA, Loo JH, Hill C, Mormina M, Lee CL, Chen YM, Hudjashov G, Forster P, Macaulay V, Bulbeck D, Oppenheimer S, Lin M, Richards MB (2008) Climate change and postglacial human dispersals in Southeast Asia. Molecular Biology and Evolution 25: 1209-1218.
102. Chandrasekar A, Kumar S, Sreenath J, Sarkar BN, Urade BP, Mallick S, Bandopadhyay SS, Barua P, Barik SS, Basu D, Kiran U, Gangopadhyay P, Sahani R, Prasad BVR, Gangopadhyay S, Lakshmi GR, Ravuri RR, Padmaja K, Venugopal PN, Sharma MB, Rao VR (2009) Updating Phylogeny of Mitochondrial DNA Macrohaplogroup M in India: Dispersal of Modern Human in South Asian Corridor. PLoS ONE 4: e0007447.
103. Peng MS, Palanichamy MG, Yao YG, Mitra B, Cheng YT, Zhao M, Liu J, Wang HW, Pan H, Wang WZ, Zhang AM, Zhang W, Wang D, Zou Y, Yang Y, Chaudhuri TK, Kong QP, Zhang

YP (2011) Inland post-glacial dispersal in East Asia revealed by mitochondrial haplogroup M9a'b. BMC Biology 9:2.
104. Qin Z, Yang Y, Kang L, Yan S, Cho K, Cai X, Lu Y, Zheng H, Zhu D, Fei D, Li S, Jin L, Li H (2010) A mitochondrial revelation of early human migrations to the Tibetan Plateau before and after the last glacial maximum. American Journal of Physical Anthropology 143: 555-569.
105. Reiff DM, Spathis R, Chan CW, Vilar MG, Sankaranarayanan K, Lynch D, Ehrlich E, Kerath S, Chowdhury R, Robinowitz L, Koji Lum J, Garruto RM (2011) Inherited and somatic mitochondrial DNA mutations in Guam amyotrophic lateral sclerosis and parkinsonism-dementia. Neurological Sciences 32: 883-892.
106. Derenko M, Malyarchuk B, Denisova G, Perkova M, Rogalla U, Grzybowski T, Khusnutdinova E, Dambueva I, Zakharov I (2012) Complete mitochondrial DNA analysis of eastern eurasian haplogroups rarely found in populations of Northern Asia and Eastern Europe. PLoS ONE 7: e32179.
107. Kazuno AA, Munakata K, Nagai T, Shimozono S, Tanaka M, Yoneda M, Kato N, Miyawaki A, Kato T (2006) Identification of mitochondrial DNA polymorphisms that alter mitochondrial matrix pH and intracellular calcium dynamics. PLoS Genetics 2: 1167-1177.
108. Mishmar D, Ruiz-Pesini E, Golik P, Macaulay V, Clark AG, Hosseini S, Brandon M, Easleyf K, Chen E, Brown MD, Sukernik RI, Olckers A, Wallace DC (2003) Natural selection shaped regional mtDNA variation in humans. Proceedings of the National Academy of Sciences of the United States of America 100: 171-176.
109. Benton M, Macartney-Coxson D, Eccles D, Griffiths L, Chambers G, Lea R (2012) Complete mitochondrial genome sequencing reveals novel haplotypes in a polynesian population. PLoS ONE 7: e35026.
110. Razafindrazaka H, Ricaut FX, Cox MP, Mormina M, Dugoujon JM, Randriamarolaza LP, Guitard E, Tonasso L, Ludes B, Crubézy E (2010) Complete mitochondrial DNA sequences provide new insights into the Polynesian motif and the peopling of Madagascar. European Journal of Human Genetics 18: 575-581.
111. Corser CA, McLenachan PA, Pierson MJ, Harrison GLA, Penny D (2012) The Q2 Mitochondrial Haplogroup in Oceania. PLoS ONE 7: e52022.
112. Soares P, Rito T, Trejaut J, Mormina M, Hill C, Tinkler-Hundal E, Braid M, Clarke DJ, Loo JH, Thomson N, Denham T, Donohue M, Macaulay V, Lin M, Oppenheimer S, Richards MB (2011) Ancient voyaging and polynesian origins. American Journal of Human Genetics 88: 239-247.
113. Knapp M, Horsburgh KA, Prost S, Stanton JA, Buckley HR, Walter RK, Matisoo-Smith EA (2012) Complete mitochondrial DNA genome sequences from the first New Zealanders. Proceedings of the National Academy of Sciences of the United States of America 109: 18350-18354.
114. Duggan AT, Stoneking M (2013) A highly unstable recent mutation in human mtDNA. American Journal of Human Genetics 92: 279-284.
115. Palanichamy MG, Sun C, Agrawal S, Bandelt H-J, Kong QP, Khan F, Wang CY, Chaudhuri TK, Palla V, Zhang YP (2004) Phylogeny of mitochondrial DNA macrohaplogroup N in India, based on complete sequencing: Implications for the peopling of South Asia. American Journal of Human Genetics 75: 966-978.
116. Achilli A, Rengo C, Battaglia V, Pala M, Olivieri A, Fornarino S, Magri C, Scozzari R, Babudri N, Santachiara-Benerecetti AS, Bandelt H-J, Semino O, Torroni A (2005) Saami and Berbers - An unexpected mitochondrial DNA link. American Journal of Human Genetics 76: 883-886.
117. González AM, García O, Larruga JM, Cabrera VM (2006) The mitochondrial lineage U8a reveals a Paleolithic settlement in the Basque country. BMC Genomics 7: 124.

118. Just RS, Diegoli TM, Saunier JL, Irwin JA, Parsons TJ (2008) Complete mitochondrial genome sequences for 265 African American and U.S. "Hispanic" individuals. Forensic Science International: Genetics 2: e45-e48.
119. Torroni A, Achilli A, Macaulay V, Richards M, Bandelt H-J (2006) Harvesting the fruit of the human mtDNA tree. Trends in Genetics 22: 339-345.
120. Malyarchuk B, Grzybowski T, Derenko M, Perkova M, Vanecek T, Lazur J, Gomolcak P, Tsybovsky I (2008) Mitochondrial DNA phylogeny in eastern and western Slavs. Molecular Biology and Evolution 25: 1651-1658.
121. Behar DM, Metspalu E, Kivisilid T, Rosset S, Tzur S, Hadid Y, Yudkovsky G, Rosengarten D, Pereira L, Amorim A, Kutuev I, Gurwitz D, Bonne-Tamir B, Villems R, Skorecki K (2008) Counting the founders: The matrilineal genetic ancestry of the Jewish Diaspora. PLoS ONE 3: e2062.
122. Behar DM, Villems R, Soodyall H, Blue-Smith J, Pereira L, Metspalu E, Scozzari R, Makkan H, Tzur S, Comas D, Bertranpetit J, Quintana-Murci L, Tyler-Smith C, Wells RS, Rosset S (2008) The Dawn of Human Matrilineal Diversity. American Journal of Human Genetics 82: 1130-1140.
123. Quintana-Murci L, Quach H, Harmant C, Luca F, Massonnet B, Patin E, Sica L, Mouguiama-Daouda P, Comas D, Tzur S, Balanovsky O, Kidd KK, Kidd JR, Van Der Veen L, Hombert JM, Gessain A, Verdu P, Froment A, Bahuchet S, Heyer E, Dausset J, Salas A, Behar DM (2008) Maternal traces of deep common ancestry and asymmetric gene flow between Pygmy hunter-gatherers and Bantu-speaking farmers. Proceedings of the National Academy of Sciences of the United States of America 105: 1596-1601.
124. Achilli A, Perego UA, Bravi CM, Coble MD, Kong QP, Woodward SR, Salas A, Torroni A, Bandelt H-J (2008) The phylogeny of the four pan-American MtDNA haplogroups: Implications for evolutionary and disease studies. PLoS ONE 3: e1764.
125. Kujanová M, Pereira L, Fernandes V, Pereira JB, Černý V (2009) Near eastern neolithic genetic input in a small oasis of the Egyptian Western Desert. American Journal of Physical Anthropology 140: 336-346.
126. Chaubey G, Karmin M, Metspalu E, Metspalu M, Selvi-Rani D, Singh VK, Parik J, Solnik A, Naidu BP, Kumar A, Adarsh N, Mallick CB, Trivedi B, Prakash S, Reddy R, Shukla P, Bhagat S, Verma S, Vasnik S, Khan I, Barwa A, Sahoo D, Sharma A, Rashid M, Chandra V, Reddy AG, Torroni A, Foley RA, Thangaraj K, Singh L, Kivisild T, Villems R (2008) Phylogeography of mtDNA haplogroup R7 in the Indian peninsula. BMC Evolutionary Biology 8: 227.
127. Kumar S, Ravuri RR, Koneru P, Urade B, Sarkar B, Chandrasekar A, Rao VR (2009) Reconstructing Indian-Australian phylogenetic link. BMC Evolutionary Biology 9: 173.
128. Costa MD, Cherni L, Fernandes V, Freitas F, Ammar el Gaaied AB, Pereira L (2009) Data from complete mtDNA sequencing of Tunisian centenarians: Testing haplogroup association and the "golden mean" to longevity. Mechanisms of Ageing and Development 130: 222-226.
129. Cerný V, Fernandes V, Costa MD, Hájek M, Mulligan CJ, Pereira L (2009) Migration of Chadic speaking pastoralists within Africa based on population structure of Chad Basin and phylogeography of mitochondrial L3f haplogroup. BMC Evolutionary Biology 9: 63.
130. Derenko M, Malyarchuk B, Grzybowski T, Denisova G, Rogalla U, Perkova M, Dambueva I, Zakharov I (2010) Origin and post-glacial dispersal of mitochondrial DNA haplogroups C and D in Northern Asia. PLoS ONE 5: e15214.
131. Cerný V, Mulligan CJ, Fernandes V, Silva NM, Alshamali F, Non A, Harich N, Cherni L, El Gaaied ABA, Al-Meeri A, Pereira L (2011) Internal diversification of mitochondrial haplogroup r0a reveals post-last glacial maximum demographic expansions in South Arabia. Molecular Biology and Evolution 28: 71-78.
132. Batini C, Lopes J, Behar DM, Calafell F, Jorde LB, Van Der Veen L, Quintana-Murci L, Spedini G, Destro-Bisol G, Comas D (2011) Insights into the demographic history of

African pygmies from complete mitochondrial genomes. Molecular Biology and Evolution 28: 1099-1110.
133. Kumar S, Bellis C, Zlojutro M, Melton PE, Blangero J, Curran JE (2011) Large scale mitochondrial sequencing in Mexican Americans suggests a reappraisal of Native American origins. BMC Evolutionary Biology 11: 293.
134. Soares P, Alshamali F, Pereira JB, Fernandes V, Silva NM, Afonso C, Costa MD, Musilová E, Macaulay V, Richards MB, Černý V, Pereira L (2012) The expansion of mtDNA haplogroup L3 within and out of Africa. Molecular Biology and Evolution 29: 915-927.
135. Barbieri C, Whitten M, Beyer K, Schreiber H, Li M, Pakendorf B (2012) Contrasting maternal and paternal histories in the linguistic context of Burkina Faso. Molecular Biology and Evolution 29: 1213-1223.
136. Fu Q, Meyer M, Gao X, Stenzel U, Burbano HA, Kelso J, Pääbo S (2013) DNA analysis of an early modern human from Tianyuan Cave, China. Proceedings of the National Academy of Sciences of the United States of America 110: 2223-2227.
137. Fu Q, Mittnik A, Johnson PLF, Bos K, Lari M, Bollongino R, Sun C, Giemsch L, Schmitz R, Burger J, Ronchitelli AM, Martini F, Cremonesi RG, Svoboda J, Bauer P, Caramelli D, Castellano S, Reich D, Pääbo S, Krause J (2013) A revised timescale for human evolution based on ancient mitochondrial genomes. Current Biology 23: 553-559.
138. Derenko M, Malyarchuk B, Bahmanimehr A, Denisova G, Perkova M, Farjadian S, Yepiskoposyan L (2013) Complete mitochondrial DNA diversity in Iranians. PLoS ONE 8: e80673.
139. Richards M, Macaulay V, Hickey E, Vega E, Sykes B, Guida V, Rengo C, Sellitto D, Cruciani F, Kivisild T, Villems R, Thomas M, Rychkov S, Rychkov O, Rychkov Y, Golge M, Dimitrov D, Hill E, Bradley D, Romano V, Cali F, Vona G, Demaine A, Papiha S, Triantaphyllidis C, Stefanescu G, Hatina J, Belledi M, Di Rienzo A, Novelletto A, Oppenheim A, Norby S, Al-Zaheri N, Santachiara-Benerecetti S, Scozzari R, Torroni A, Bandelt H-J (2000) Tracing European founder lineages in the Near Eastern mtDNA pool. American Journal of Human Genetics 67: 1251-1276.
140. Mellars P, Gori KC, Carr M, Soares PA, Richards MB (2013) Genetic and archaeological perspectives on the initial modern human colonization of southern Asia. Proceedings of the National Academy of Sciences of the United States of America 110: 10699-10704.
